# *Escherichia coli* attachment to model particulates: The effects of bacterial cell characteristics and particulate properties

**DOI:** 10.1371/journal.pone.0184664

**Published:** 2017-09-14

**Authors:** Xiao Liang, Chunyu Liao, Michelle L. Soupir, Laura R. Jarboe, Michael L. Thompson, Philip M. Dixon

**Affiliations:** 1 Department of Agricultural and Biosystems Engineering, Iowa State University, Ames, Iowa, United States of America; 2 Department of Microbiology, Iowa State University, Ames, Iowa, United States of America; 3 Department of Chemical and Biological Engineering, Iowa State University, Ames, Iowa, United States of America; 4 Department of Agronomy, Iowa State University, Ames, Iowa, United States of America; 5 Department of Statistics, Iowa State University, Ames, Iowa, United States of America; Purdue University, UNITED STATES

## Abstract

*E*. *coli* bacteria move in streams freely in a planktonic state or attached to suspended particulates. Attachment is a dynamic process, and the fraction of attached microorganisms is thought to be affected by both bacterial characteristics and particulate properties. In this study, we investigated how the properties of cell surfaces and stream particulates influence attachment. Attachment assays were conducted for 77 *E*. *coli* strains and three model particulates (ferrihydrite, Ca-montmorillonite, or corn stover) under environmentally relevant conditions. Surface area, particle size distribution, and total carbon content were determined for each type of particulate. Among the three particulates, attachment fractions to corn stover were significantly larger than the attachments to 2-line ferrihydrite (*p*-value = 0.0036) and Ca-montmorillonite (*p*-value = 0.022). Furthermore, attachment to Ca-montmorillonite and corn stover was successfully modeled by a Generalized Additive Model (GAM) using cell characteristics as predictor variables. The natural logarithm of the net charge on the bacterial surface had a significant, positive, and linear impact on the attachment of *E*. *coli* bacteria to Ca-montmorillonite (*p*-value = 0.013), but it did not significantly impact the attachment to corn stover (*p*-value = 0.36). The large diversities in cell characteristics among 77 *E*. *coli* strains, particulate properties, and attachment fractions clearly demonstrated the inadequacy of using a static parameter or linear coefficient to predict the attachment behavior of *E*. *coli* in stream water quality models.

## Introduction

Pathogens are the leading cause of water quality impairments in rivers and streams in the United States. Currently, 178,048 miles of assessed rivers and streams in the US are contaminated due to elevated levels of pathogens or pathogen indicators [1]. Although not all *E*. *coli* and enterococcus strains are pathogenic, *E*. *coli* and enterococci are used as indicators to predict when a risk to human health is present in fresh or marine water, respectively [2]. Therefore, improved understanding of *E*. *coli* fate and transport in the environment is needed. In streams, microorganisms may move freely in water in a planktonic state or they may be attached to suspended inorganic and organic particles [3–8]. To model bacterial fate and transport, the fraction of *E*. *coli* that are attached to suspended particles needs to be estimated [9]. Previously, the fraction of attached *E*. *coli* has been estimated as a static parameter [10], or it has been predicted based on a linear correlation of planktonic *E*. *coli* with the concentration of suspended clay [11, 12]. However, bacterial attachment to particulates in aquatic environments is a dynamic process, affected by bacterial characteristics, particulate properties, and environmental factors.

Bacterial surface characteristics, such as net charge, hydrophobicity, and extracellular polymeric substances (EPS) can impact bacterial attachment to particulates [13, 14]. *E*. *coli* cells typically have a net negative charge due to the carboxylate and phosphate groups present in peptidoglycan and lipopolysaccharides that compose the cell walls. The surface charge can impact attachment to particles by repulsion of similarly charged particles or by attraction to particulates with an opposite charge [15, 16]. Surface hydrophobicity also affects attachment. Hydrophobicity is determined by the functional groups of both extracellular residues and structures on the surface of the cell; these can be either dominantly hydrophilic or dominantly hydrophobic [17]. Some studies have found a positive correlation between bacterial attachment to particles and hydrophobicity [18–22]. On this basis, it might be assumed that hydrophobicity of the bacterial surface would also regulate the transport of cells through soil materials. However, neither Gannon et al. [23] nor Bolster et al. [16] reported a correlation between measured hydrophobicity and transport of a variety of bacterial strains through soil materials or packed quartz beds, respectively.

Extracellular polymeric substances (EPS) are mainly composed of polysaccharides and proteins, but they may also include other macromolecules such as DNA, lipids, and humic-like substances [24]. EPS play an important role in cell aggregation, cell adhesion, biofilm formation, and protection of cells from hostile environments [25]. The polysaccharide / protein ratio of EPS is positively correlated with cell surface charge [26], and the presence of cellulose in EPS of laboratory strains of *E*. *coli* has been found essential for attachment to plastic surfaces [27]. Omoike et al. [28] and Parikh and Chorover [29] have demonstrated the potential for phosphate/phosphonate and phosphodiester groups of EPS to form strong inner-sphere bonds with Fe oxide surfaces at pH 6–7. On the other hand, researchers have found no correlation between the presence of three EPS-associated genes (*ompC*, *slp*, *and surA*) and attachment to quartz [30].

Both size and shape of cell can impact *E*. *coli* attachment. Attachment decreases the total surface energy of the cell-particle system in the aquatic environment by forming contact regions with lower surface energy. Surface energy is the excess energy per unit surface area and larger or highly spherical bacteria have lower surface energy. Therefore, smaller or non-spherical bacteria have a higher preference of attaching to particles than larger or more highly spherical bacteria [31]. Consequently, the fraction of bacteria attached to particles is typically negatively correlated with particle size [32–38].

Previous research has also reported that the survival time of bacteria in water and soil can range from a few days to several months [39]. The survival can be influenced by pH, temperature, nutrient availability, and competition from other microorganisms [40]. Attachment also increases the survival of cells. Burton et al. [41] reported increased survival of *E*. *coli* in freshwater sediments, with higher survival when clay content increased. The populations of bacteria surviving in bottom sediments are protected from ultraviolet radiation [3, 42]. It has also been reported that *E*. *coli* survival in water bodies increases after attaching to mineral aggregates with high sediment organic matter (SOM) concentration [43].

Given the importance of understanding *E*. *coli* transport in the environment, we explored relationships between particulate surface and cell surface properties and attachment. The objectives of this study were to: 1) identify the impact of particulate properties on *E*. *coli* attachment fractions; 2) identify cell characteristics important for predicting *E*. *coli* particulate attachment; and 3) construct and evaluate statistical models for predicting *E*. *coli* particulate attachment fractions. The particulate properties considered were size, surface area, and organic carbon content, and the cell properties included hydrophobicity, surface charge, zeta potential, size, and EPS composition [44].

## Materials and methods

### *E*. *coli* strain selection and preparation

Strain collection, preparation, and rep-PCR have been described in detail previously [44]. Briefly, six times between 10/26/2012 and 6/18/2013, stream sediment and water samples were collected from two locations along Squaw Creek in Ames, Iowa, U.S.A, from public access points which do not require specific permissions to sample. The field studies did not involve endangered or protected species. Two hundred strains each were isolated from stream sediments and water samples. A pathogenic strain was also investigated: ATCC^TM^ 43888, a genetically modified version of *E*. *coli* O157:H7, with the genes that produce Shiga-like toxins I and II removed. After isolation, the strains were inoculated in Luria-Bertani liquid media (BD Biosciences; San Jose, CA), grown to the stationary phase, and stored at -80°C in 15% glycerol. Using primer (GTG)_5_ with the sequence of 5’-GTGGTGGTGGTGGTG-3’[45], we differentiated the 400 *E*. *coli* strains by rep-PCR. Forty-five sediment strains (23% of the 200 strains) and 33 water strains (17% of the 200 strains) were considered genomically distinct on the basis of a 90% similarity criterion [44]. One sediment strain (No. 122) did not grow sufficiently in M9 minimal broth (Sigma-Aldrich, St. Louis, MO), so further analyses were based on 44 sediment strains and 33 water strains.

Time to early stationary phase of the isolates was determined by absorbance at 600 nm (spectrophotometer, HACH, Loveland, CO). *E*. *coli* isolates were inoculated in 10 mL of M9 broth and incubated to early stationary phase (1.0<OD600<1.5). To harvest the cells, the *E*. *coli* suspension was centrifuged for 15 min at 4,000 rpm/1878 ×*g* (Centrifuge 5430R with Rotor F-35-6-30, Eppendorf, Hauppauge, NY) at 4°C. The supernatant was discarded, and the cells were re-suspended in CaCO_3_ solution of pH 8.0 and ionic strength 10 mM (prepared by diluting saturated CaCO_3_ solution with deionized water). The CaCO_3_ solution was used to simulate typical aquatic conditions of the Upper Midwestern United States [46]. Electrical conductivity was measured by a conductivity meter (Accumet AB30, Fisher Scientific, Asheville, NC) and ionic strength was estimated by:

$$\mathrm{Ionic}\;\mathrm{strength}\;\left({\mathrm{in}\;\mathrm{mmol}\;L}^{\text{-}1}\right)\approx 0.015\times \mathrm{Electrical}\;\mathrm{conductivity}\;\left({\mathrm{in}\;\mu s\;\mathrm{cm}}^{\text{-}1}\right)$$ (1)

### Model particulates

We conducted attachment assays with three model particulates, chosen to represent common particulates in streams of the upper Midwestern United States: an iron oxide mineral, a layer silicate mineral, and an organic matter model. Poorly crystalline iron oxides are common in stream sediments, and bacteria cells can potentially attach to them because of charge interactions. At typical stream pH values, iron oxides such as 2-line ferrihydrite will carry a net positive charge to which negatively charged bacterial cells are attracted. To prepare 2-line ferrihydrite, Fe(NO_3_)_3_·9H_2_O was added to 75°C deionized water in a water bath, and the temperature was maintained at 75°C for 10–12 minutes. After the color changed from gold to dark brown, which is the color of ferrihydrite, the container was cooled in ice water and the solution was transferred into dialysis bags, followed by dialysis and freeze-drying of the resultant solid [47].

Smectite is a ubiquitous clay–size layer silicate mineral in stream sediments of the upper Midwest. We used Ca-saturated montmorillonite (purchased from The Clay Mineral Society, Chantilly, Virginia) as a model smectite. Under typical conditions of pH and ionic strength, Ca-montmorillonite occurs as low-density, stacked-layer quasicrystals, with large, low-charge surfaces and spaces between substacks where bacterial cells can attach [48].

Last, we simulated the interactions of *E*. *coli* cells with a common constituent of stream particles, particulate organic matter (POM), by conducting experiments with ground corn stover particles. Corn stover particles are dominated by primary and secondary cell wall materials that contain cellulose (a beta-linked glucose polymer) and lignin (a biopolymer composed of irregularly linked phenol monomers). These surfaces may be recognized by *E*. *coli* attachment factors that bind to carbohydrate receptors. Dried corn stover was ground, passed through a 53-μm sieve, and stored at room temperature.

### Analysis of particulate properties

Surface area of the dry particles was determined using N_2_ by the Brunauer-Emmett-Teller (BET) method using a NOVA Surface Area Analyzer-4200 (Quantachrome Instruments, Boynton Beach, Florida). The particle size distribution was analyzed by a Mastersizer 3000 laser particle size analyzer (Malvern Instruments Ltd, Worcestershire, UK). The total carbon content of the particulate samples was determined by dry combustion using a LECO elemental analyzer (LECO Corp., St. Joseph, Michigan).

### Analysis of *E*. *coli* properties

We tested the hydrophobicity, zeta potential, EPS components, net charge at pH 8, and cell acidity for each *E*. *coli* strain. Detailed methods have been described previously [44]. In this previous publication, these *E*. *coli* property data were evaluated for differences associated with stream and sediment environments, but the paper did not consider the role of these properties in attachment to particulates. Briefly, the microbial adhesion to hydrocarbon (MATH) method was employed to determine the hydrophobicity of *E*. *coli* cells [49, 50] using dodecane as the hydrocarbon. The zeta potential (reported in mV) of an *E*. *coli* suspension with OD600 = 0.1 was measured at room temperature using a Zetasizer Nano-ZS (Malvern instruments Inc., Westborough MA). The total protein and the polysaccharide content (in µg (10^8^ cells)^-1^) of EPS was determined by using an ethanol-extraction method followed by spectrometric measurements [51–53]. Potentiometric titration of *E*. *coli* suspensions was conducted to measure the net charge (in meq (10^8^ cells)^-1^) of the bacterial surfaces [54] at pH 8 as well as total cell acidity (in meq (10^8^ cells)^-1^).

### Attachment assays

Attachment assays of *E*. *coli* to model particulates were investigated under conditions that represent typical stream environments of the upper Midwest: pH of 8, ionic strength of 10 mM, and 22°C. To be consistent with previous reports of suspended sediment concentrations, model particulate concentrations were set at 1.06 mg L^-1^ for particulate organic matter, 1.06 mg L^-1^ for Fe oxides, and 108 mg L^-1^ for silicate clay [55–58]. The concentration of *E*. *coli* was set at 10^3^ cells mL^-1^, a relatively high but reasonable value based on the past record of *E*. *coli* concentrations in Squaw Creek [9]. The chosen *E*. *coli* and particle concentrations resulted in particle: *E*. *coli* surface area ratios of 600 for samples with corn stover, 2.5×10^4^ for samples with ferrihydrite, and 2×10^6^ for samples with Ca-montmorillonite.

**Fig 1:**
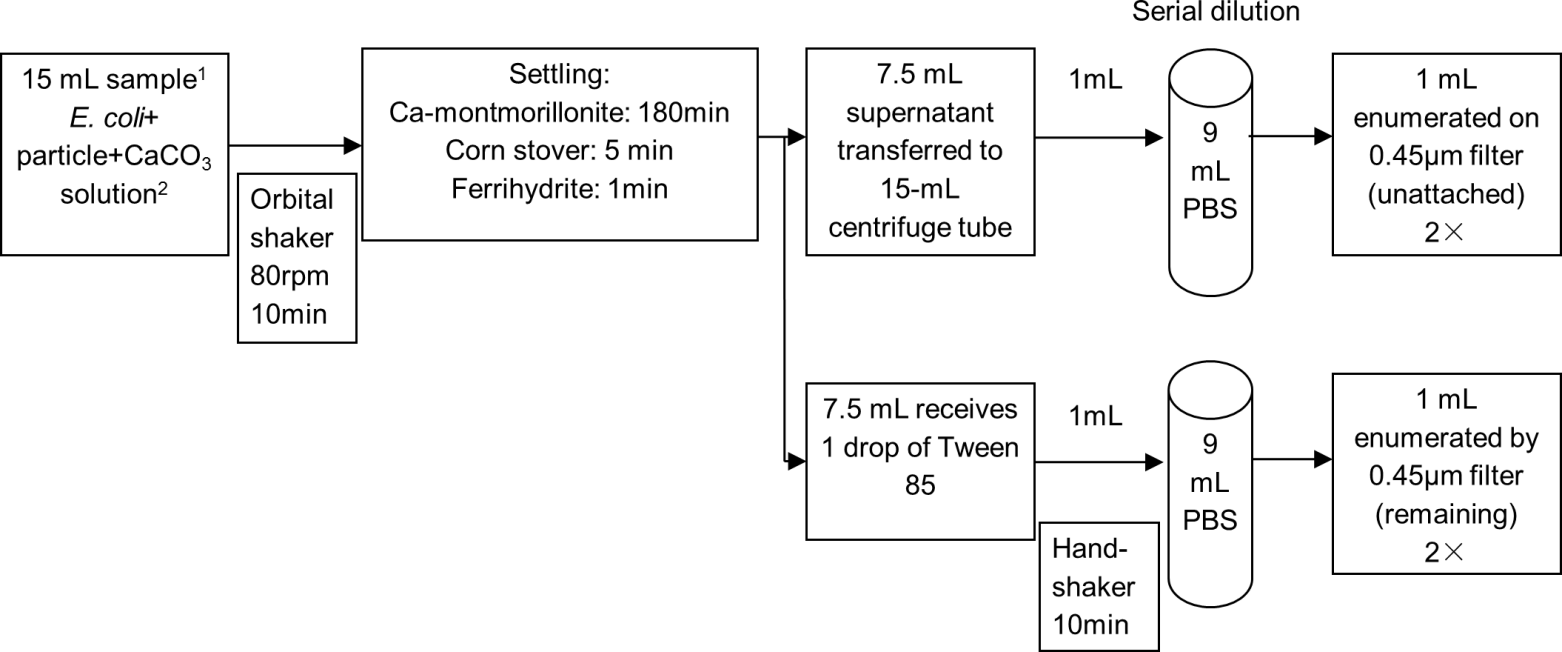
Flow chart describing the attachment assay. 1. *E*. *coli* concentration: 10^3^ cfu/ mL; Ca-montmorillonite concentration: 108 mg/ mL; Corn stover: 1.06 mg L^-1^; Ferrihydrite concentration: 1.06 mg/ mL; triplicate samples for each assay. 2. Solute: CaCO_3_; solvent: autoclaved deionized water. This solution has pH 8, with 10mM as ionic strength.

**Fig 2:**
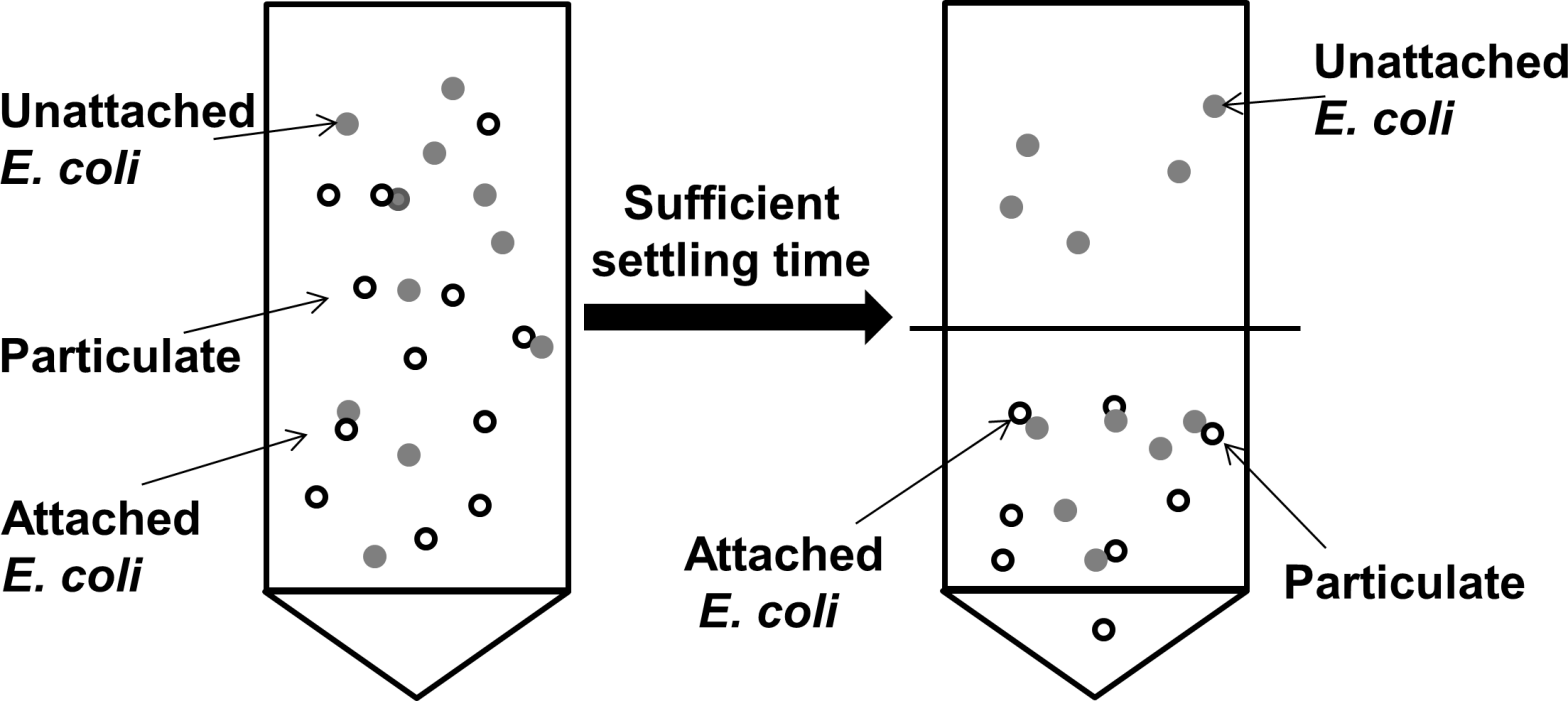
Attachment assay depiction. The concentration difference between the remaining suspension (C_r_) and the supernatant (C_s_) was considered the attached *E*. *coli* concentration.

Fig 1, modified from Liang et. al [59], shows a flow chart of the experimental procedure, which was conducted in triplicate for each strain-particle interaction. Calculated volumes of *E*. *coli* suspensions (1.0×10^3^ CFU mL^-1^), particle suspensions (at the concentrations shown above), and CaCO_3_ solution (pH of 8, ionic strength of 10 mM) were made up to 15 mL in 50- mL centrifuge tubes and the samples were shaken at 80 rpm for 10 min on an orbital shaker to enhance bacteria—particle interactions and attachment [60]. After shaking, each sample was transferred to a 15-mL centrifuge tube, and the tubes were placed vertically in racks to allow attached *E*. *coli* and particles to settle via gravity for the specified settling times as described above. For samples with Ca-montmorillonite, the samples were placed at 4°C to reduce the possibility of *E*. *coli* regrowth during settling. After the *E*. *coli* cells attached to particles, they settled out of suspension more quickly than freely suspended *E*. *coli* cells. For the 2-line ferrihydrite and corn stover particles, 1 min was sufficient for all particles to settle by visual observation. Ca-montmorillonite settled after 180 min, as reported previously [59]. After settling, 7.5 mL of supernatant, which contained the unattached bacteria, were extracted and placed in a new conical tube. Then 1 mL of supernatant was removed and serially diluted twice in 9 mL of CaCO_3_ solution. The final concentration was estimated to be within a countable range (20 to 80 colony-forming units per plate [61]), as recommended for the membrane filtration technique. Next, to estimate the bacterial concentration of remaining 7.5 mL, a combination of chemical and physical dispersion techniques were applied. One drop of Tween 85 (a polyoxyethylene non-ionic surfactant from Fisher Scientific, Fair Lawn, New Jersey) was added to the remaining 7.5 mL and shaken at 300 rpm for 10 min with a handshaker (Fisher Scientific, Asheville, NC) [62]. The serial dilution procedure was the same as described for the supernatant. The *E*. *coli* concentration (in CFU mL^-1^) in the original supernatant and in the remaining suspension were enumerated by membrane filtration in duplicate on Luria-Bertani agar. The concentration difference between the remaining suspension and the supernatant was considered the attached *E*. *coli* concentration (Fig 2). The attached fraction was calculated as

$$1-\frac{15\times C_{s}}{7.5\times (C_{r}+C_{s})}=\frac{C_{r}\text{-}C_{s}}{C_{r}+C_{s}}$$ (2)

where C_s_ is the supernatant concentration and C_r_ is the concentration in the remaining suspension. Triplicate measurements were averaged to provide a single estimate of the attached fraction for each strain-particle interaction.

Sometimes this attachment assay produced negative attachment fractions, meaning that supernatant concentration was higher than the concentration in the remaining suspension, possibly due to cell buoyancy [63]. According to Eq 2, some negative values could be due to uncertainty associated with plate counting. Plate counting of colonies is accepted by most researchers to estimate the concentration of viable bacteria in a sample. The countable range in 45-mm petri dishes is 20 to 80 colonies [61]. To assess the uncertainty in our experimental procedures, 5 *E*. *coli* strains were cultured, harvested, and diluted using the same procedures as in Fig 1. Plating was repeated 6 times for each strain. We found that the coefficient of variation (standard deviation divided by the mean) was 0.2 when the average count was 20 colonies, but the coefficient of variation decreased to 0.08 when the average was 50 colonies. Based on the results from the uncertainty analysis, 95% confidence intervals for each plate count were calculated and negative attachment fractions within the corresponding range were adjusted to zero.

In a related publication [64] additional replicates were included for a subset of strains assessed for attachment to corn stover only. In this previous publication the negative values were not adjusted to zero. The focus of this prior work was the role of outer membrane proteins in attachment to corn stover.

### Data analysis

The non-parametric Friedman test was used to compare the attached fractions among the three particulate types after adjusting for differences between *E*. *coli* strains. The non-parametric Kendall-tau correlation was calculated for combination of property and particle type. The relationship between each *E*. *coli* property and the attached fraction was visualized using LOESS (a non-parametric LOcal regrESSion model) to smooth the data. To predict the attached fraction using multiple *E*. *coli* properties, a generalized additive model (GAM) was used. The GAM is a semi-parametric extension of the generalized linear model in which the response variable depends linearly on unknown but smooth functions of predictor variables, and interest focuses on inferences about the smoothing functions. In general, the mean *μ* of a response *y* is related to an additive function of the predictors (*x*_i_) via a link function *g* [65]:

$$g(\mu )=\alpha +f_{1}(x_{1})+\ldots +f_{m}(x_{m})$$ (3)

Statistical analysis of data was performed using R project software (ver. 3.1.3, R Core Team, Vienna, Austria). The GAMs were fit using the R package mgcv (Version 1.8) with gaussian errors and a linear link function, i.e., *g*(*μ*) = *μ*. For single predictor variables, the smooth functions were penalized regression splines; tensor product smooths were used to assess potential interactions between two predictor variables. When a fitted smooth function for an *E*. *coli* property was approximately linear, the smooth function, *f(x)*, was replaced by a linear function, *βx*.

For each particulate type, models with different subsets of *E*. *coli* characteristics and their interactions were generated to fit the attached fractions. Model assumptions were checked using quantile-quantile plots of residuals, plots of residual *vs*. fitted values, and plots of observed values *vs*. fitted values. Several criteria were used to evaluate model performance. The coefficient of determination (R^2^) estimates the collinearity between observed and predicted values, and ranges between 0 (no linear relationship) and 1 (perfect linear relationship). The generalized cross validation (GCV) criterion was used to evaluate the models based on their goodness of fit. The chi-square test was used to assess the statistical significant of individual terms of a smooth. The preferred model was the one with the highest R^2^ and smallest GCV.

## Results

### Particulate properties and *E*. *coli*—Particulate attachment results

**Fig 3:**
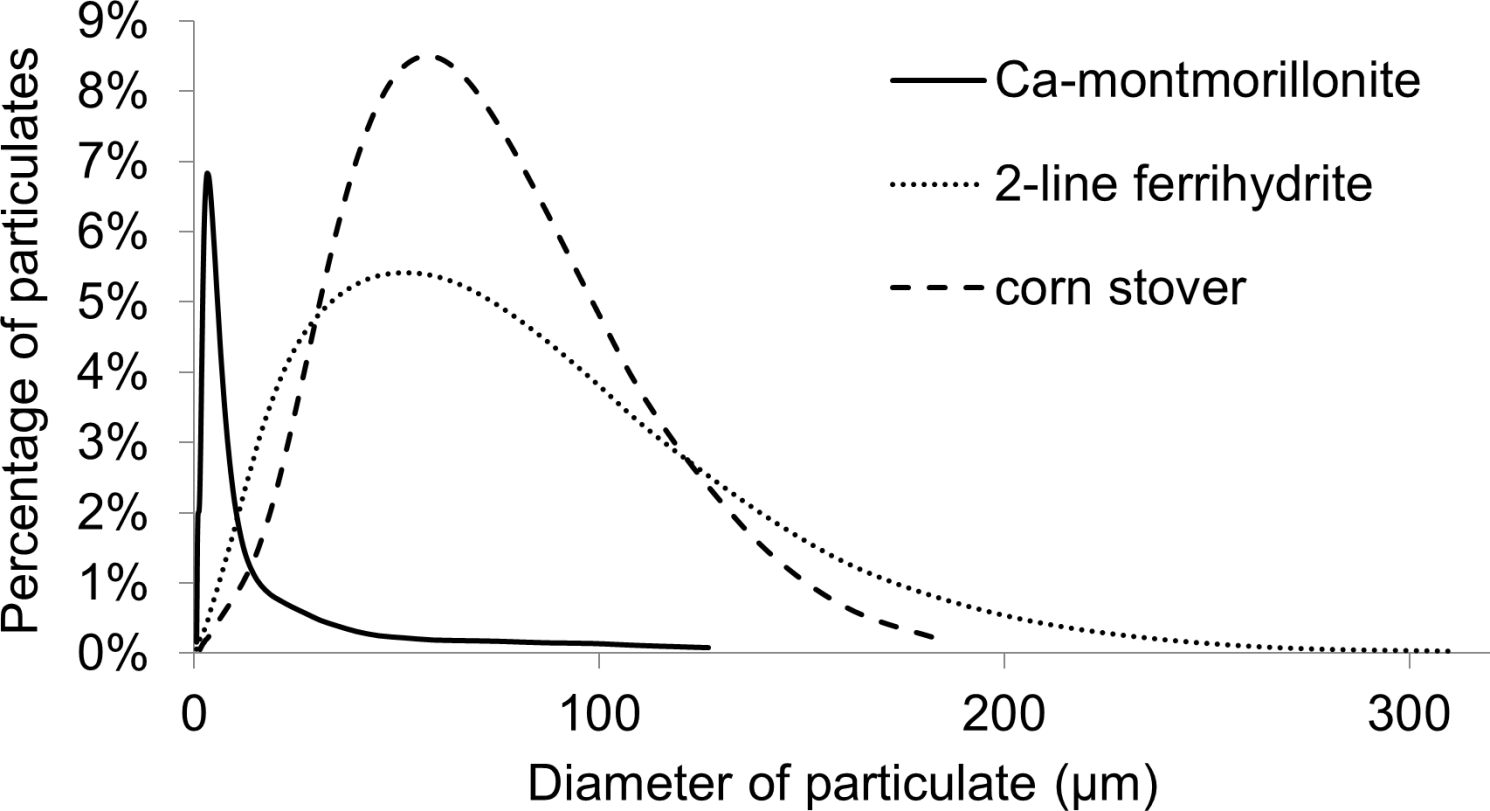
Particulate size distribution.

Measurements of carbon content, surface area, and size distribution for each of the particulates are listed in Table 1. Fig 3 shows a detailed size distribution for each of the particulates. Among the three particles, Ca-montmorillonite had the smallest size, while 2-line ferrihydrite and corn stover had similar size distributions. However, the surface areas of 2-line ferrihydrite and corn stover were different. The carbon content of corn stover was 38%, while the carbon content of Ca-montmorillonite was 0.055%, and the carbon content of 2-line ferrihydrite was zero.

**Table 1:** Measured particulate properties.

			**Size distribution (μm)**		
Particulate	Carbon content (%)	Surface area (m^2^/g)	D_x_^1^(10)	D_x_(50)	D_x_(90)
**2-line ferrihydrite**	0	142	9	41	118
**Ca-montmorillonite**	0.055	111	1	4	12
**corn stover**	38	3	18	53	106

^1^ D_x_ under size distribution indicates the maximum diameter of particulates (in μm) in 10, 50, and 90% of the entire population (e.g., 10 percent of the ferrihydrite particles have diameters < 9 μm)

**Fig 4:**
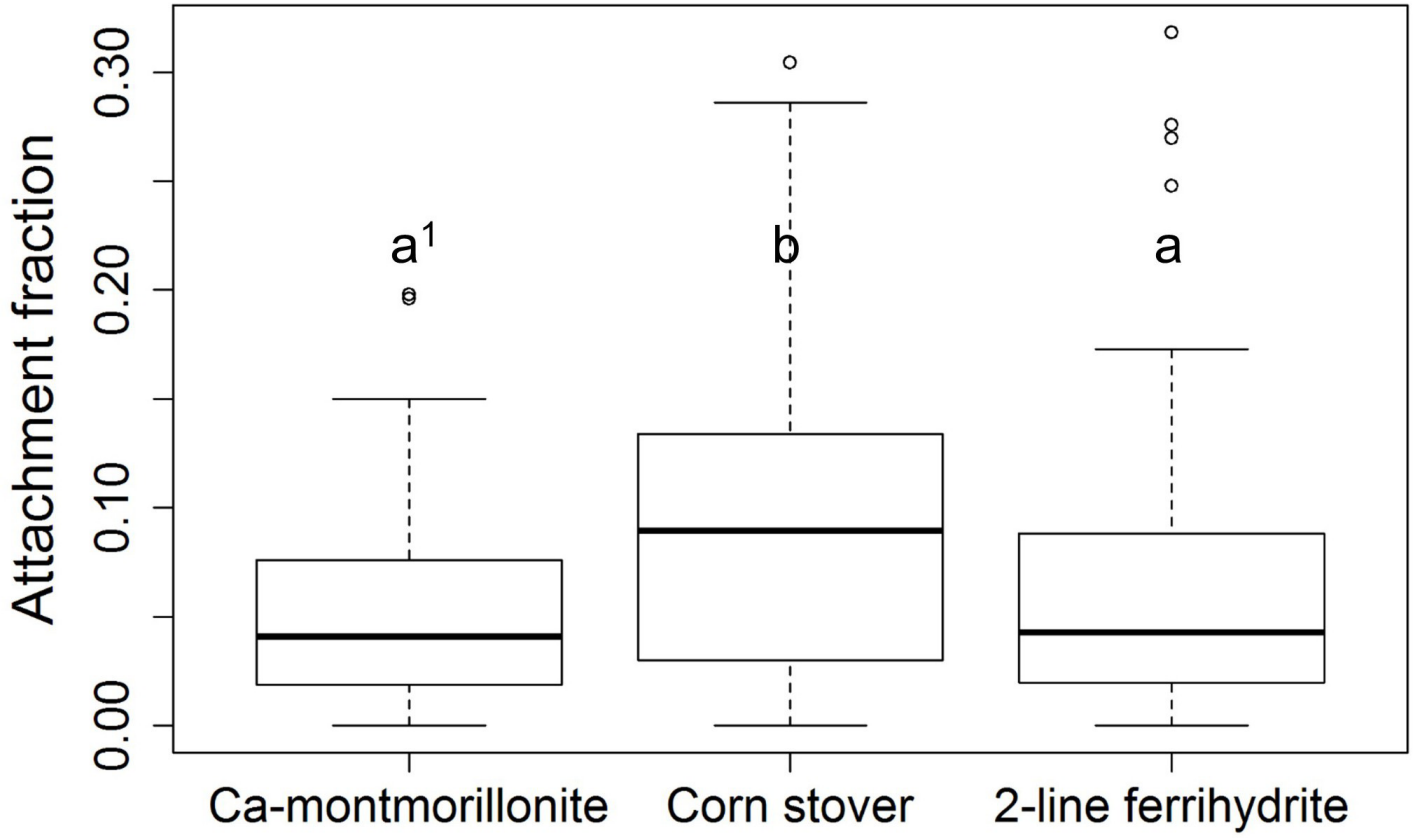
Boxplot^2^ of attachment fractions for each particulate type. 1. Values with the same letter are not different at α = 0.05, as determined by a Wilcoxon test. 2. Each plot shows five numerical values: the smallest observation (Q1 = Q2-1.5(Q4-Q1), the low end of the whisker), 25% quartile (Q2, low boundary of box), median (Q3, the band near the middle of box), 75% quartile (Q4, high boundary of box), and largest observation (Q5 = Q4+1.5(Q4-Q1), the high end of the whisker). Outliers, which are out of the range [Q1, Q5], are indicated by open circles.

For each *E*. *coli* strain, the attachment fraction to each particulate was obtained using the settling method. *E*. *coli—*particulate attachment fractions ranged from 0.00 to 0.32. Particulate type had an effect on *E*. *coli* attachment fractions (*p*-value = 0.0065). Fig 4 shows the boxplots of attachment fractions analyzed by each particulate type. The mean attachment fraction was 0.062 for 2-line ferrihydrite, 0.053 for Ca-montmorillonite, and 0.091 for corn stover. The attachment fractions to corn stover were significantly higher than those to both 2-line ferrihydrite (*p*-value = 0.0036) and Ca-montmorillonite (*p*-value = 0.022). The attachment fractions to 2-line ferrihydrite and Ca-montmorillonite were statistically similar (*p*-value = 0.90). These results are consistent with the particulate properties and suggest that, under typical environmental conditions, *E*. *coli* attachment can be affected by either surface area or the organic content of the particulate phase [43, 66].

### *E*. *coli* surface characteristics correlated with *E*. *coli*—Particulate attachment

We previously reported differences in *E*. *coli* characteristics in strains that were found in the stream sediment versus those in overlying water habitats [44]. Stream sediment *E*. *coli* isolates had significantly greater hydrophobicity, greater EPS protein content and EPS sugar content, less negative net charge, and higher point of zero charge when compared to *E*. *coli* isolates collected from the overlying stream water.

#### Correlations and model selection

**Fig 5:**
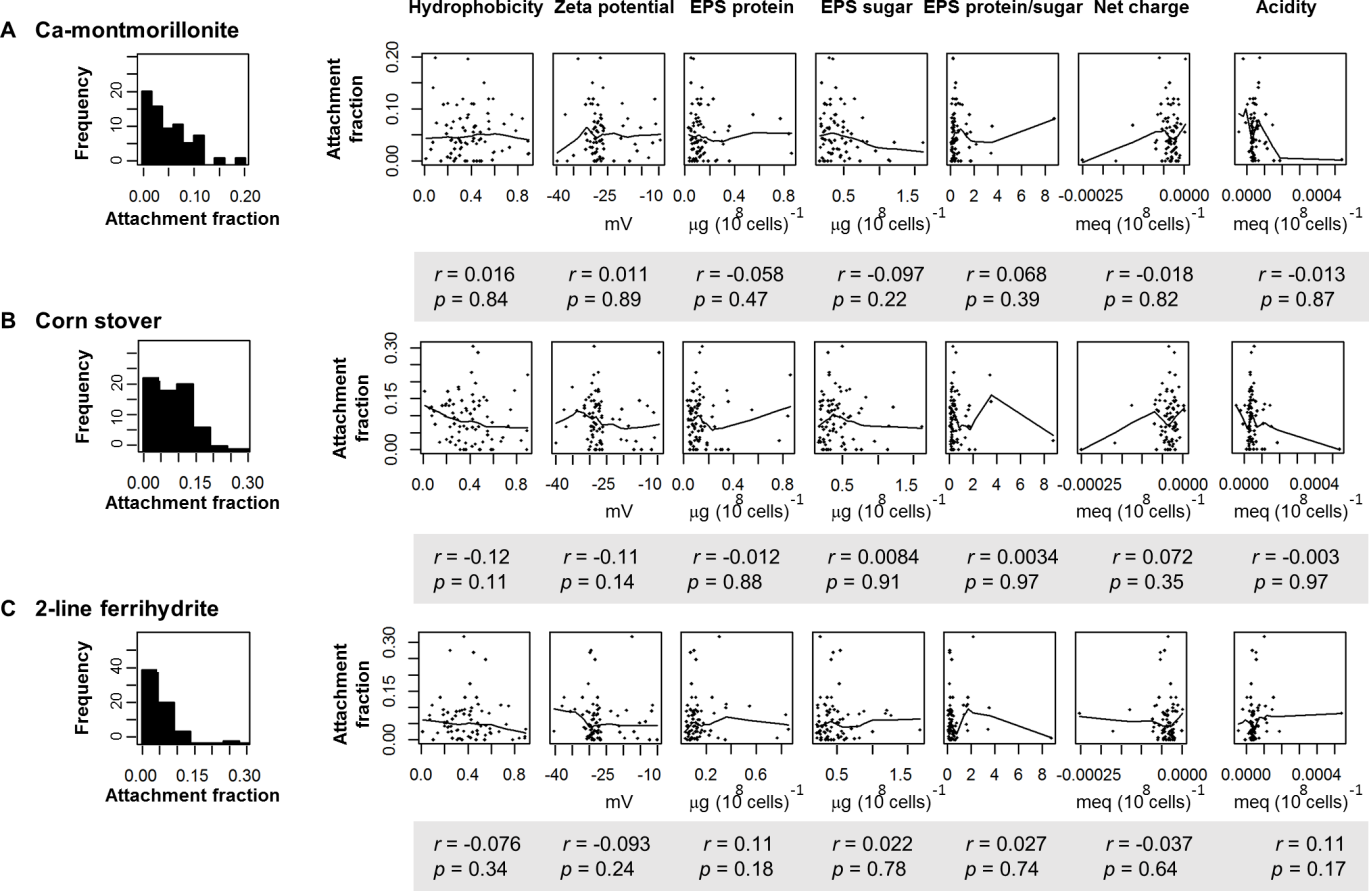
Attached fraction of each particulate type and correlation with each of the measured *E*. *coli* surface characteristics. Three rows of plots describe the results from three particulates: (A) Ca-montmorillonite, (B) corn stover, and (C) 2-line-ferrihydrite. The left panel shows the histogram of attachment fraction; The right panels contain the scatterplots of the attachment fraction vs. *E*. *coli* surface characteristic with the smoothing curve using the LOESS method. The correlation coefficient (r) with associated p-value (by the Kendall-tau) method is shown under each plot.

As indicated in Fig 5, for each particulate tested, the Kendall-tau correlation method found no significant linear relationship (*p*-value > 0.05) between the *E*. *coli* attachment fraction and any of the measured *E*. *coli* surface characteristics. Therefore, smoothing functions were applied to include the range of analyzed cell characteristics as variables in predicting the attachment fraction. A generalized additive model (GAM) was selected to explore the impact from multiple *E*. *coli* properties on attachment to particulates.

The histograms of cell characteristic results, including zeta potential, EPS protein, EPS polysaccharide, ratio of EPS protein to polysaccharide, absolute value of net charge, and acidity, indicated right-skewed distributions, as described previously [44]. The values of these characteristics were natural logarithm (ln) transformed for better normality prior to their use as predictor variables in the GAM model. Tensor product smooths were used to explore potential interactions between two predictor variables. The GAM models to fit *E*. *coli* attachment fractions to the three model particulates are discussed in detail below.

#### GAM model to predict attachment to Ca-montmorillonite

Natural log values of *E*. *coli* surface charge (ln |net charge|), of EPS sugar content (ln EPS sugar), and acidity (ln acidity) were shown to be useful to model the *E*. *coli* attachment fraction to Ca-montmorillonite. The interactions of ln acidity with ln net charge, and ln EPS sugar with ln acidity were also included. The model can be written as Eq 4:

$$\mathrm{Attachment}\;{\mathrm{fraction}}_{\mathrm{Ca-mont}}=\alpha_{0}+\alpha_{1}\times (\ln|\mathrm{net}\;\mathrm{charge}|)+f_{1}(\ln\;\mathrm{EPS}\;\mathrm{sugar})+f_{2}(\ln\;\mathrm{acidity})+f_{3}(\ln\;\mathrm{acidity},\ln|\mathrm{net}\;\mathrm{charge}|)+f_{4}(\ln\;\mathrm{acidity},\ln\;\mathrm{EPS}\;\mathrm{sugar})$$ (4)

**Fig 6:**
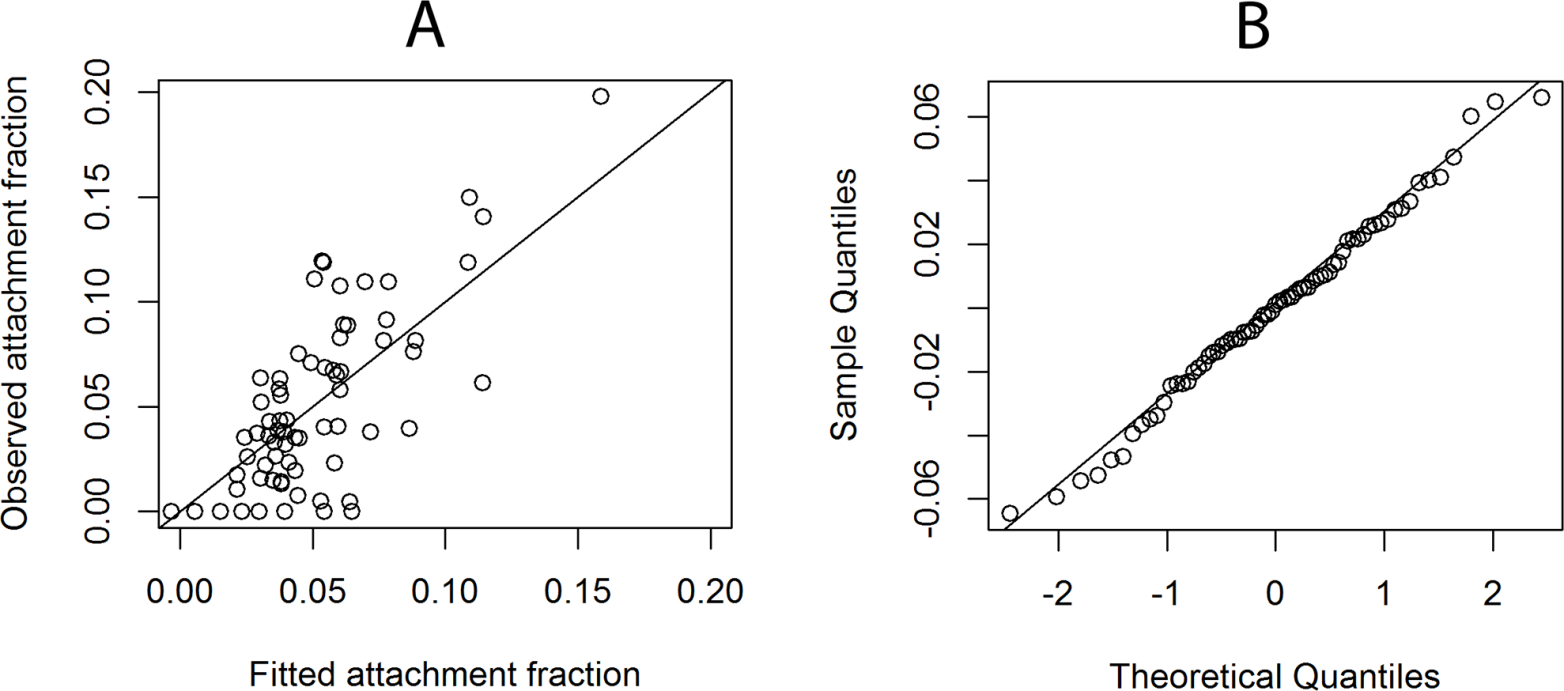
The performance of the model for predicting *E*. *coli* attachment to Ca-montmorillonite. (A) Plot of observed attachment fractions vs. fitted attachment fractions with straight line denoting observed = fitted. (B) Q-Q plot of residuals with straight line denoting a normal distribution.

**Fig 7:**
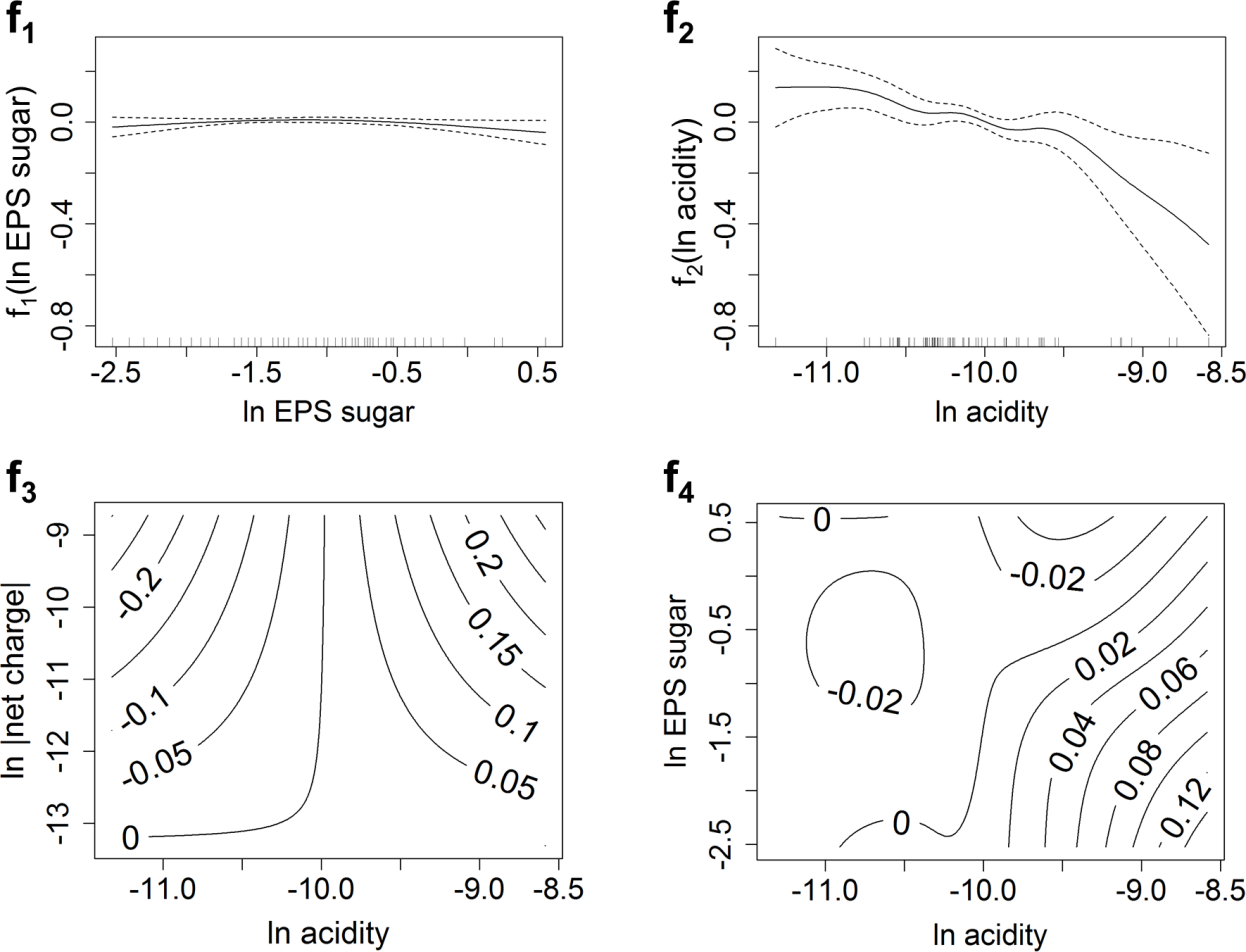
Plots of smoothed terms in predicting attachment to Ca-montmorillonite. Plot f_1_ shows the correlation between ln EPS sugar and the f_1_ term in Eq 4 with the 95% confidence interval (dotted curve). Similarly, plot f_2_ shows ln acidity. Plot f_3_ shows the estimate of the interaction term of ln |net charge| and ln acidity as contour lines, and plot f_4_ shows the estimate of the interaction term of ln EPS sugar and ln acidity.

The value of R^2^ for this model was 0.56, and model performance assessments are shown in Fig 6. The Q-Q plot of the residuals indicated a nearly normal distribution. Ln |net charge| showed a significant positive linear impact on the attachment fractions to Ca-montmorillonite (Table 2, S1 Fig). Since all *E*. *coli* strains measured had a net negative surface charge, we conclude that *E*. *coli* strains with a more negative net surface charge were more likely to attach to Ca-montmorillonite, possibly via Ca bridging of the bacterial cells with the negatively charged mineral surfaces. For smooth terms, estimated significance levels are listed in Table 3. While the impact of EPS sugar was not significant (*p*-value = 0.17), the interaction of EPS sugar and acidity did significantly impact the attachment fraction (*p*-value = 0.035). The contour plot of *f*_4_(ln acidity,ln EPS sugar) (Fig 7) indicated that the attachment would increase as ln acidity increases or as ln EPS sugar decreases. Surface acidity was included in three terms of the successful model; thus, the impact of surface acidity on attachment was complex. The plot of *f*_2_(ln acidity) shows that as ln acidity increased, the attachment to Ca-montmorillonite decreased significantly (*p*-value = 0.027). The contour plot of *f*_3_(ln acidity,ln |net charge|) presents the interaction between acidity and net charge. When ln acidity was less than -10.0, and as ln |net charge| increased, the attachment to Ca-montmorillonite decreased. When ln acidity was greater than -10.0, and as ln |net charge| increased, the attachment increased. When ln |net charge| was smaller than -13.0, the attachment to Ca-montmorillonite did not change as ln acidity changed. Finally, when ln |net charge| was greater than -13.0, the attachment increased as ln acidity increased.

**Table 2:** Estimated regression coefficients of ln |net charge| derived from GAM models to estimate attachment fractions.

Particulate	Estimate	SE	*p*-value	90% confidence interval	95% confidence interval
**Ca-montmorillonite**	0.025	0.010	0.013	(0.0091, 0.042)	(0.0059,0.045)
**corn stover**	-0.0064	0.0070	0.36	(-0.018, 0.0050)	(-0.20, 0.0073)

**Table 3:** Estimated significance of smoothed terms from GAM models to estimated attachment fractions.

Particulate	Term	*p*-value
**Ca-montmorillonite**	ln (EPS sugar)	0.16
	ln (acidity)	0.027
	tensor product (ln (EPS sugar), ln (acidity))	0.023
	tensor product (ln |net charge|, ln (acidity))	0.031
**corn stover**	Hydrophobicity	0.30
	ln (EPS protein)	0.40

#### GAM model to predict attachment to corn stover

Values of ln |net charge|, ln EPS sugar, and hydrophobicity were included in the model of *E*. *coli* attachment fractions to corn stover. The model can be written as Eq 5:

Attachmentfractioncornstover=α0+α1×(ln|netcharge|)+f1(lnEPSprotein)+f2(hydrophobicity) (5)

**Fig 8:**
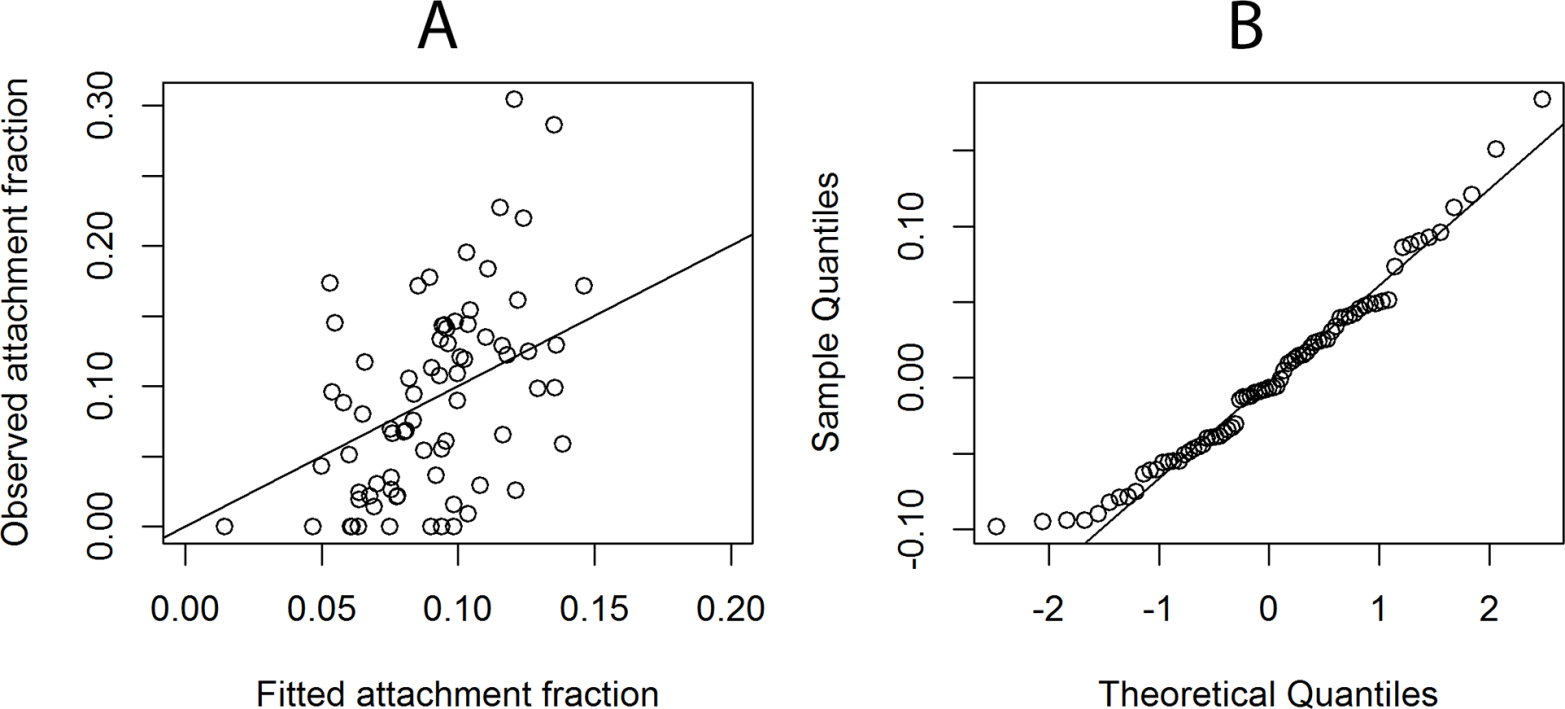
Plots for checking the performance of model for predicting *E*. *coli* attachment to corn stover. A. Plot of observed attachment fractions vs. fitted attachment fractions with straight line denoting observed = fitted. B. Q-Q plot of residuals with straight line denoting a normal distribution.

The R^2^ for this model is 0.23, and the model performance assessment is shown in Fig 8. Although the R^2^ is lower than the R^2^ for the Ca-montmorillonite GAM model, the overall model p-value is 0.090 and the distribution of the residuals still suggests this model is acceptable.

Ln |net charge| was included in the model as a linear term, but no significant impact on the attachment fractions to corn stover was observed (Table 2, S1 Fig). The 90% confidence intervals of ln |net charge| for Ca-montmorillonite and corn stover did not overlap, which indicated that the impacts from cell net surface charge on these two particles were significantly different from one another at α = 0.10. In addition, according to the model, the impacts from hydrophobicity and ln EPS sugar were neither linear nor significant (Table 3 and S2 Fig). Thus, the GAM model recognized the impacts of co-varying and interacting parameters, reinforcing the complexity and dynamic nature of attachment mechanisms.

#### GAM model to predict attachment to ferrihydrite

The attachment fraction to ferrihydrite ranged from 0 to 0.32, while the predicted values from the model ranged from 0.0086 to 0.12 (S3 Fig). For ferrihydrite, there was a non-normal distribution of residuals (S3 Fig). We interpret the failure in prediction to indicate that cell properties were less important to attachment to ferrihydrite than they were to the other particulates. Several aspects of the chemical environment may have played a role. For example, the pH of zero charge (PZC) of ferrihydrite from fresh preparations is typically reported to be ~8 [67], suggesting that at the pH of our system (~8) the net surface charge on ferrihydrite particles was close to zero. In addition, there was likely to be considerable competition from bicarbonate ions in the solution for any positively charged sites on the ferrihydrite surfaces. Thus, electrostatic or inner-sphere bonding reactions with bacterial EPS would not have promoted attachment. Since the ferrihydrite surfaces were quite polar and hydrophilic, it was not likely that hydrophobic interactions would have contributed to attachment.

## Discussion

To explore the impact of cell characteristics and particulate properties on attachment of *E*. *coli* bacteria, we conducted an attachment assay using 77 distinct *E*. *coli* strains and model particulates under laboratory conditions. The attachment fractions obtained from this study (0–32%) were lower than those of previous studies that considered mixed-strain environmental samples of *E*. *coli*. For example, Krometis et al. [7] found between 20 and 35% of *E*. *coli* were associated with settleable particles during normal flow conditions and 40% during storm flow conditions. Another study conducted by Schillinger and Gannon [3] determined that 10–20% of fecal coliform cells were adsorbed to suspended particles in untreated stormwater runoff. The differences indicate bacterial attachment of mixed environmental strains in surface waters might be different than our single strain and pure particulate assays suggest. In this study, we used a saturated CaCO_3_ solution to mimic surface water with pH 8 and ionic strength 10mM. While environmental water samples would have more complex ionic composition than our simulated systems [68], chemical interactions between particulate surfaces and colloidal bacterial cells are still likely to be regulated by pH and ionic strength [69].

Linear models are most often used to identify the impact of cell properties on cell attachment [13, 16, 21]. There are three potential problems with using a strict linear model with our results. First, linear methods consider only the correlation between attachment and one measured surface property. When assessing the impact of a single property on the attachment fraction, linear methods assume that all other properties are held constant. However, we have previously reported that two cell properties can be highly correlated; thus interaction terms are appropriate to explore [44]. Second, linear models assume that the values of the attachment fraction and each of the measured properties should be normally distributed. However, the distribution of attachment fractions and most cell properties are quite right skewed (Fig 5). Last, the correlation could be non-linear or polynomial. Here, we used the Kendall-tau correlation method (Fig 5) to explore the potential correlation between the attachment fraction and cell properties. The large diversity in cell characteristics, particulate properties, and attachment fractions among different *E*. *coli* strains and particulates clearly demonstrates the inadequacy of using a static parameter or a linear coefficient to assess the general attachment behavior of all *E*. *coli* strains. The modeling method employed in this study, GAM, can address many of the shortcomings of linear approaches. The GAM considered all measured cell properties and possible interactions simultaneously, and it can address not only linear but also non-linear correlations. Further, the normal distribution of the residuals (Fig 6B, Fig 8B) indicates the deviation model is reliable.

The uncertainty in estimating the cell count in the supernatant (C_s_) and the remaining suspension (C_r_) led to some variability in measuring the attachment fractions; the uncertainty was larger when the plate counts were less than 50 CFU. The uncertainty derived by cultivation of microorganisms can contribute to both laboratory bias (subsampling, dilution, pouring-plating) and measurement error [70, 71]. In this study, 11% of the observed attachment fractions were zero, and the models failed to predict the zero attachment fraction values, which contributed to decreased model performance. Therefore, minimum plate counts of 50 to 80 CFU per 45-mm petri dish are recommended to reduce the uncertainty when measuring *E*. *coli* attachment fractions.

The statistical approach to assessing *E*. *coli* attachment fractions is much different from current methods used in water quality models employed to predict bacteria fate and transport. For example, the Soil and Water Assessment Tool (SWAT), assumes linear regression between attached and planktonic bacteria, and the linear coefficient depends on the percentage of clay in sediment [11]. Bai and Lung [55] and Russo et al. [72] proposed attachment as a linear and reversible adsorption process, based on the work of previous researchers [73, 74]. Jamieson et al. [75] suggests that irreversible adsorption better represents attachment observed in low-ionic strength freshwaters. Regardless of the partitioning approach used, performance of models used to predict *E*. *coli* fate and transport at the watershed scale are lacking, and additional research and guidance is needed to better inform bacteria partitioning in models [76].

This work highlights the insufficiencies in commonly used methods to predict partitioning of environmental *E*. *coli*. More advanced statistical methods which consider variation in cell properties should be explored for better understanding of environmental microbial partitioning. Moreover, the mixed populations of microorganisms and particulates in environmental water samples might mean that the factors driving attachment could extend beyond the properties considered here. Such factors may include particle dispersion, temperature, discharge, and precipitation [77, 78]. Thus, the results from this work could be expanded in the future to explore more complex systems of diverse microorganisms and mixed particulates.

## Supporting information

S1 Fig Ln |net charge| showed a significant positive linear impact on the attachment fractions to Ca-montmorillonite (A); but an insignificant negative linear impact on the attachment fractions to corn stover (B). Dashed curves indicate 95% confidence interval bounds.
(TIF)

S2 Fig Plot of smoothed terms in predicting attachment to corn stover. Plot f1 shows the correlation between ln EPS protein and f1 term in Eq 5 with the 95% confidence interval in dotted curve. Similarly, f2 shows hydrophobicity.
(TIF)

S3 Fig The performance of the model for predicting *E*. *coli* attachment to 2-line ferrihydrite. (A) Plot of observed attachment fractions vs. fitted attachment fractions with straight line denoting observed = fitted. (B) Q-Q plot of residuals with straight line denoting a normal distribution.
(TIF)

S1 Dataset Data. (XLS)

S1 File *E*. *coli* surface properties differ between stream water and sediment environments. (PDF)

S2 File Allelic variation in outer membrane protein A and its influence on attachment of *Escherichia coli* to corn stover. (PDF)
