# Supplementary figures and images for: Escherichia coli attachment to model particulates: The effects of bacterial cell characteristics and particulate properties
Source: PLoS One. 2017 Sep 14;12(9):e0184664. doi: 10.1371/journal.pone.0184664 (PMC5599003; doi:10.1371/journal.pone.0184664)

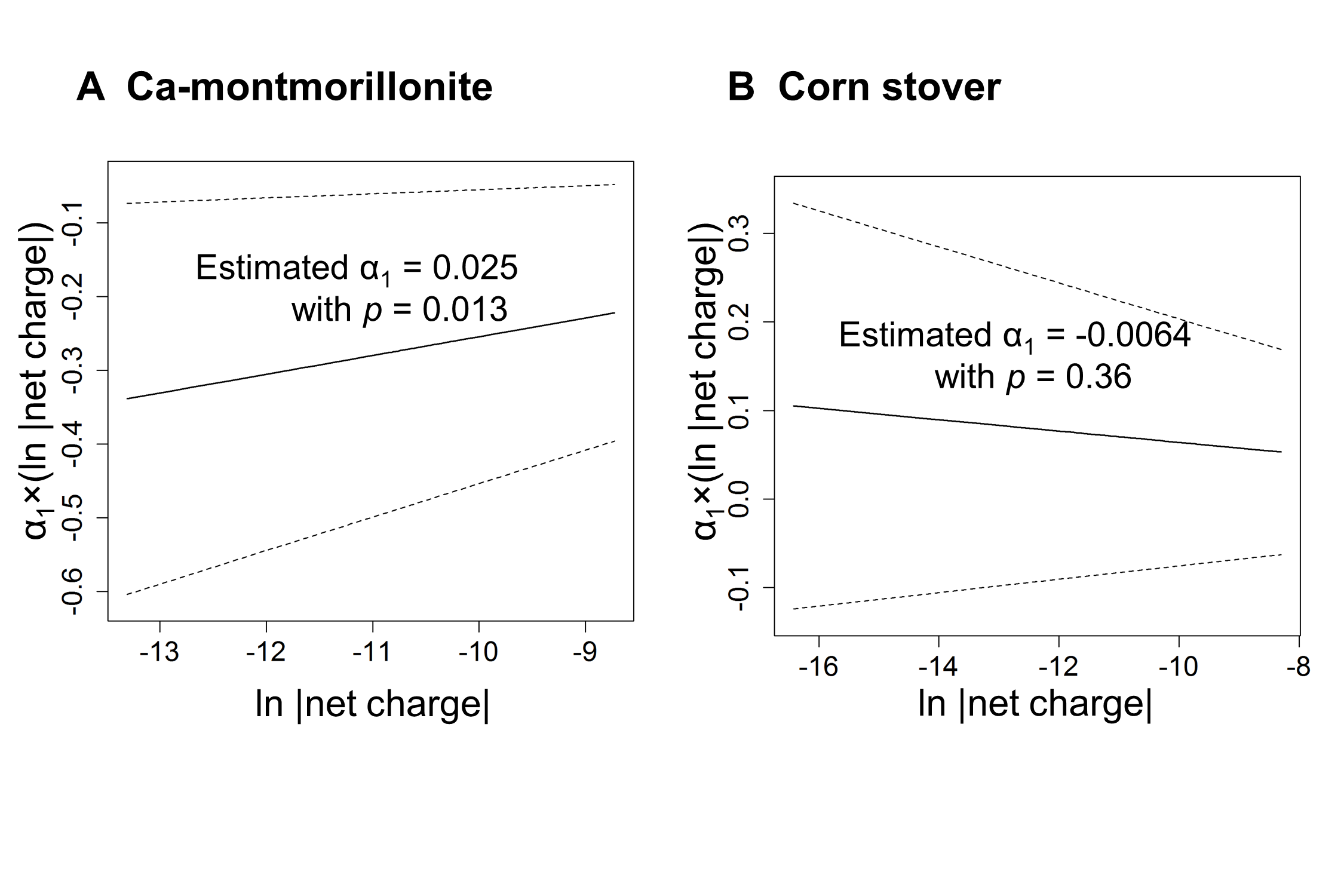

Supplement: S1 Fig — Ln |net charge| showed a significant positive linear impact on the attachment fractions to Ca-montmorillonite (A); but an insignificant negative linear impact on the attachment fractions to corn stover (B). Dashed curves indicate 95% confidence interval bounds. (TIF) [file pone.0184664.s001.tif]

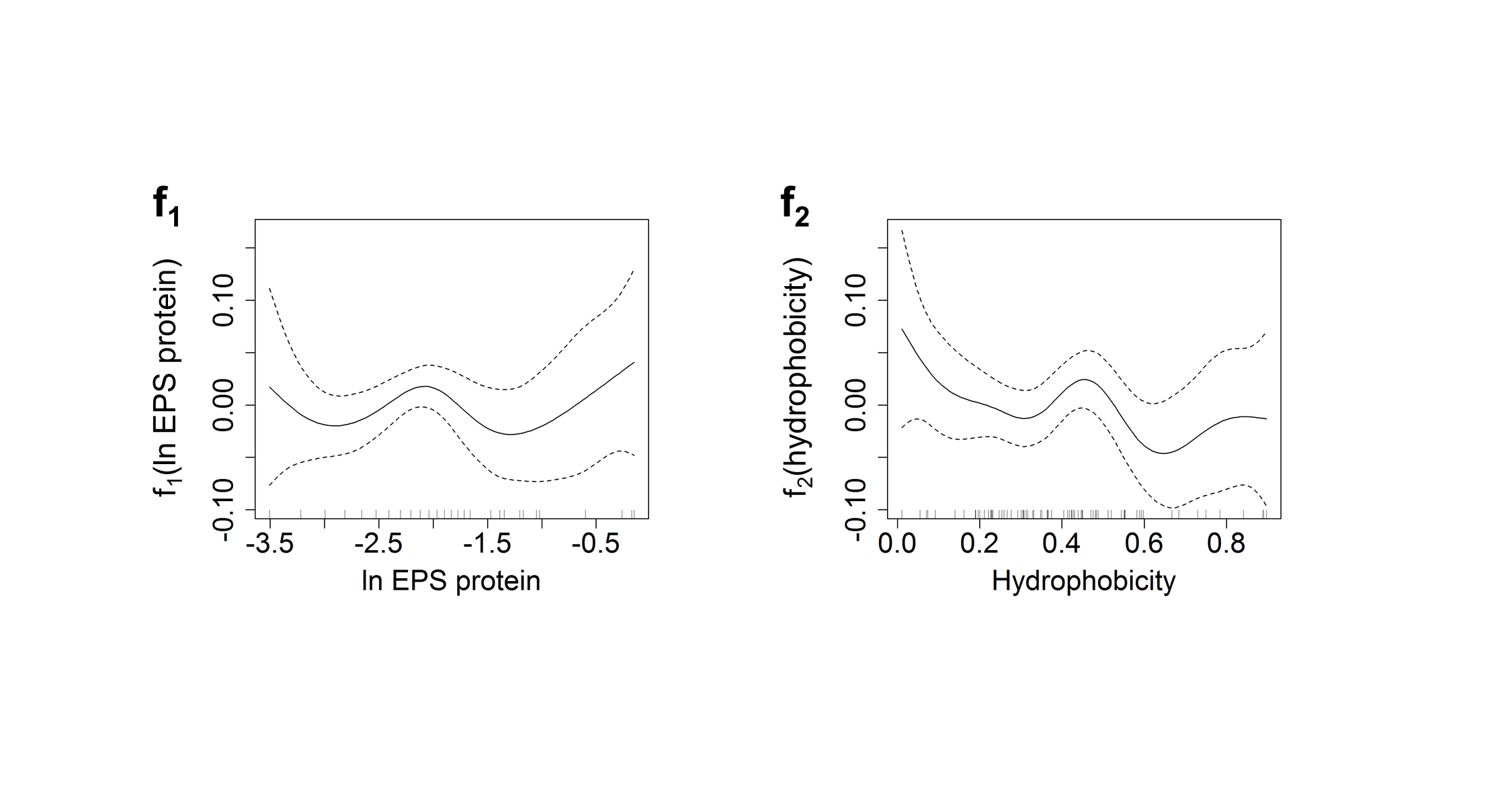

Supplement: S2 Fig — Plot f1 shows the correlation between ln EPS protein and f1 term in Eq 5 with the 95% confidence interval in dotted curve. Similarly, f2 shows hydrophobicity. (TIF) [file pone.0184664.s002.tif]

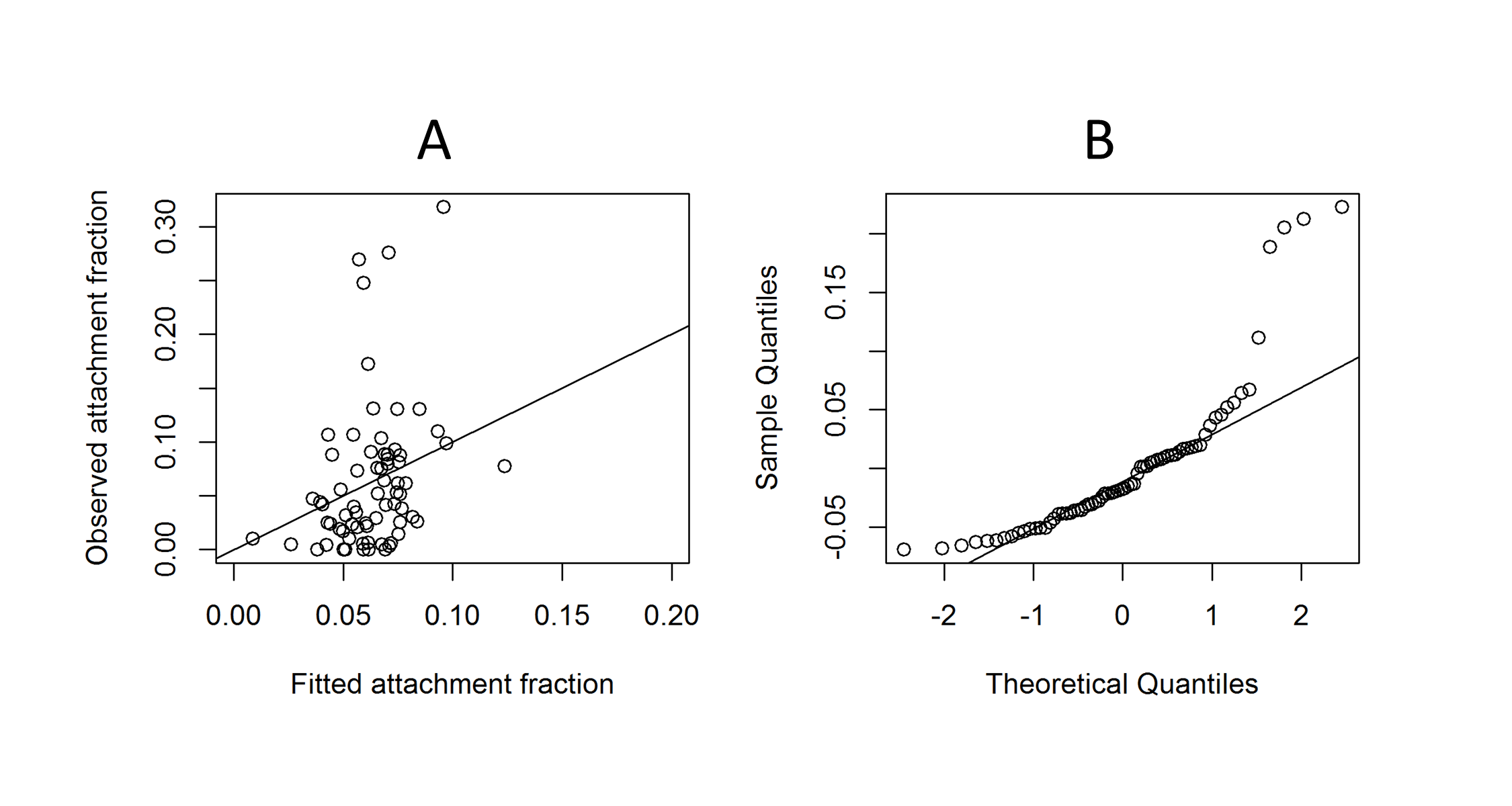

Supplement: S3 Fig — (A) Plot of observed attachment fractions vs. fitted attachment fractions with straight line denoting observed = fitted. (B) Q-Q plot of residuals with straight line denoting a normal distribution. (TIF) [file pone.0184664.s003.tif]
