# Supplementary material for: Escherichia coli attachment to model particulates: The effects of bacterial cell characteristics and particulate properties
Source: PLoS One. 2017 Sep 14;12(9):e0184664. doi: 10.1371/journal.pone.0184664 (PMC5599003; doi:10.1371/journal.pone.0184664)
Supplement: S1 File — (PDF) [file pone.0184664.s005.pdf]

frontiers in Microbiology

ORIGINAL RESEARCH
published: 01 November 2016
doi: 10.3389/fmicb.2016.01732

E. coli Surface Properties Differ between Stream Water and Sediment Environments

Xiao Liang[1], *Chunyu Liao*[2], *Michael L. Thompson*[3], *Michelle L. Soupir*[1]*, *Laura R. Jarboe*[2,4] *and Philip M. Dixon*[5]

[1] Department of Agricultural and Biosystems Engineering, Iowa State University, Ames, IA, USA, [2] Department of Microbiology, Iowa State University, Ames, IA, USA, [3] Department of Agronomy, Iowa State University, Ames, IA, USA, [4] Department of Chemical and Biological Engineering, Iowa State University, Ames, IA, USA, [5] Department of Statistics, Iowa State University, Ames, IA, USA

The importance of *E. coli* as an indicator organism in fresh water has led to numerous studies focusing on cell properties and transport behavior. However, previous studies have been unable to assess if differences in *E. coli* cell surface properties and genomic variation are associated with different environmental habitats. In this study, we investigated the variation in characteristics of *E. coli* obtained from stream water and stream bottom sediments. Cell properties were measured for 77 genomically different *E. coli* strains (44 strains isolated from sediments and 33 strains isolated from water) under common stream conditions in the Upper Midwestern United States: pH 8.0, ionic strength 10 mM and 22°C. Measured cell properties include hydrophobicity, zeta potential, net charge, total acidity, and extracellular polymeric substance (EPS) composition. Our results indicate that stream sediment *E. coli* had significantly greater hydrophobicity, greater EPS protein content and EPS sugar content, less negative net charge, and higher point of zero charge than stream water *E. coli*. A significant positive correlation was observed between hydrophobicity and EPS protein for stream sediment *E. coli* but not for stream water *E. coli*. Additionally, *E. coli* surviving in the same habitat tended to have significantly larger $(GTG)_5$ genome similarity. After accounting for the intrinsic impact from the genome, environmental habitat was determined to be a factor influencing some cell surface properties, such as hydrophobicity. The diversity of cell properties and its resulting impact on particle interactions should be considered for environmental fate and transport modeling of aquatic indicator organisms such as *E. coli*.

Keywords: ***E. coli*, water quality, stream, surface property, particle**

OPEN ACCESS

Edited by:
Martin G. Klotz,
City University of New York, USA

Reviewed by:
Greg D. O'Mullan,
City University of New York, USA
Stephanus Nicolaas Venter,
University of Pretoria, South Africa

Correspondence:
Michelle L. Soupir
msoupir@iastate.edu

Specialty section:
This article was submitted to Aquatic Microbiology, a section of the journal Frontiers in Microbiology

Received: *22 June 2016*
Accepted: *17 October 2016*
Published: *01 November 2016*

Citation:
Liang X, Liao C, Thompson ML, Soupir ML, Jarboe LR and Dixon PM (2016) E. coli Surface Properties Differ between Stream Water and Sediment Environments. Front. Microbiol. 7:1732. doi: 10.3389/fmicb.2016.01732

INTRODUCTION

Currently, pathogens are the leading cause of water quality impairments in rivers and streams in the United States, as often indicated by elevated levels of *E. coli* (USEPA, 2014). Therefore, improved understanding the variations of *E. coli* properties is needed for predicting fate and transport of the bacteria and to support the development of plans to reduce bacterial contamination of waters. Recent studies have indicated that there is high diversity of *E. coli* isolates in the environment

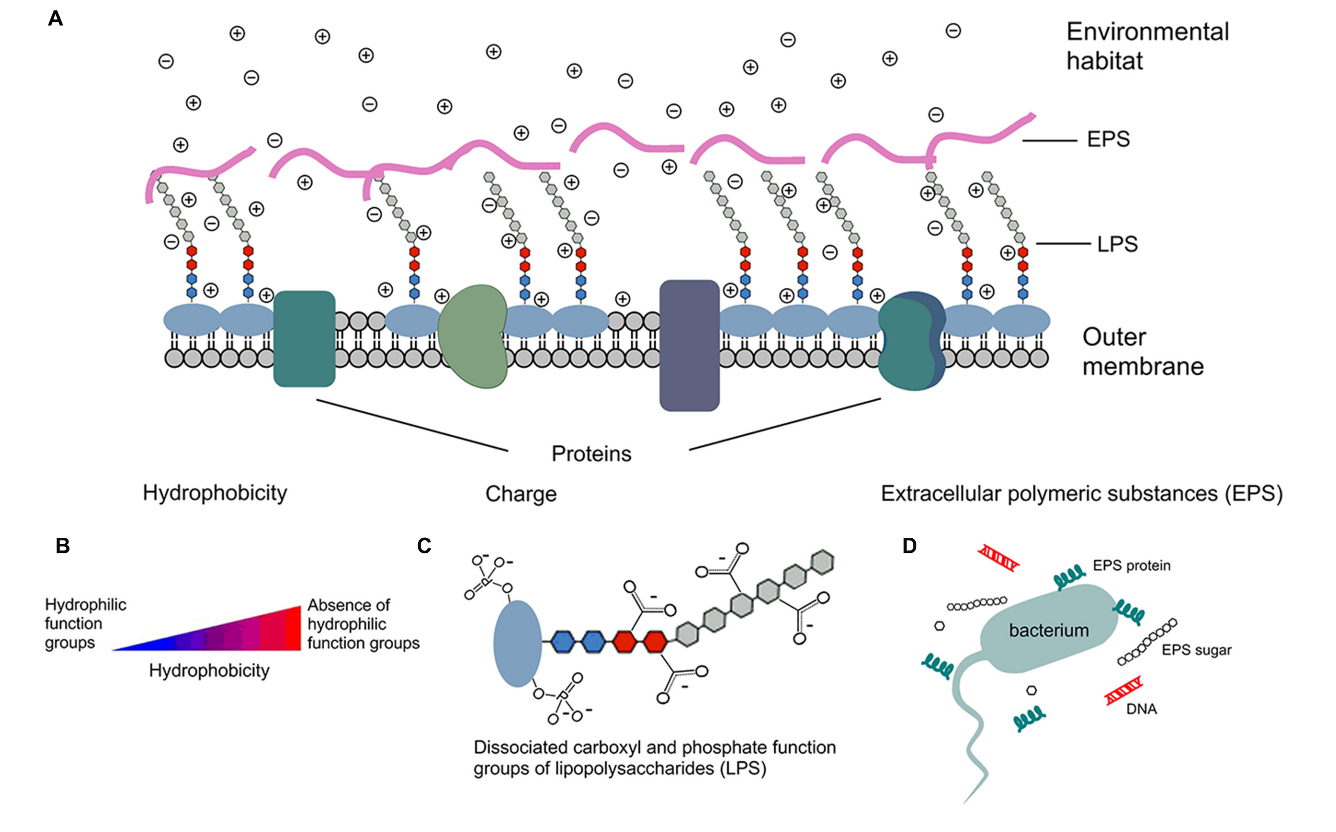

FIGURE 1 | Schematic depiction of *E. coli* surface properties. ⊕ cation in solution; ⊖ anion in solution; – negative charge due to dissociation. **(A)** *E. coli* outer membrane has several components that contribute to cell surface properties, such as hydrophobicity, surface charge, zeta potential, and components of extracellular polymeric substance (EPS). **(B)** Cells that are absence of hydrophilic function groups are more hydrophobic than cells with hydrophilic function groups. **(C)** Surface is negatively charged due to the dissociation of carboxylic acid and phosphate functional groups; similar dissociation can also occur on the EPS and phospholipids of the membrane. Therefore, more cations than anions exist in solution, and that separation of charge is measured by the zeta potential. **(D)** EPS are mainly composed of sugars and proteins, but they may also include other macromolecules such as DNA.

(Lu et al., 2005; Bolster et al., 2009; Cook et al., 2011). This strain-level diversity has been described by differences in both genotype and phenotype, and therefore it likely impacts the fate and transport of *E. coli*. Moreover, bacterial survival and growth is a dynamic process affected by bacterial surface properties, such as extracellular polymeric substance (EPS), hydrophobicity, and net charge; both genomic and environmental factors regulate those properties (Goulter et al., 2009).

Under typical stream pH in the Upper Midwestern United States (Dickson and Koohmaraie, 1989; Fein et al., 2005; Warnes et al., 2012), *E. coli* surfaces are negatively charged due to the dissociation of carboxyl and phosphate groups in the peptidoglycan and lipopolysaccharides of cell walls (Goulter et al., 2009; Warnes et al., 2012), as shown by **Figure 1**. While the magnitude of the surface charge of bacteria is highly environment-dependent (Fein et al., 2005), it can impact the bacterial state by repulsion of similarly charged particulates and by attraction of oppositely charged particulates (Dickson and Koohmaraie, 1989; Bolster et al., 2009). The hydrophobicity of a bacterial cell is determined by functional groups of both residues and structures on the surface of the cell, which can be either hydrophilic or hydrophobic (Vandermei et al., 1991). Hydrophobicity may change according to growth phase and growth condition, while the carbon content of the growth medium could positively impact hydrophobicity. Such impacts are partially due to the effects on lipid composition (Zikmanis et al., 2007). The presence of divalent cations, such as Ca^{2+} and Mg^{2+}, could increase bacterial hydrophobicity (Khemakhem et al., 2005), since the cations principally attach to proteins on the bacterial surface and decrease their hydrophilicity (Jorand et al., 1998; Hoa et al., 2003; Wilen et al., 2003).

Extracellular polymeric substance are high-molecular-mass compounds secreted by microorganisms at the outer cell surface (Liao et al., 2015). They are mainly composed of polysaccharides and proteins, but they may also include other macromolecules such as DNA, lipids, and humic-like substances. EPS contribute to the overall heterogeneity of the bacterial surface (Walker et al., 2005; Zhao et al., 2014) and play an important role in cell aggregation, cell adhesion, and protection of cells from hostile environments (Dogsa et al., 2005; Vu et al., 2009; Bruckner et al., 2011). For example, the formation of biofilms in stream bottom sediments requires involvement of EPS (Sheng et al., 2010). The sugar/protein

ratio of EPS has been positively correlated with the cell surface charge (Shin et al., 2001). Bolster et al. (2009) reported that EPS production mostly occurred in the late growth phase of bacteria. Moreover, EPS structure has been found to become more compact as environmental pH decreases (Dogsa et al., 2005).

Current water quality assessment techniques are based on environmental sampling, for which only the suspended populations of fecal indicator bacteria are collected (Bai and Lung, 2005; Pandey and Soupir, 2013); this procedure does not assess microbial contamination of stream bottom sediments. However, previous research has indicated that after entering surface waters, microorganisms often partition into the planktonic state or they attach to suspended soil and organic particles (Jeng et al., 2005; Hipsey et al., 2006; Pachepsky et al., 2008; Liang et al., 2014). The populations of bacteria surviving in bottom sediments are protected from ultraviolet radiation (Bitton et al., 1972; Schillinger and Gannon, 1985), resulting in an extended survival time. When stream bottom sediments are disturbed during changes in flow, there is increasing likelihood of resuspension back into the water column (Song et al., 1994; Davies and Bavor, 2000; Jamieson et al., 2005). Therefore, improved understanding of the properties of sediment-associated *E. coli* is critically important for understanding bacterial fate in the environment.

An assessment of the variation of *E. coli* cell properties in different environments (stream bottom sediments versus the overlying water column) is needed to better understand environmental fate and transport. In this study, we divided the potential impacts on bacterial surface properties into two parts: genomic impact (intrinsic) and environmental impact (extrinsic). The overall goal was to determine if differences in *E. coli* cell surface properties were due primarily to extrinsic or intrinsic properties or to an interaction of the two. We assessed 77 genomically distinct *E. coli* strains with respect to hydrophobicity, zeta potential, net charge, total acidity, and EPS composition (protein and sugar). The objectives of our study were: (1) to compare bacterial properties between *E. coli* isolated from two environmental habitats, stream sediments, and stream water; (2) to determine the correlations among bacterial surface properties within each environmental habitat and compare the correlations obtained from different environmental habitat; (3) to explore the relationship between genomic similarity and environmental habitat; and (4) to investigate bacterial surface properties as a function of environmental impact (extrinsic), regardless of genomic similarity (intrinsic).

MATERIALS AND METHODS

To investigate the potential impacts from intrinsic genomic and extrinsic environmental aspects, *E. coli* strains collected from two environmental habitats were studied. For each *E. coli* strain, several bacterial surface properties were measured: hydrophobicity, zeta potential, net charge, total acidity, and EPS composition by extraction and colorimetric techniques. Genome similarities were also analyzed for each pair of *E. coli* strains.

E. coli Sampling and Analysis

Stream sediment and water were collected six times from two locations along Squaw Creek in Ames, IA, USA, in 2012 and 2013: Cameron School Road (latitude 42.0707, longitude −93.6728), and Brookside Park (latitude 42.0290, longitude −93.6288). Water samples were collected by lowering a horizontal polycarbonate water bottle sampler (2.2 L, Forestry Suppliers Inc., Jackson, MS, USA) from a bridge into the center of the creek at both of the locations. Sediment samples were collected from the top 2–3 cm of the streambed using a shallow water bottom dredge sampler (15 cm × 15 cm opening, Forestry Suppliers Inc., Jackson, MS, USA) at the same location as the water samples were collected. Immediately after collection, samples were placed on ice. The sediment-associated *E. coli* were detached by stirring a mixture of sediment and deionized water (ratio 1:1) for 15 min at approximately 200 rpm using a magnetic stir bar under room temperature (Pandey et al., 2012). One milliliter of the resulting sediment solution was filtered through a 0.45-μm cellulose filter paper (EMD Millipore; Pittsburg, PA, USA). *E. coli* strains were incubated on the filter paper using modified mTEC agar plates (USEPA, 2002). A single colony was selected from each agar plate and the plate-streaking method was applied to ensure that the selected colony was formed by only one *E. coli* strain. Two hundred strains were isolated from the stream sediment. Each 100-mL water sample was filtered through a 0.45-μm filter paper, and another 200 strains were obtained from the water samples. After isolation, frozen stocks were made according to standard technique. The strains were inoculated in Luria–Bertani liquid media (BD Biosciences; San Jose, CA, USA), grown to the stationary phase, and stored at −80°C in 15% glycerol.

Computer-Assisted Rep-PCR DNA Fingerprint Analysis

Rep-PCR was performed (Rademaker and de Bruijn, 1997) with $(GTG)_5$ (5′-GTGGTGGTGGTGGTG-3′) as primer (Mohapatra and Mazumder, 2008; Mohapatra et al., 2008; Ma et al., 2011). The PCR reaction contained 12.5 μL PCR-master-mix (2X, Qiagen), 10 μL primer (50 pmol), and 2.5 μL water. A small fraction of a fresh *E. coli* colony was transferred to the PCR mixture as the template by using a 1-μL loop. PCR was conducted in a C1000 Thermal Cycler (Bio-Rad, Hercules, CA, USA). The thermo cycler program was set for an initial denaturation (95°C for 2 min), 32 cycles of denaturation (94°C for 3 s and 92°C for 30 s), annealing (40°C for 1 min), extension (65°C for 8 min), and a final extension (65 °C for 8 min). Then 10 μL of resulting PCR products and 2 μL of 6X loading dye mixture (Life Technology, Grand Island, NY, USA) were loaded onto 1.5% agarose gel, and the 1 Kb Plus DNA ladder (Life Technology, Grand Island, NY, USA) was loaded into every tenth well and was used as an external control for normalization. Electrophoresis was applied at 4°C and 80 V for 13.5 h, and the sample was stained for 20 min in TAE solution containing 0.5 μg mL^{-1} ethidium bromide. Gel pictures were captured with a Molecular Imager ChemiDoc (Bio-Rad, Hercules, CA, USA).

The resulting gel image files were imported into Bionumerics (version 7.1, Applied Maths, Kortrijk, Belgium) for

normalization, band identification, and cluster analysis. Bands of more than 5000 bp and less than 300 bp were eliminated from analysis to avoid false clustering. Similarity coefficients for each strain pair were generated by Pearson's correlation method with a band matching tolerance of 0.5%, and an optimization value of 0.5% (BioNumerics, 2013). Unweighted pair groups with mathematical averages (UPGMA) was used for clustering and generating the dendrogram. Strains with similarity less than 90% were considered genomically different. *E. coli*- $(GTG)_5$ genomic similarity matrix was also obtained from Bionumerics.

Genomically different *E. coli* strains were assigned to one of seven phylotypes (A, B1, B2, A/C, D/E, E, or F) using the revised Clermont method (Clermont et al., 2013). Strains were inoculated in M9 broth (minimal media) with 0.4% (w/w) glucose at 37°C and incubated to early stationary phase (OD600 = 1.0–1.5). To harvest the cells, *E. coli* were centrifuged for 15 min at 4,000 rpm/1878 × *g* (Centrifuge 5430R with Rotor F-35-6-30, Eppendorf, Hauppauge, NY, USA) at 4°C. The supernatant was discarded, and the cell pellet was used for property analysis.

Cell Properties

The microbial adhesion to hydrocarbon (MATH) method was employed to estimate the hydrophobicity of the *E. coli* strains (Rosenberg et al., 1980; Pembrey et al., 1999). Briefly, the cell pellet was resuspended in 4 mL of deionized water and OD546 of the cell suspension (initial OD546) was measured by spectrophotometer (HACH, Loveland, CO, USA). Then the cell suspension was transferred to individual glass test tubes (1.7 cm in diameter, 15 cm in length), each of which contained 1 mL of dodecane (99%, Fisher Scientific, Fair Lawn, NJ, USA). The tubes were vortexed (Fisher Scientific, Fair Lawn, NJ, USA) at full speed for 2 min and then left vertically at room temperature for 15 min for phase separation. The OD546 of the aqueous phase was determined and hydrophobic partitioning of the bacterial suspension was calculated by using this equation from Pembrey et al. (1999): hydrophobic partitioning = (initial OD546 − OD546 of aqueous phase)/initial OD546. The analysis was performed in triplicate.

Zeta potential measurements were performed at room temperature using a Zetasizer Nano-ZS. To mimic typical stream environments of the Upper Midwestern United States, a solution of $CaCO_3$ was prepared by diluting saturated $CaCO_3$ solution to pH 8.0 and an ionic strength of 10 mmol L^{-1}. The *E. coli* cell pellet was washed twice with $CaCO_3$ solution then suspended in $CaCO_3$ solution to OD600 = 0.1. The resulting suspension was poured into a disposable capillary cell (DTS1070). The average and standard deviation of 12 runs were recorded.

Potentiometric titration of *E. coli* cells was conducted to measure the acidity of the bacterial surface. The harvested cell pellet was suspended in $CaCO_3$ solution (pH = 8.0, ionic strength of 10 mmol L^{-1}). The concentration of *E. coli* cells in the suspension was then determined by cellometer (Auto M10, Nexcelom Bioscience LLC, Lawrence, MA, USA). Then the solution pH was adjusted to 4.0 by addition of 0.01 mol L^{-1} HCl. Next, the *E. coli* suspension was purged with nitrogen gas for 1 h to remove dissolved carbon dioxide (Walker et al., 2005), and then it was titrated with NaOH (0.01 mol L^{-1}) from pH 4.0 to 10.0 using a titrator (Multitasking titration system, Lab synergy, Goshen, NY, USA). A blank titration with $CaCO_3$ solution without *E. coli* was run separately. The number of moles of deprotonated sites was calculated as described by Fein et al. (2005):

$$[NC]_{\text{Net charge meq per } 10^8 \text{ cells}} = \left[\frac{\begin{array}{c}(C_A - C_B - [H^+] - [OH^-])_{\text{sample}} \\ -\,(C_A - C_B - [H^+] + [OH^-])_{\text{blank}}\end{array}}{N_{\text{bact}}} \right] \times 10^{-3}$$

where N_{bact} is the total number of cells per mL of solution obtained by the cellometer; C_A and C_B are the concentrations (in mmol L^{-1}) of acid and base (including initial amounts of acid or base added to the suspension prior to the titration); $[H^+]$ and $[OH^-]$ are the concentrations of H^+ and OH^-, calculated from themeasured pH. The net charge was determined as the difference of charge between the *E. coli* suspension sample and the blank. The total acidity was obtained by subtracting the net charge at pH 10.0 from the net charge at pH 4.0. The surface charge at pH 8.0 and the point of zero charge (PZC) were also points of interest. The sample analyses were performed in duplicate while the blank solutions were titrated in triplicate and averaged. **Supplementary Figure A1** shows an example of the potentiometric titration curve to demonstrate the useful information which can be obtained from this measurement.

The EPS, specifically the total protein and the polysaccharide content, was determined by an extraction method (Chang, 2005). Briefly, *E. coli* cells were incubated on a 0.45-μm filter membrane on multiple mTEC agars overnight at 37°C to obtain the total amount of *E. coli* cell within the range of 3×10^{10} to 6×10^{10} cells, and then the membrane was placed in 30 mL of 0.85% (w/v) NaCl solution. The *E. coli* concentration was measured by cellometer. After centrifugation at 16,300 × *g* for 30 min at 4°C, the supernatant was filtered through a 0.45-μm filter. The filtrate was then added to 90 mL of ice-cold 100% ethanol and stored at −20°C for 24 h. Finally, the EPS pellet was harvested by centrifugation at 16,300 × *g* for 30 min at 4°C and air-dried in a fume hood. The analysis of EPS protein was conducted using the Lowry method (Lowry et al., 1951), which is a spectrometric method based on measurement at a wavelength of 500 nm using bovine serum albumin (Sigma-Aldrich, St. Louis, MO, USA) as the standard. The EPS sugar was analyzed by the phenol-sulfuric acid method, which is based on measurement at a wavelength of 488 nm using xanthan gum as the standard (Dubois et al., 1956; Im et al., 2010).

Data Analyses

Statistical analysis of data was performed using R project software (version 3.1.3, Institute from Statistics and Mathematics, Vienna University of Economics and Business, Vienna, Austria). The non-parametric Wilcoxon signed-rank test was used to determine

if any of the properties varied between sediment *E. coli* strains and water *E. coli* strains. To investigate the correlation between any two *E. coli* properties, the Kendall-tau correlation method and the LOESS-smoothing method were applied. The Mantel test was conducted to determine the correlation between *E. coli*-(GTG)$_5$ genomic similarity and environmental habitat (stream bottom sediments or overlying water). Moreover, phylogenetic generalized least squares was employed to explore the impact of environmental habitat on bacterial properties, by excluding the potential impact from genomic similarity. Phylogenetic methods are used in the analysis of interspecies data because species are non-independent for statistical analysis (Revell, 2010). When applying the phylogenetic generalized least squares analysis (R package 'pGLS'), one bacterial property was considered as the response (***Y***), environmental habitat was considered as the binary predictor (***X***), and similarity matrix was considered as the variance-covariance matrix. The method uses a variance-covariance matrix to weight the predictors (Mao, 2015).

RESULTS AND DISCUSSION

E. coli Strain Selection, Dendrogram, and Phylo-Type

By computer-assisted rep-PCR DNA fingerprint analysis, 45 sediment strains (22.5% of 200 strains) and 33 water strains (16.5% of 200 strains) were considered genomically distinct on the basis of the 90% similarity criterion. Phylo-type analysis was conducted to query the origin of the isolates and to put the work into the context of other recently published environmental studies. **Figure 2** shows the dendrogram of these 78 selected strains based on the genome similarity score from the rep-PCR fingerprint as well as the phylo-type, while **Supplementary Figure A2** shows the dendrogram of the electrophoresis image. There was no obvious cluster pattern for *E. coli*-(GTG)$_5$ genomic profiles of strains from the same environmental habitat (stream water or sediment). Phylo-typing revealed that sediment isolates had more diverse phylo-type (25% classified as D/E, 23% as B1, 20% as B2, 16% as A/C, 11% as A, 2% as E, and 2% as F) when compared to water isolates in which 56% of isolates were classified as B1 (additionally, 19% classified as D/E, 9% classified as A, 9% classified as A/C, 3% as E, and 3% as F). Previously *E. coli* from phylogenetic groups A and B1 were associated with livestock (Cortes et al., 2010; Schulz et al., 2016) while B2 and D strains are more likely to be associated with extraintestinal infections. Note that one sediment strain (strain No. 122) had insufficient growth in M9 broth to proceed, so further analyses were based on 44 sediment strains and 33 water strains. This analysis demonstrates that each of the isolates characterized here was genomically distinct from the others, and variation in phylo-types existed.

E. coli Property Comparison between Two Environmental Habitats

Although each *E. coli* strain was subjected to the same storage and growth conditions, diversities in properties of *E. coli* derived

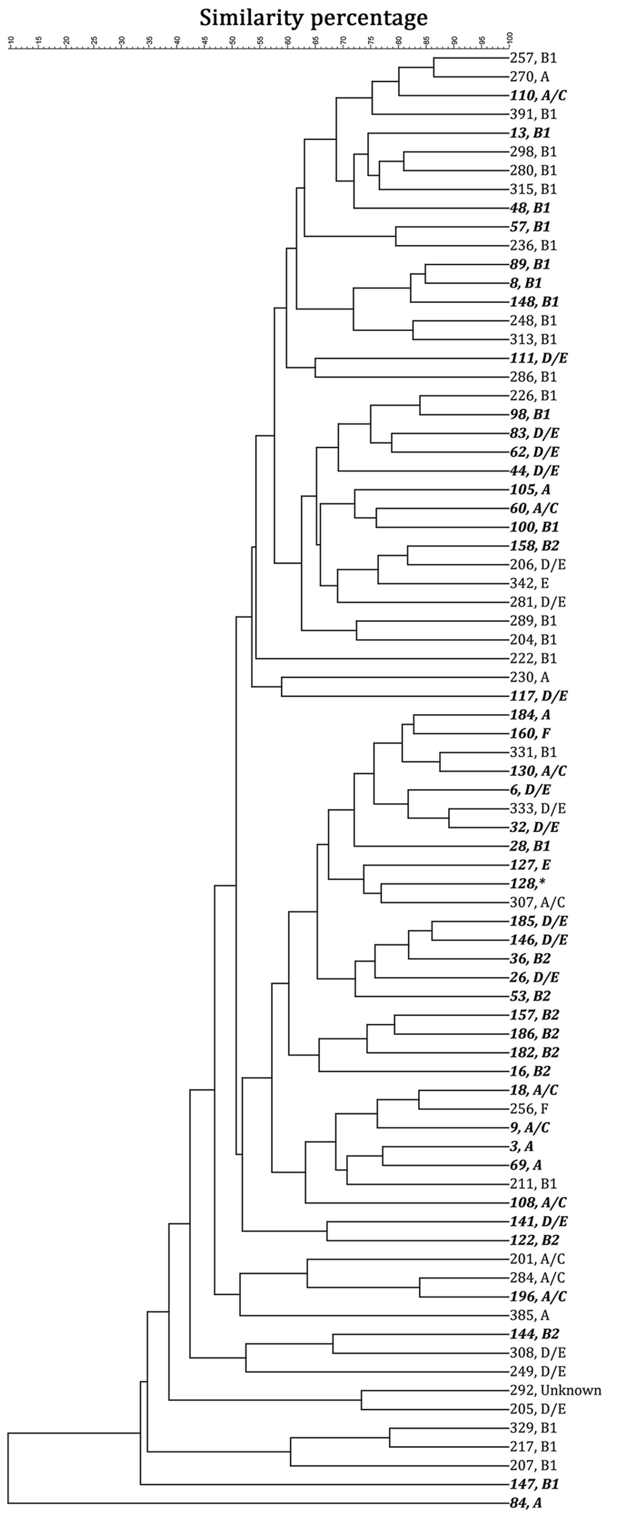

FIGURE 2 | The 78 strains selected for characterization showed less than 90% genomic similarity, based on a unweighted pair groups with mathematical averages (UPGMA) cluster analysis. Strains 1–200 (italic and bold) were collected from stream sediment; while 201–400 were collected from stream water. The strain number is followed by the phylotype analysis. *Strain 128 could not be recovered for phylotype analysis.

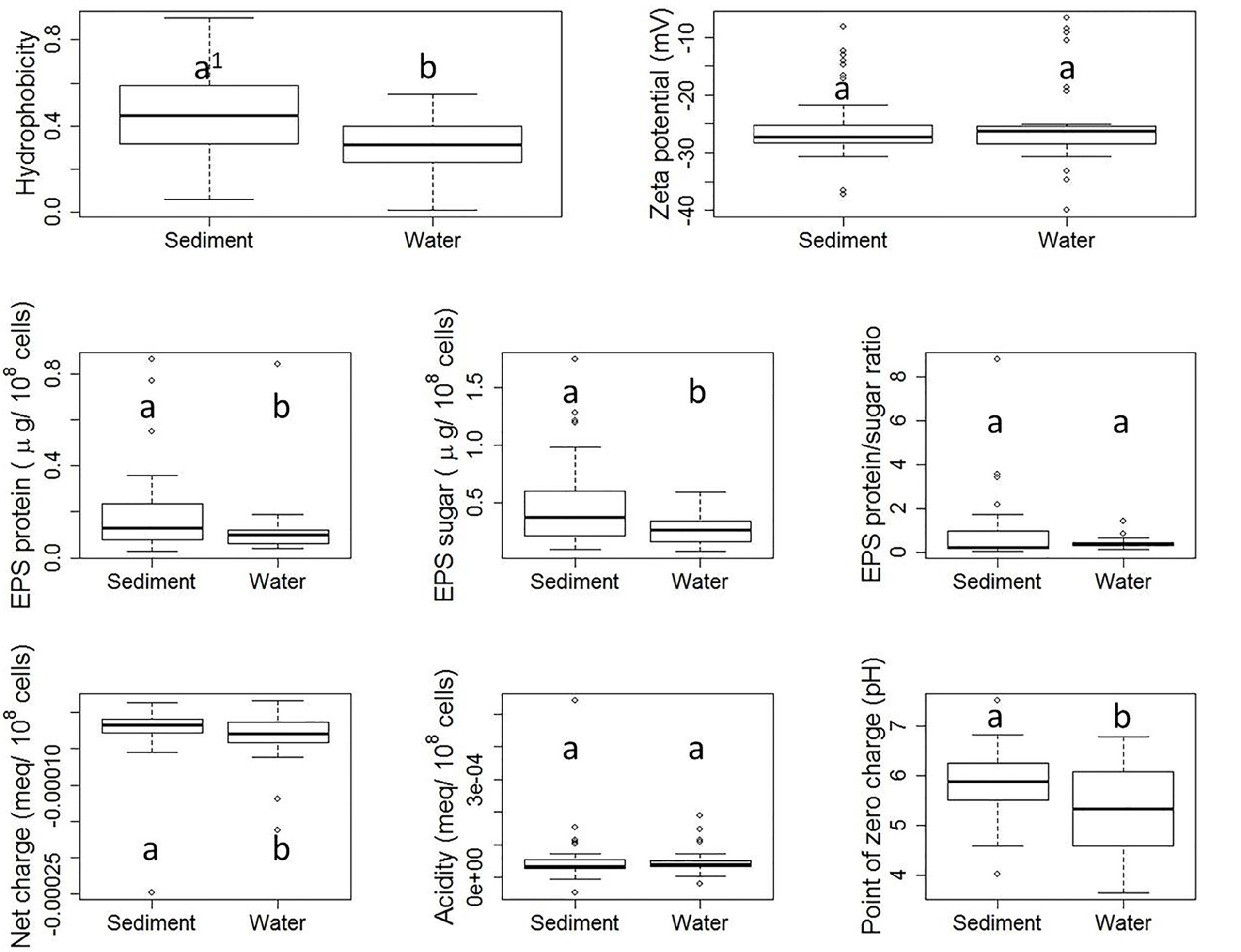

FIGURE 3 | Boxplots of property results from stream sediment *E. coli* and stream water *E. coli*. Each plot shows five numerical values: the smallest observation ([Q1 = Q2 − 1.5(Q4 − Q1)], the low end of the whisker), 25% quartile (Q2, low boundary of box), median (Q3, the band near the middle of box), 75% quartile (Q4, high boundary of box), and largest observation ([Q5 = Q4 + 1.5(Q4 − Q1)], the high end of the whisker). Outliers, if any, are indicated by dots. Within each subplot, values with the same letter are not different at the significant level. The difference is determined by a Wilcoxon test with significance level set at $\alpha = 0.05$.

from stream water and sediment were observed, as shown by **Figure 3**. Hydrophobicity, as measured by the MATH assay, ranged from 0.01 to 0.90. Zeta potential ranged from −6.76 to −39.87 mV. Total EPS protein content ranged from 0.30 to 0.86 μg/10^8 cells, while total sugar content of EPS ranged from 0.80 to 1.74 μg/10^8 cells. The EPS protein/sugar ratio ranged from 0.07 to 8.78. Net charge at pH 8.0 varied from -2.48×10^{-4} to 1.60×10^{-5} meq/10^8 cells. Variation was also observed in the total acidity and PZC. While previous studies have reported surface property ranges for *E. coli* isolates, here we have measured all of these properties for this large number of distinct isolates. This framework could be applied to other organisms.

Figure 3 shows the boxplots of property results analyzed for sediment *E. coli* strains and water *E. coli* considered separately. Statistically significant differences in cell properties were observed between stream sediment *E. coli* and water *E. coli* in many cases. For example, according to the Wilcoxon test, sediment *E. coli* had a significantly greater hydrophobicity than water *E. coli* (p-value = 0.005). Previously, Stenstrom (1989) and Zita and Hermansson (1997), reported that higher hydrophobicity coincided with greater adhesion to mineral particles in water and sludge flocs in sludge liquor from wastewater treatment plants. In addition to hydrophobicity, sediment *E. coli* also had greater EPS protein content (p-value = 0.006) and sugar content (p-value = 0.036), less negative net charge (p-value = 0.026), and higher PZC (p-value = 0.009) than stream water *E. coli*.

The zeta potential of suspended *E. coli* cells reflects the electrokinetic potential of the cell surfaces. Colloid stability (i.e., the likelihood that cells will not coagulate with one another) will increase as the absolute value of the zeta potential increases. Previous studies have indicated that zeta

potential ranged from −4.9 to −29 mV for 280 different *E. coli* strains (Morrow et al., 2005) or ranged from −3.5 to −49 mV for 12 different *E. coli* strains (Bolster et al., 2009), which are similar with the range of our results. In our study, there was no significant difference between zeta potentials measured for sediment *E. coli* strains and water *E. coli* strains. Similarly, previous research has found no clear correlation between the zeta potential of bacterial cells and their adhesion to a negatively charged polystyrene surface (van Loosdrecht et al., 1987) or quartz particles (Bolster et al., 2009).

Our results also indicated that stream sediment *E. coli* strains had significantly higher EPS protein and sugar content than stream water strains, which is consistent with the fact that cell adhesion and biofilm formation require EPS (Dogsa et al., 2005; Vu et al., 2009). The EPS protein/sugar ratio can be dramatically impacted by environmental pH (Dogsa et al., 2005), culturing time (Shin et al., 2001), culturing medium, and extraction method (Nielsen Per and Jahn, 1999; Sheng et al., 2010). However, while the absolute values of EPS protein and sugar contents differed between the two habitats, no significant difference in the EPS protein/sugar ratio was observed in our study.

Moreover, the net charge at pH 8.0 of stream sediment *E. coli* was significantly less negative than the net charge of water *E. coli*. *E. coli* cells in both water and sediment carry an overall negative surface charge at pH 8.0. Those strains carrying more negative net charge need to overcome a greater barrier from electrostatic force when attaching to negatively charged sediment surfaces, as demonstrated in previous studies (Dickson and Koohmaraie, 1989; Bolster et al., 2009). The cell surfaces of sediment *E. coli* also had a lower PZC.

E. coli Property Correlations

Correlation analyses were used to determine whether any two measured cell properties were dependent. Pair-wise correlations between the different cell properties measured in this study were generally low and not statistically significant when analyzed for all 77 *E. coli* strains (**Table 1**). Of the statistically significant correlations observed in this study, some were between properties which had shared parameters or measurements so the correlations were artificially inflated. Such correlations include: the EPS protein/sugar ratio with EPS protein and EPS sugar, net charge with acidity, and net charge with PZC. On the other hand, the strong positive correlation between hydrophobicity and EPS protein content [r (correlation coefficient) $= 0.283$; p-value $= 3.914 \times 10^{-4}$] is novel and useful. Interestingly, the correlation between hydrophobicity and the EPS protein was not the same for stream sediment *E. coli* and water *E. coli*. Using scatterplots with smoothing curves, histograms of each property, and the results from the Kendall-tau correlation method, **Figure 4** shows the correlations between *E. coli* hydrophobicity and EPS protein content for stream sediment *E. coli* and water *E. coli*, respectively. For sediment *E. coli*, there was significant positive correlation ($r = 0.407$, p-value $= 1.274 \times 10^{-4}$) between hydrophobicity and the EPS protein; while for water *E. coli*, no significant correlation was observed ($r = -0.103$ with p-value $= 0.416$). Previous research has found that hydrophobic components of EPS are mainly comprised of proteins (Jorand et al., 1998; Gerbersdorf et al., 2008). Perhaps the identity or abundance of those EPS proteins lead to the partitioning of these strains into the sediment where they can be protected from environmental stresses such as ultraviolet radiation. Additional studies to characterize EPS proteins associated with the two groups of *E. coli* would be helpful to determine the mechanisms behind these observations.

By definition, surface charge and zeta potential are related. However, our results indicate only a very weak correlation between net charge and zeta potential with $r = 0.008$ with p-value $= 0.316$. Thus our results may temper the conclusions of some previous research in which zeta potential has been used to estimate surface charge (Alves et al., 2010; Zhang et al., 2012).

Correlation between Genomic Similarity with Environmental Habitat

To explore possible relationships between *E. coli*-$(GTG)_5$ genomic similarity and environmental habitat (stream sediment or water), the Mantel test was applied. The results showed a significantly positive correlation between genomic similarity and environmental habitat ($r = 0.063$; p-value $= 0.002$). This finding indicates that genomic similarity was larger within habitat, as

TABLE 1 | Correlation coefficient matrix for cell properties for all *E. coli* strains ($n = 77$) obtained from Kendall-tau correlation method.

	Hydrophobicity	Zeta potential	EPS protein	EPS sugar	Ratio (EPS protein/sugar)	Net charge at pH 8.0	Acidity
Zeta potential	−0.047						
Extracellular polymeric substance (EPS) protein	***0.283***	−0.179					
EPS sugar	−0.060	−0.177	0.095				
Ratio (EPS protein/sugar)	0.203	−0.016	***0.403***	***−0.522***			
Net charge at pH 8.0	0.131	0.078	0.035	−0.141	0.066		
Acidity	0.016	−0.085	0.148	0.064	0.078	***−0.540***	
Point of zero charge	0.237	0.075	0.149	−0.121	0.136	***0.403***	−0.170

Statistically significant (p < 0.001) are in italic and bold.

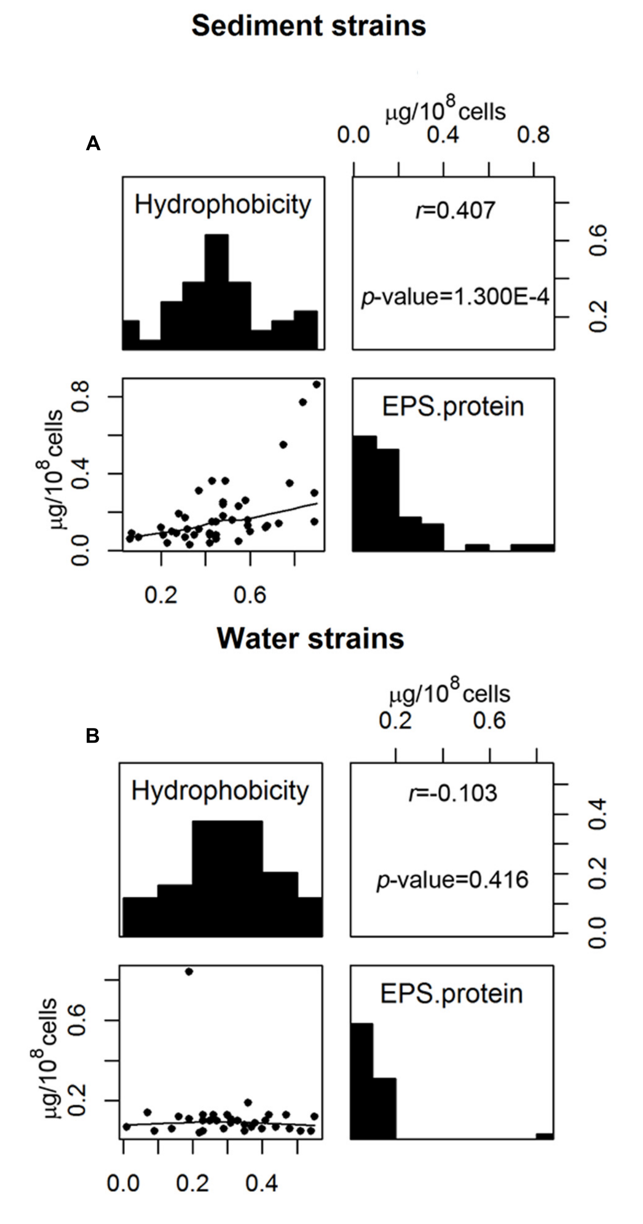

FIGURE 4 | Correlation between *E. coli* hydrophobicity and EPS protein content for (A) sediment *E. coli* strains and (B) water *E. coli* strains. The upper panels show the correlation coefficient *r* with associated *p*-value analyzed by Kendall-tau method; the diagonal panels show histograms of each property; the lower panels are scatterplots with the smoothing curves using the Lowess method.

compared to between habitat types. Thus, genomic factors may ultimately influence whether certain strains remain in the water or sediment.

E. coli Property Comparison Integrating Phylogenetic Correlation

Phylogenetic generalized least square method was employed to explore the impact of environmental habitat (extrinsic) on bacterial properties, while excluding potential impacts from genomic similarity (intrinsic). The results indicate that some bacterial properties were significantly regulated by environmental habitat: e.g., *E. coli* strains occurring in stream sediments were more likely to have higher hydrophobicity (p-value $= 2.935 \times 10^{-7}$), EPS sugar content (p-value $= 3.953 \times 10^{-3}$), net charge (p-value $= 3.414 \times 10^{-4}$), and PZC (p-value $= 6.165 \times 10^{-8}$), but lower acidity (p-value $= 0.016$) when compared to *E. coli* strains suspended in the water column.

Previous studies have been unable to determine if differences in environmental *E. coli* cell surface properties and genomic variation residing in different environmental habitats (stream bottom sediments versus overlying water) are due primarily to environmental habitat (extrinsic), genomic similarity (intrinsic), or an interaction of these two. Below is a summary of our major conclusions:

- Statistically significant differences in cell properties were observed between stream sediment *E. coli* and water *E. coli*; most notably, sediment *E. coli* had significantly greater hydrophobicity, EPS protein content, and EPS sugar content; less negative net charge; and higher PZC when compared to water *E. coli*.
- Hydrophobicity and EPS protein were positively correlated for stream sediment *E. coli* but not for water *E. coli*.
- Genomic similarity was greater within environmental habitat, as compared to between habitat types.
- When the impacts of genomic similarity were accounted for, the impact of environmental habitat on hydrophobicity, EPS sugar, net charge, PZC, and acidity was significant among the strains, indicating that habitat was a regulating factor for expression of these properties.

AUTHOR CONTRIBUTIONS

CL, XL, and MS collected strains. XL created **Figure 1**, performed the experiments for and analyzed the data presented in **Figures 2–5**, wrote the manuscript draft, and finalized the manuscript. CL performed rep-PCR. MT, MS, and LJ contributed to experimental design, data analysis, and manuscript writing. PD contributed to statistical analysis.

FUNDING

Funding for this work was provided by the National Science Foundation (CBET-1236510); opinions, findings, and conclusions are those of the authors and do not necessarily reflect the views of the National Science Foundation.

ACKNOWLEDGMENT

We thank Dr. Teresita Chua for assistance with the titration measurements, Dr. Jean-Philippe Tessonnier for assistance with zeta potential measurements, Dr. Cathy Logue for assistance with rep-PCR, and Dr. Ganwu Li for phylo-type analysis.

SUPPLEMENTARY MATERIAL

The Supplementary Material for this article can be found online at: http://journal.frontiersin.org/article/10.3389/fmicb.2016.01732/full#supplementary-material

FIGURE A1 | Potentiometric titration curve example from strain number 53. Three pieces of information can be obtained from the curve: (1) Point of zero charge of *E. coli* strain = pH 5.8; (2) Net charge at pH 8.0 carried by *E. coli* = -7.5×10^{-6} meq/10^8 cells; (3) Total acidity of *E. coli* strain = 2.8×10^{-5} meq/10^8 cells.

FIGURE A2 | The dendrogram shows the percent similarity of rep-PCR fingerprint banding patterns for 78 strains with similarity smaller than 90%, based on UPGMA cluster analysis. Strains 1–200 (italic and bold) were collected from stream sediment, while 201–400 were collected from stream water.

REFERENCES

Alves, C. S., Melo, M. N., Franquelim, H. G., Ferre, R., Planas, M., Feliu, L., et al. (2010). *Escherichia coli* cell surface perturbation and disruption induced by antimicrobial peptides BP100 and pepR. *J. Biol. Chem.* 285, 27536–27544. doi: 10.1074/jbc.M110.130955

Bai, S., and Lung, W. S. (2005). Modeling sediment impact on the transport of fecal bacteria. *Water Res.* 39, 5232–5240. doi: 10.1016/j.watres.2005.10.013

BioNumerics. (2013). *BioNumerics Tutorial: Clustering Fingerprint Data*. Available: http://www.applied-maths.com/sites/default/files/whitepapers/clustering_fingerprint_data.pdf

Bitton, G., Henis, Y., and Lahav, N. (1972). Effect of several clay-minerals and humic acid on survival of kiebiella-aerogenes exposed to ultrviolet-irradiation. *Appl. Microbiol.* 23, 870–874.

Bolster, C. H., Haznedaroglu, B. Z., and Walker, S. L. (2009). Diversity in cell properties and transport behavior among 12 different environmental *Escherichia coli* isolates. *J. Environ. Qual.* 38, 465–472. doi: 10.2134/jeq2008.0137

Bruckner, C. G., Rehm, C., Grossart, H. P., and Kroth, P. G. (2011). Growth and release of extracellular organic compounds by benthic diatoms depend on interactions with bacteria. *Environ. Microbiol.* 13, 1052–1063. doi: 10.1111/j.1462-2920.2010.02411.x

Chang, W.-S. (2005). *Influence of Reduced Water Availability on Pseudomonas Putida Unsaturated Biofilms and the Role of Alginate in Desiccation Tolerance*. Doctor of Philosopy thesis, Iowa State University, Iowa.

Clermont, O., Christenson, J. K., Denamur, E., and Gordon, D. M. (2013). The clermonth *Escherichia coli* phylo-typing method revisited: improvemetn of specificity and detection of new phylo-groups. *Environ. Microbiol. Rep.* 5, 58–65. doi: 10.1111/1758-2229.12019

Cook, K. L., Bolster, C. H., Ayers, K. A., and Reynolds, D. N. (2011). *Escherichia coli* diversity in livestock manures and agriculturally impacted stream waters. *Curr. Microbiol.* 63, 439–449. doi: 10.1007/s00284-011-0002-6

Cortes, P., Blanc, V., Mora, A., Dahbi, G., Blanco, J. E., Blanco, M., et al. (2010). Isolation and characterization of potentially pathogenic antimicrobial-resistant *Escherichia coli* strains from chicken and pig farms in Spain. *Appl. Environ. Microbiol.* 76, 2799–2805. doi: 10.1128/AEM.02421-09

Davies, C. M., and Bavor, H. J. (2000). The fate of storm water associated bacteria in constructed wetland and water pollution control pond systems. *Appl. Microbiol.* 89, 349–360. doi: 10.1046/j.1365-2672.2000.01118.x

Dickson, J. S., and Koohmaraie, M. (1989). Cell surface charge characteristics and their relationship to bacterial attachment to meat surfaces. *Appl. Environ. Microbiol.* 55, 832–836.

Dogsa, I., Kriechbaum, M., Stopar, D., and Laggner, P. (2005). Structure of bacterial extracellular polymeric substances at different pH values as determined by SAXS. *Biophys. J.* 89, 2711–2720. doi: 10.1529/biophysj.105.061648

Dubois, M., Gilles, K. A., Hamilton, J. K., Rebers, P. A., and Smith, F. (1956). Colorimetric method for determination of sugars and related substances. *Anal. Chem.* 28, 350–356. doi: 10.1021/ac60111a017

Fein, J. B., Boily, J. F., Yee, N., Gorman-Lewis, D., and Turner, B. F. (2005). Potentiometric titrations of *Bacillus subtilis* cells to low pH and a comparison of modeling approaches. *Geochim. Cosmochim. Acta* 69, 1123–1132. doi: 10.1016/j.gca.2004.07.033

Gerbersdorf, S. U., Manz, W., and Paterson, D. M. (2008). The engineering potential of natural benthic bacterial assemblages in terms of the erosion resistance of sediments. *FEMS Microbiol. Ecol.* 66, 282–294. doi: 10.1111/j.1574-6941.2008.00586.x

Goulter, R. M., Gentle, I. R., and Dykes, G. A. (2009). Issues in determining factors influencing bacterial attachment: a review using the attachment of *Escherichia coli* to abiotic surfaces as an example. *Lett. Appl. Microbiol.* 49, 1–7. doi: 10.1111/j.1472-765X.2009.02591.x

Hipsey, M. R., Brookes, J. D., Regel, R. H., Antenucci, J. P., and Burch, M. D. (2006). In situ evidence for the association of total coliforms and *Escherichia coli* with suspended inorganic particles in an Australian reservoir. *Water Air Soil Poll* 170, 191–209. doi: 10.1007/s11270-006-3010-6

Hoa, P. T., Nair, L., and Visvanathan, C. (2003). The effect of nutrients on extracellular polymeric substance production and its influence on sludge properties. *Water SA* 29, 437–442.

Im, S.-A., Wang, W., Lee, C.-K., and Lee, Y. N. (2010). Activation of macrophages by exopolysaccharide produced by MK1 bacterial strain isolated from neungee mushroom, Sarcodon aspratus. *Immune Netw.* 10, 230–238. doi: 10.4110/in.2010.10.6.230

Jamieson, R., Joy, D. M., Lee, H., Kostaschuk, R., and Gordon, R. (2005). Transport and deposition of sediment-associated *Escherichia coli* in natural streams. *Water Res.* 39, 2665–2675. doi: 10.1016/j.watres.2005.04.040

Jeng, H. W. C., England, A. J., and Bradford, H. B. (2005). Indicator organisms associated with stormwater suspended particles and estuarine sediment. *J. Environ. Sci. Health A Tox Hazard Subst. Environ. Eng.* 40, 779–791. doi: 10.1081/ese-20048264

Jorand, F., Boue-Bigne, F., Block, J. C., and Urbain, V. (1998). Hydrophobic/hydrophilic properties of activated sludge exopolymeric substances. *Water Sci. Technol.* 37, 307–315. doi: 10.1016/S0273-1223(98)00123-1

Khemakhem, W., Ammar, E., and Bakhrouf, A. (2005). Effect of environmental conditions on hydrophobicity of marine bacteria adapted to textile effluent treatment. *World J. Microbiol. Biotechnol.* 21, 1623–1631. doi: 10.1007/s11274-005-8537-y

Liang, X., Soupir, M. L., Rigby, S., Jarboe, L. R., and Zhang, W. (2014). Flow cytometry is a promising and rapid method for differentiating between freely suspended *Escherichia coli* and *E. coli* attached to clay particles. *J. Appl. Microbiol.* 117, 1730–1739. doi: 10.1111/Jam.12660

Liao, C., Liang, X., Soupir, M. L., and Jarboe, L. R. (2015). Cellular, particle and environmental parameters influencing attachment in surface waters: a review. *J. Appl. Microbiol.* 119, 315–330. doi: 10.1111/jam.12860

Lowry, O. H., Rosebrough, N. J., Farr, A. L., and Randall, R. J. (1951). Protein measurement with the folin phenol reagent. *J. Biol. Chem.* 193, 265–275.

Lu, Z. X., Lapen, D., Scott, A., Dang, A., and Topp, E. (2005). Identifying host sources of fecal pollution: diversity of *Escherichia coli* in confined dairy and swine production systems. *Appl. Environ. Microbiol.* 71, 5992–5998. doi: 10.1128/aem.71.10.5992-5998.2005

Ma, H. J., Fu, L. L., and Li, J. R. (2011). Differentiation of fecal *Escherichia coli* from human, livestock, and poultry sources by rep-PCR DNA fingerprinting on the shellfish culture area of East China Sea. *Curr. Microbiol.* 62, 1423–1430. doi: 10.1007/s00284-011-9870-z

Mao, X. (2015). *Generalized Least Square in Comparative Phylogenetics*. Available: http://cran.r-project.org/web/packages/pGLS/pGLS.pdf

Mohapatra, B. R., Broersma, K., and Mazumder, A. (2008). Differentiation of fecal *Escherichia coli* from poultry and free-living birds by (GTG)(5)-PCR genomic fingerprinting. *Int. J. Med. Microbiol.* 298, 245–252. doi: 10.1016/j.ijmm.2007.03.019

Mohapatra, B. R., and Mazumder, A. (2008). Comparative efficacy of five different rep-PCR methods to discriminate *Escherichia coli* populations in aquatic environments. *Water Sci. Technol.* 58, 537–547. doi: 10.2166/Wst.2008.424

Morrow, J. B., Stratton, R., Yang, H. H., Smets, B. F., and Grasso, D. (2005). Macro–and nanoscale observations of adhesive behavior for several *E. coli* Strains (O157:H7 and environmental isolates) on mineral surfaces. *Environ. Sci. Technol.* 39, 6395–6404. doi: 10.1021/es0500815

Nielsen Per, H., and Jahn, A. (1999). "Extraction of EPS," in *Microbial Extracellular Polymeric Substances: Characterization, Structure and Function*, eds N. T. Wingender and H. C. Flemming (Berlin: Springer-Verlag), 49–72.

Pachepsky, Y. A., Yu, O., Karns, J. S., Shelton, D. R., Guber, A. K., and van Kessel, J. S. (2008). Strain-dependent variations in attachment of E. *coli* to soil particles of different sizes. *Int. Agrophys.* 22, 61–66.

Pandey, P. K., and Soupir, M. L. (2013). Assessing the impacts of E. *coli* laden streambed sediment on *E. coli* loads over a range of flows and sediment characteristics. *J. Am. Water Resour. Assoc.* 49, 1261–1269. doi: 10.1111/Jawr.12079

Pandey, P. K., Soupir, M. L., and Rehmann, C. R. (2012). A model for predicting resuspension of *Escherichia coli* from streambed sediments. *Water Res.* 46, 115–126. doi: 10.1016/j.watres.2011.10.019

Pembrey, R. S., Marshall, K. C., and Schneider, R. P. (1999). Cell surface analysis techniques: what do cell preparation protocols do to cell surface properties? *Appl. Environ. Microbiol.* 65, 2877–2894.

Rademaker, J., and de Bruijn, F. (1997). "Characterization and classification of microbes by rep-PCR genomic fingerprinting and computer assisted pattern analysis," in *DNA Markers: Protocols, Applications nad Overviews*, eds G. Caetano-Anoiies and P. M. Gresshoff (New York, NY: Wiley & Sons), 151–171.

Revell, L. J. (2010). Phylogenetic signal and linear regression on species data. *Methods Ecol. Evol.* 1, 319–329. doi: 10.1111/j.2041-210X.2010.00044.x

Rosenberg, M., Gutnick, D., and Rosenberg, E. (1980). Adherence of bacteria to hydrocarbons–a simple method for measuring cell-surface hydrophobicity. *FEMS Microbiol. Lett.* 9, 29–33. doi: 10.1111/j.1574-6968.1980.tb05599.x

Schillinger, J. E., and Gannon, J. J. (1985). Bacterial adsorption and suspned particles in urban stormwater. *Water Pollut. Control Fed.* 57, 384–389. doi: 10.1016/j.watres.2015.08.026

Schulz, J., Ruddat, I., Hartung, J., Hamscher, G., Kemper, N., and Ewers, C. (2016). Antimicrobial-resistant *Escherichia* coil survived in dust samples for more than 20 years. *Front. Microbiol.* 7:10. doi: 10.3389/fmicb.2016.00866

Sheng, G. P., Yu, H. Q., and Li, X. Y. (2010). Extracellular polymeric substances (EPS) of microbial aggregates in biological wastewater treatment systems: a review. *Biotechnol. Adv.* 28, 882–894. doi: 10.1016/j.biotechadv.2010.08.001

Shin, H. S., Kang, S. T., and Nam, S. Y. (2001). Effect of carbohydrate and protein in the EPS on sludge settling characteristics. *Water Sci. Technol.* 43, 193–196.

Song, L. F., Johnson, P. R., and Elimelech, M. (1994). Kinetics of colloid deposition onto heterogeneously charged surfaces in porous-meida. *Environ. Sci. Technol.* 28, 1164–1171. doi: 10.1021/es00055a030

Stenstrom, T. A. (1989). Bacterial hydrophobicity, an overall parameter for the measurement of adhesion potential to soil particles. *Appl. Environ. Microbiol.* 55, 142–147.

USEPA (2002). *Method 1603: Escherichia coli* (*E. coli*) in Water by Membrane Filtration using Modified Membrane-Thermotolerant *Escherichia coli* Agar (Modified mTEC). Washington DC: US Environmental Protection Agency Office of Water.

USEPA (2014). *Watershed Assessment, Tracking & Environmental Results*. Washington DC: US Environmental Protection Agency Office of Water.

van Loosdrecht, M. C. M., Lyklema, J., Norde, W., Schraa, G., and Zehnder, A. J. B. (1987). Electrophoretic mobility and hydrophobicity as a measure to predict the initial steps of bacteria adhersion. *Appl. Environ. Microbiol.* 53, 1898–1901.

Vandermei, H. C., Desoet, J. J., Degraaff, J., Rouxhet, P. G., and Busscher, H. J. (1991). Comparison of the physicochemical surface properties of Streptococcus rattus with those of other mutans streptococcal species. *Caries Res.* 25, 415–423. doi: 10.1159/000261404

Vu, B., Chen, M., Crawford, R. J., and Ivanova, E. P. (2009). Bacterial extracellular polysaccharides involved in biofilm formation. *Molecules* 14, 2535–2554. doi: 10.3390/molecules14072535

Walker, S. L., Redman, J. A., and Elimelech, M. (2005). Influence of growth phase on bacterial deposition: Interaction mechanisms in packed-bed column and radial stagnation point flow systems. *Environ. Sci. Technol.* 39, 6405–6411. doi: 10.1021/es050077t

Warnes, S. L., Caves, V., and Keevil, C. W. (2012). Mechanism of copper surface toxicity in *Escherichia coli* O157:H7 and *Salmonella* involves immediate membrane depolarization followed by slower rate of DNA destruction which differs from that observed for Gram-positive bacteria. *Environ. Microbiol.* 14, 1730–1743. doi: 10.1111/j.1462-2920.2011.02677.x

Wilen, B. M., Jin, B., and Lant, P. (2003). Relationship between flocculation of activated sludge and composition of extracellular polymeric substances. *Water Sci. Technol.* 47, 95–103.

Zhang, W., Hughes, J., and Chen, Y. S. (2012). Impacts of hematite nanoparticle exposure on biomechanical, adhesive, and surface electrical properties of *Escherichia coli* cells. *Appl. Environ. Microbiol.* 78, 3905–3915. doi: 10.1128/Aem.00193-12

Zhao, W., Walker, S. L., Huang, Q., and Cai, P. (2014). Adhesion of bacterial pathogens to soil colloidal particles: influences of cell type, natural organic matter, and solution chemistry. *Water Res.* 53, 35–46. doi: 10.1016/j.watres.2014.01.009

Zikmanis, P., Shakirova, L., Auzina, L., and Andersone, I. (2007). Hydrophobicity of bacteria *Zymomonas mobilis* under varied environmental conditions. *Process Biochem.* 42, 745–750. doi: 10.1016/j.procbio.2007.01.002

Zita, A., and Hermansson, M. (1997). Effects of bacterial cell surface structures and hydrophobicity on attachment to activated sludge flocs. *Appl. Environ. Microbiol.* 63, 1168–1170.

Conflict of Interest Statement: The authors declare that the research was conducted in the absence of any commercial or financial relationships that could be construed as a potential conflict of interest.

The reviewer GDO and handling Editor declared their shared institutional affiliation and the handling Editor states that the process nevertheless met the standards of a fair and objective review.

Copyright © 2016 Liang, Liao, Thompson, Soupir, Jarboe and Dixon. This is an open-access article distributed under the terms of the Creative Commons Attribution License (CC BY). The use, distribution or reproduction in other forums is permitted, provided the original author(s) or licensor are credited and that the original publication in this journal is cited, in accordance with accepted academic practice. No use, distribution or reproduction is permitted which does not comply with these terms.
