# Supplementary material for: Escherichia coli attachment to model particulates: The effects of bacterial cell characteristics and particulate properties
Source: PLoS One. 2017 Sep 14;12(9):e0184664. doi: 10.1371/journal.pone.0184664 (PMC5599003; doi:10.1371/journal.pone.0184664)
Supplement: S2 File — (PDF) [file pone.0184664.s006.pdf]

frontiers in Microbiology

ORIGINAL RESEARCH
published: 03 May 2017
doi: 10.3389/fmicb.2017.00708

# Allelic Variation in Outer Membrane Protein A and Its Influence on Attachment of *Escherichia coli* to Corn Stover

**Chunyu Liao[1], Xiao Liang[2], Fan Yang[2], Michelle L. Soupir[1,2], Adina C. Howe[1,2], Michael L. Thompson[3] and Laura R. Jarboe[1,4]***

[1] Interdepartmental Microbiology Program, Iowa State University, Ames, IA, USA, [2] Department of Agricultural and Biosystems Engineering, Iowa State University, Ames, IA, USA, [3] Department of Agronomy, Iowa State University, Ames, IA, USA, [4] Department of Chemical and Biological Engineering, Iowa State University, Ames, IA, USA

Understanding the genetic factors that govern microbe-sediment interactions in aquatic environments is important for water quality management and reduction of waterborne disease outbreaks. Although chemical properties of bacteria have been identified that contribute to initiation of attachment, the outer membrane proteins that contribute to these chemical properties still remain unclear. In this study we explored the attachment of 78 *Escherichia coli* environmental isolates to corn stover, a representative agricultural residue. Outer membrane proteome analysis led to the observation of amino acid variations, some of which had not been previously described, in outer membrane protein A (OmpA) at 10 distinct locations, including each of the four extracellular loops, three of the eight transmembrane segments, the proline-rich linker and the dimerization domain. Some of the polymorphisms within loops 1, 2, and 3 were found to significantly co-occur. Grouping of sequences according to the outer loop polymorphisms revealed five distinct patterns that each occur in at least 5% of our isolates. The two most common patterns, I and II, are encoded by 33.3 and 20.5% of these isolates and differ at each of the four loops. Statistically significant differences in attachment to corn stover were observed among isolates expressing different versions of OmpA and when different versions of OmpA were expressed in the same genetic background. Most notable was the increased corn stover attachment associated with a loop 3 sequence of SNFDGKN relative to the standard SNVYGKN sequence. These results provide further insight into the allelic variation of OmpA and implicate OmpA in contributing to attachment to corn stover.

**Keywords: OmpA, attachment, polymorphism, allelic variation, competitive attachment assay, polymorphism co-occurrence**

OPEN ACCESS

**Edited by:**
*Martin G. Klotz, Queens College (CUNY), USA*

**Reviewed by:**
*Adrian Goldman, University of Helsinki, Finland*
*Anne-Marie Krachler, University of Texas Health Science Center at Houston, USA*

***Correspondence:**
*Laura R. Jarboe*
*ljarboe@iastate.edu*

**Specialty section:**
*This article was submitted to Microbial Physiology and Metabolism, a section of the journal Frontiers in Microbiology*

**Received:** *22 June 2016*
**Accepted:** *05 April 2017*
**Published:** *03 May 2017*

**Citation:**
*Liao C, Liang X, Yang F, Soupir ML, Howe AC, Thompson ML and Jarboe LR (2017) Allelic Variation in Outer Membrane Protein A and Its Influence on Attachment of Escherichia coli to Corn Stover. Front. Microbiol. 8:708. doi: 10.3389/fmicb.2017.00708*

## INTRODUCTION

Waterborne diseases such as gastroenteritis, diarrhea, typhoid fever, and dermatitis can develop when people consume or come into contact with water contaminated by pathogenic microorganisms (Rosen, 2001; Pond, 2005). In the United States, more than 562,000 miles of rivers and streams and 12.1 million acres of lakes, reservoirs and ponds are classified as impaired due

to pathogens (EPA)[1]. Worldwide, approximately 1.2 million people, about one quarter of whom were under 6 years of age, died from diarrheal diseases in 2013 (Murray et al., 2014). These diarrheal deaths can be attributed to a variety of microbial causes, including viruses, bacteria and amoeba. *Escherichia coli*, which is the focus of the research described here, was the known causative agent in more than 10% of the cases in which the etiology was determined (Murray et al., 2014). Bacteria in aquatic environments can attach to sediment particles, contributing to their persistence and transport (Schillinger and Gannon, 1985; Song et al., 1994; Davies and Bavor, 2000; Bai and Lung, 2005; Liao et al., 2015). To guide planning for water quality management and reduction of waterborne disease outbreaks, improved understanding of microbe-particle interactions is essential.

Bacterial attachment to a surface requires overcoming the repulsive forces between the cell and the surface. This surface attachment is the initial step of the highly ordered process of biofilm formation (Donlan, 2002; Van Houdt and Michiels, 2005). A variety of genes that encode or regulate cell surface structures have been demonstrated to be involved in biofilm formation, including, for example, *motA*, *crsA*, *fimA*, *ag43*, *csgA*, *ompX,* and *pgaABCD* (Pratt and Kolter, 1998; Vidal et al., 1998; Hare and McDonough, 1999; Danese et al., 2000; Jackson et al., 2002; Beloin et al., 2004; Otto and Hermansson, 2004). Such genes may also affect the initiation of attachment, given the fact that increased attachment leads to more frequent establishment of biofilms (Agladze et al., 2005). The Derjaguin-Landau-Verwey-Overbeek (DLVO) theory has been reported to efficiently predict bacteria-mineral interactions, in which the interaction force is a function of both cell surface net charge and hydrophobicity (Rijnaarts et al., 1999; Hori and Matsumoto, 2010; Zhao et al., 2014). In this context, cell surface net charge and cell surface hydrophobicity are regarded as two chemical properties that contribute to microbial attachment to solids, though some studies suggest that the contribution of hydrophobicity and surface charge may differ depending on the surface in question (Rivas et al., 2007; Bolster et al., 2009; Liao et al., 2015). Our previous characterization of 78 environmental *E. coli* isolates shows a range of cell surface charge from −39.9 to −6.8 mV in terms of zeta potential and hydrophobicity from 0.01 to 0.9 (Liang et al., 2016). These studies also showed that while cell surface hydrophobicity can be attributed to variations in the production of extracellular polymeric substances, the variations in cell surface charge did not correlate with any of the other measured properties (Liang et al., 2016). This lack of understanding of the basis of variations in cell surface charge highlights the need for further characterization of genetic factors contributing to cell surface properties.

At the interface between the bacteria and particles, cell surface structures, such as outer membrane proteins, flagella, fimbria, and extracellular polysaccharides affect the initial attachment step by contributing to the chemical properties of the cell surface. Although biofilm development is well-characterized, little is known about preexisting surface structures that mediate the initial interaction between bacteria and particles in the aquatic habitats. Moreover, while there are many studies about attachment of bacteria to plant tissues and quartz (sand) in lab conditions, little is known about the genetic factors that drive the attachment of *E. coli* to representative stream particles under environmentally relevant conditions. Proteomic analysis has proven to be an effective method to explore the surface structures which play key roles in attachment (Otto et al., 2001; Orme et al., 2006; Rivas et al., 2008).

More than 300 million acres of US farmland were planted with corn in 2015 (USDA, 2016). Production of the corn kernel is accompanied by the production of an approximately equal mass of corn stover, which includes such components as the stalks, cobs and husks (Adler et al., 2015). Corn stover consists of roughly 34 wt% glucan, 22 wt% xylan, and 18 wt% lignin and has a crystallinity index of 50 (Kumar et al., 2009). There are a variety of uses for the stover, including but not limited to, use as animal feed or for the production of biorenewable fuels and chemicals (Golecha and Gan, 2016). Here we have selected corn stover as a representative agricultural residue likely to be present in streams and sediment.

In this study, *E. coli* strains isolated from a local waterway were used to characterize the outer membrane proteins (OMPs) which contribute to the attachment of *E. coli* to corn stover under environmentally relevant conditions. This process was guided by the use of proteomic analysis and development of a competitive attachment assay, modified from previous work (Liu et al., 2011). This analysis includes consideration of flagella and OMPs, such as Outer Membrane Protein A (OmpA). Our work demonstrates the association of sequence variations within the OmpA protein with attachment to corn stover.

## MATERIALS AND METHODS

### Bacterial Strains, Growth Condition, and Corn Stover Particles

Seventy-eight *E. coli* strains were selected from a pool of 400 *E. coli* strains isolated from Squaw Creek in Ames, Iowa, as previously described (Liang et al., 2016). These *E. coli* isolates were of different genotype according to our previous repetitive sequence based-PCR (rep-PCR) and fingerprint profile analysis, using a maximum similarity cut-off of 90% (Liang et al., 2016). The *E. coli* lab strain MG1655 was used as a K12 control. Cells were grown in M9 medium (pH = 7.4) with 0.4% (wt/vol) glucose at 37°C, 250 rpm to early stationary phase ($OD_{600}$ = 1.0–1.5), and then harvested by centrifugation at 4°C, 4,000 × g for 15 min. This growth condition was selected with the intention of approximating the conditions of the mammalian hosts that are the typical source of bacteria in agricultural water systems.

This growth and harvesting condition was used in preparing cells for the attachment assay and outer membrane protein extraction, unless otherwise indicated. Cells for molecular

[1]EPA. Available: http://ofmpub.epa.gov/waters10/attains_nation_cy.control [Accessed].

manipulation were grown in Luria-Bertani (LB) medium (pH = 7.0), at 37°C or 30°C as needed. When required, the medium was supplemented with 25 $\mu$g ml$^{-1}$ chloramphenicol and 100 $\mu$g ml$^{-1}$ ampicillin.

Corn stover was prepared by grinding dry corn leaf. The resulting powder was sieved using U.S. standard no. 270. The resulting particles with diameter less than 53 $\mu$m were collected and stored at room temperature. This corn stover had a carbon content of 38 wt% and surface area of 3 m$^2$/g (Liang et al, in preparation).

## Transmission Electron Microscopy

Negative staining and TEM imaging were conducted in the Microscopy and NanoImaging Facility at Iowa State University. Briefly, 3 $\mu$l of cells in water were placed onto a carbon film coated copper grid for 1 min. The supernatant was removed by wicking from the side with a piece of filter paper. Then 3 $\mu$l of 2% (wt/vol) aqueous uranyl acetate was placed onto the grid for 30 s. Excess stain was removed by wicking, and the grid was allowed to dry. Images were made using a JEOL 2,100 scanning transmission electron microscope at an accelerating voltage of 200 kV (Japan Electron Optics Laboratory, USA, Peabody, MA).

## Motility Agar Stab Test

A motility agar stab test was performed as described previously (Greene et al., 1951). Briefly, *E. coli* strains were stabbed into glass tubes with LB containing 0.4% (wt/vol) agar. The tubes were incubated at 37°C for 18 h or until growth was evident. A diffuse cloud of growth beyond the stab track indicates motility, while growth constrained to the stab track indicates a lack of motility.

## Attachment Assays

Single-strain attachment assays were performed according to previously described methods (Liu et al., 2011; Zwonitzer et al., 2016) with modifications that are briefly described here. Fifteen $\mu$l of cell suspension were added to a 15-ml centrifuge tube (120 mm in length) containing 15 ml of saturated $CaCO_3$ solution (pH ~ 8) and 1.06 mg l$^{-1}$ corn stover. The target final *E. coli* concentration was 10$^3$ CFU ml$^{-1}$. These concentrations were selected based on previous reports (Terrio, 1996; Bai and Lung, 2005). After shaking at 80 rpm for 10 min, samples were allowed to settle for 1 min. The top 7.5 ml of liquid was transferred to another tube without disturbing the bottom layer. This top layer contained unattached bacteria. One drop of Tween 85 was added to the remaining liquid to separate the settled bacteria-particle aggregates. After vortexing for 10 s, each sample was diluted 10-fold with phosphate buffer. One ml of the diluted samples was then used for enumeration of colony forming units (CFU) on LB agar plates. Note that the Tween treatment and vortexing have no significant impact on cell viability (*data not shown*). The attached fraction was calculated as:

$$\text{Attached fraction} = (C_b - C_t)/((C_t + C_b))$$

where $C_t$ is the number of CFU collected from the top half of the settled liquid and $C_b$ is the number of CFU collected from an equal volume of the bottom half of the settled liquid after addition of Tween and vortexting. The ($C_b$ – $C_t$) term represents the enrichment of attached bacteria in the bottom half of the liquid relative to the unattached bacteria assumed to dispersed uniformly throughout the top and bottom halves of the liquid. The ($C_t + C_b$) term represents the total number of CFU queried. Up to six distinct tubes were assayed for each strain. An attached fraction was calculated for each tube, and then the average of all replicates was calculated. CFU counts ranged from 7 to 118, with an average value of 23.

When directly comparing two strains, such as a wild-type (WT) and mutant, we used a competitive attachment assay developed here. This assay is very similar to the single-strain assay described above, except that both the mutant and WT strains were incubated with particles for attachment in the same tube. Moreover, instead of enumerating the CFU of both the attached and unattached fraction, only the attached (bottom) fraction was enumerated. Specifically, after mixing and settling of the bacteria and particles, 1 ml of Tween-treated phosphate suspension from the bottom half of the settled liquid was enumerated for both total CFU attached (grown on LB agar plates with no antibiotics) and mutant CFU attached (grown on LB agar plate with corresponding antibiotics). The attachment ratio for mutant: wild-type was calculated as:

$$\text{Ratio}_{\text{with particle}} = \text{mutant}/(\text{total} - \text{mutant})$$

The same experiment was conducted with no particle added as a control (ratio$_{\text{control}}$).

This direct comparison of the bottom fraction of the settled liquid is possible only because the WT and mutant strains can be distinguished via antibiotic resistance markers.

## Cell Shearing

Approximately 10$^{10}$ cells were harvested at early stationary phase as described above, and washed twice with 10 ml $CaCO_3$ solution. The washed cells were resuspended in 10 ml $CaCO_3$ solution and subjected to blending (8 × 45 s) at 20,000 rpm on ice using a Homogenizer (Omni International., Kennesaw GA). After blending, cells were washed once using $CaCO_3$ solution and then diluted to a concentration of approximately 10$^6$ cells ml$^{-1}$.

## Proteomic Analysis

OMPs were extracted using the ReadyPrep Protein Extraction Kit (Membrane II) (Bio-Rad, Hercules, CA, USA) according to the manufacturer's instructions. The RC DC Protein assay kit (Bio-Rad, Hercules, CA, USA) was used to measure the protein concentration. Either 200 or 100 $\mu$g of protein dissolved in 125 $\mu$l of rehydration/sample buffer, as provided in the protein extraction kit, was used for the first dimension isoelectric focusing (IEF) on immobilized-pH-gradient (IPG) strips (pH 3 to 10, 7, cm, Bio-Rad, Hercules, CA, USA) at 20°C, approximately 40,000 V·h for 22 h. This first dimension separation was performed at the Protein Facility of Iowa State University.

The IPG strips were then loaded to 7 cm Any kD precast polyacrylamide gels, 8.6 × 6.7 cm (W × L, Bio-Rad, Hercules, CA, USA) for the second dimension separation. Electrophoresis was performed at 180 V until the dye front reached the bottom of the gel. Precision Plus protein standard plugs (10–250 kD,

Bio-Rad, Hercules, CA, USA) were used as molecular weight standards. The gels were stained overnight using Coomassie blue, and then destained overnight.

Images of the 2D gels were acquired using Amersham Pharmacia Biotech image scanner flatbed scanner (Amersham Pharmacia Biotech Inc, Piscataway, NJ, USA). The images were analyzed using the SameSpots (TotalLab, UK) software. This analysis included quantification of both spot size and intensity. All spot size intensity and size values were normalized relative to a reference gel, which was selected on the basis of image quality. Proteins associated with specific spots were identified by excision of the spots from the gels, in-gel trypsin digestion and mass spectrometry. Specifically, proteins were identified by matrix-assisted laser desorption ionization–time of flight tandem mass spectrometry (MALDI-TOF MS/MS) and database searching using Mascot (Matrix Science Inc, Boston, MA, USA). Image and protein analysis were performed at the Protein Facility of Iowa State University.

## OmpA Sequencing and Characterization

Open reading frames (ORF) were PCR amplified using Q5® High-Fidelity DNA Polymerase (New England Biolabs Inc, Ipswich, MA, USA), purified using QIAquick Gel Extraction Kit (QIAGEN, Germantown, MA, USA), and sequenced at the Iowa State University DNA Facility. Primers used in this study are listed in Table S1. Each ORF nucleotide sequence was converted to an amino acid sequence using the translate tool (http://web.expasy.org/translate/). All of the amino acid sequences are provided in Table S2.

Sequence logos were generated using WebLogo 3.5.0. Protein isoelectric point (pI) and molecular weight were predicted using the software PROTEIN CALCULATOR v3.4 (http://protcalc.sourceforge.net/).

## Co-occurrence Analysis

To detect the potential co-occurrence of OmpA polymorphisms, the amino acid sequences at the locations of interest of each strain were combined and converted to a binary matrix. For example, the amino acid sequence of location 25 in OmpA is N in strain 3 and P in strain 6. After the binary conversion, strain 3's OmpA amino acid sequence at location 25 would now be 1 for N and 0 for P; while strain 6's OmpA amino acid sequence at location 25 would be 0 for N and 1 for P. The Spearman's rank correlation coefficients (ρ) were calculated for all possible combinations of sequences. The significance of each sequence combination was adjusted for false discovery rate using method developed by Benjamini & Hochberg (Benjamini and Hochberg, 1995). Sequence combinations with adjusted $P \geq 0.05$ and $-0.75 < \rho < 0.75$ were eliminated. All calculations were performed using R 3.2.4 and package Hmisc.

## Gene Removal and Plasmid Construction

Recombineering via the Lambda Red-mediated gene replacement system was used to delete genes from the bacterial genome. Briefly, the DNA fragment of chloramphenicol resistance gene with homologous arms upstream and downstream of the target genes was introduced into *E. coli* cells via electroporation. With the help of the recombinase enzymes encoded by plasmid pKD46, the target gene was then replaced by the antibiotic resistance gene, as described previously (Datsenko and Wanner, 2000). PCR was conducted to confirm the success of replacement.

To construct the plasmid expressing a selected OmpA pattern, a 1.3 Kb fragment containing MG1655's *ompA* promoter and the coding region of the selected *ompA* gene was obtained by overlapping PCR and subsequently cloned into plasmid pGEN-MCS using NotI and HindIII sites.

## Statistical Analysis

One-way ANOVA and two-tailed student's *t*-test were used to analyze attachment data. Box and whisker plots were generated using BoxPlotR (shiny.chemgrid.org).

# RESULTS

As previously described (Liang et al., 2016), a library of 400 *E. coli* strains was isolated from two locations within the Squaw Creek watershed in Ames, IA during the fall of 2012 and summer of 2013 and subjected to Rep-PCR fingerprint analysis. This analysis generated genome similarity scores for each pair of isolates and a set of 78 isolates with a maximum genome similarity score of 90% was selected for further study

## Flagella Do Not Impact Corn Stover Attachment

The flagellum is a well-characterized filamentous appendage on the microbial surface which not only grants mobility in aqueous environments but also was reported to be a key genetic factor involved in biofilm formation (Pratt and Kolter, 1998; Van Houdt and Michiels, 2005). The roles of the flagella in initiating bacteria-surface attachment are (1) overcoming the repulsive force at the solid-liquid interface, and (2) providing opportunities for bacteria-surface contact (Pratt and Kolter, 1998; Gutman et al., 2013). The *fliC* gene encodes the flagellin protein, a primary subunit of the flagella filament. Each of the *fliC* deletion mutants constructed here was confirmed to be non-motile by the motility agar stab test (Figure S1).

Using the competitive attachment assay designed in this study (**Figure 1**), we found that none of these *fliC* deletion mutants showed a statistically significant ($P < 0.05$) change in attachment to corn stover (**Table 1**). It should be noted that this competitive attachment assay can only be used to directly compare strains that can be distinguished in the settled liquid. For example, here we used antibiotic resistance markers to distinguish the wild-type strain and the corresponding *fliC* deletion mutant.

These results demonstrate that while flagella may contribute to binding of cells to some types of particles, they are not involved in binding to corn stover. Thus, we sought to identify other proteins that might be involved in mediating attachment to corn stover.

## Shearing Treatment Impacts Attachment of Some Isolates

A homogenizer was used to shear and remove the flagella from the surface of a set of 18 of our 78 isolates, using a method similar to previous reports (Rosenbaum and Child,

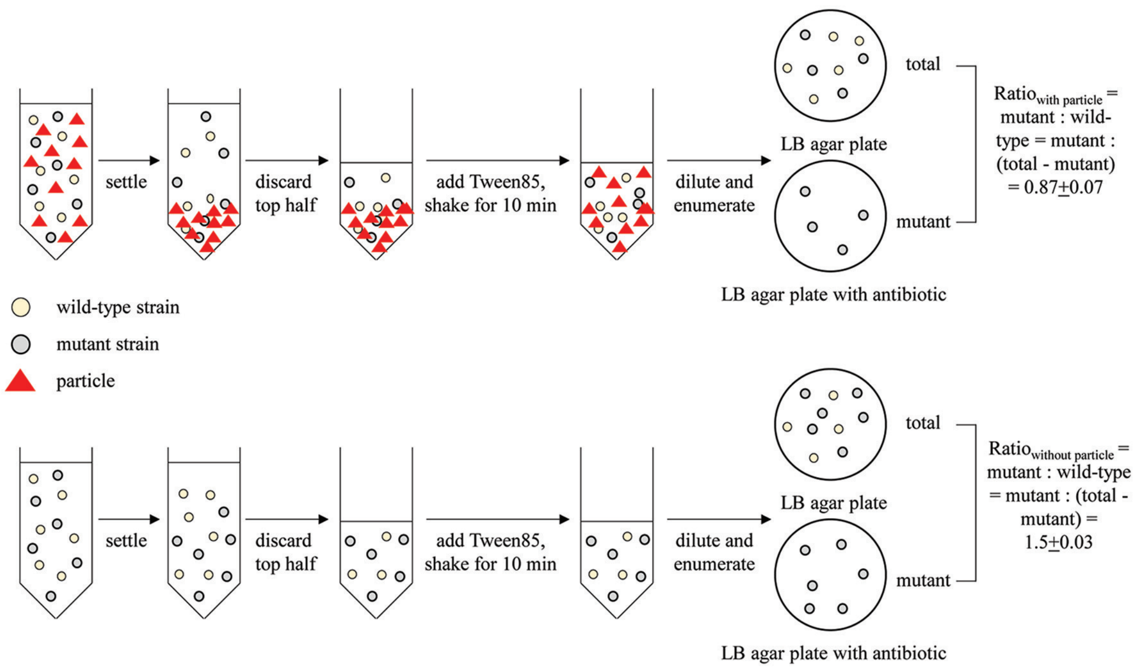


**FIGURE 1 | Schematic of the competitive attachment assay developed here, using attachment of wild type MG1655 and mutant MG1655: *ΔfliC* to corn stover as an example.** Mutant and wild type ratio was calculated as mutant: (total-mutant).

**TABLE 1 | Flagella have little impact on attachment to corn stover.**

| | Ratio of *ΔfliC* mutant to wild type | | |
|---|---|---|---|
| **Strain** | **No particle** | **Corn stover** | ***P*-value** |
| MG1655 | 0.87 ± 0.07 | 1.5 ± 0.3 | 0.11 |
| Isolate 44 | 0.93 ± 0.08 | 1.3 ± 0.3 | 0.22 |
| Isolate 117 | 1.13 ± 0.03 | 1.6 ± 0.4 | 0.25 |

*Flagellin component fliC was deleted and the corresponding mutant and wild-type were subjected to competitive attachment assay. Changes in attachment ability were obtained from the competitive attachment assay (n = 3) between the indicated ΔfliC mutant and corresponding wild type strain, with the mean value and standard error shown. If the ratio is greater than 1.0, the mutant has higher attachment ability than wild type. P-values were determined by comparing the ratios from the assays performed with and without corn stover via the Student's t-test.*

1967). These 18 isolates were selected randomly from our pool of genomically distinct isolates. Note that this shearing process is also expected to remove other surface proteins. Images from the TEM examination show that this shearing procedure was effective in removing at least some flagella from the cell surface (Figure S2). Specifically, flagella can be observed in images collected before blending, but not in the images collected after blending.

We characterized the attachment behavior of each of the 18 isolates in both the unblended and blended state (**Figure 2**). Note that this attachment assay sometimes produces negative values, meaning that there were more CFU in the top half of the settled liquid than in the bottom half, possibly due to cell buoyancy (Wan et al., 1995). Five isolates (57, 108, 117, 217, and 331) had at least a 5% decrease in attachment after shearing. This decreased attachment could possibly be attributed to removal of a surface protein or feature involved in promoting attachment. Three isolates (44, 69, and 308) had an increase of at least 5%. This increased attachment could indicate that a surface protein or feature that inhibited attachment was removed by the shearing process. The other 10 isolates had less than a 5% difference in attachment before and after shearing. This lack of change in attachment indicates that either the flagella, or other surface proteins, controlling the attachment behavior were not thoroughly removed by the blending process or that the attachment behavior of these strains is controlled by something that was not affected by the blending treatment.

The finding that the removal of surface proteins actually increased attachment in some cases was unexpected. This could possibly be due to the removal of a surface protein that had a repulsive interaction with the corn stover particles. The goal here was to identify genetic factors involved in promoting attachment to corn stover, so strains whose attachment decreased after shearing treatment were selected for further analysis.

## Proteomic Analysis Identifies OmpA as Contributing to Attachment

To investigate the surface structures that contribute to bacteria-particle attachment, we did two-dimensional (2-D) gel electrophoresis analysis for OMPs. OMP profiles of three randomly-selected strains with decreased attachment ability to corn stover after blending (57, 117, and 217) were compared to three randomly-selected strains with no change in attachment

(62, 100, and 157). This comparison identified 4 spots with potentially different expression levels between these two groups of strains (**Table 2**). The most promising spot, #5352, was present on the gels of strains 62, 100, and 157, but was not detected on the gels of strains 57, 117, and 217 (**Figure 3**). In-gel digestion and MALDI-TOF MS/MS analysis identified this spot as outer membrane protein OmpA. Similar characterization revealed that spot #5405 is also OmpA. Thus, the spots associated with OmpA were in sufficiently different locations on the gels of strains 57, 117, and 217 vs. strains 62, 100, and 157 to be identified by the image analysis software as two distinct spots. These results suggest that OmpA may contribute to the patterns of attachment observed for our strains and this shifting of the OmpA-associated spot indicates that there are variations at the molecular level among the OmpAs. This led us to sequence the *ompA* gene for each of our 78 isolates.

## Allelic Variation of OmpA

Consistent with the shifting OmpA spots in the proteome analysis, we found that there is substantial variation in the translated sequences (**Figure 4**, **Table 3A**). Specifically, polymorphisms were observed in each of the four extracellular loops, within three of the eight transmembrane segments, within the proline-rich linker and at two positions within the dimerization domain. As described below, some of these variations have been previously noted. The polymorphisms are described according to the amino acid numbering convention used by Pautsch and Schulz (Pautsch and Schulz, 1998). Note that the N-terminal domain of OmpA is generally considered to consist of residues 1–172 and the C-terminal domain of residues 189–325, with residues 173–188 being the proline-rich linker. Residues 189–276 comprise the proposed dimerization domain (Marcoux et al., 2014).

The polymorphism within loop 1 falls nearly in the middle of the loop, within the region designated by Pautsch and Schulz as "highly mobile" (Pautsch and Schulz, 1998). This residue is asparagine in nearly half (46%) of our isolates, and aspartic acid or proline in the other 15 and 39% of the isolates, respectively. Asparagine is uncharged, aspartic acid is negatively charged and proline is hydrophobic and contains a secondary amine group. Thus, the variation within loop 1 includes structurally and functionally variable amino acids.

The polymorphism within loop 2 consists of three amino acids and is also within a loop segment previously designated as "highly mobile." Slightly more than half of our isolates (51%) encode serine-valine-glutamate, 46% encode aspartate-asparagine-isoleucine and two others encode variations of this SVE pattern: one encodes serine-valine-alanine and the other encodes serine-valine-valine. Thus, 97% of our isolates present one residue with a polar uncharged side chain (S or N), one hydrophobic residue (V or I), and one negatively charged acidic residue (E or D) in this variable region of loop 2.

The third extracellular loop, which was previously concluded to not have a "highly mobile" region (Pautsch and Schulz, 1998), consists not just of amino acid changes relative to the standard OmpA sequence, but also consists of the insertion of up to five additional amino acids. The polymorphism includes the first amino acid of the subsequent transmembrane segment, with each isolate encoding either an asparagine or aspartic acid at position 114. Eight variations were observed in this position (**Figure 4**, **Table 3A**). The standard SNVYG was observed in 39.7% of our isolates, with another 9.0% encoding the two-residue variation of SN**FD**G, with bold font indicating the variant residues. Another 39.7% of the population encodes a sequence which is substantially different from the standard SNVYG. Specifically these isolates encode ANVPGGASF, with two other strains differing from this pattern at only one residue each (ANVPGGAS**Y** and ANVPG**V**ASF) and two others differing at two or three residues (**S**NVPGGAS**T** and **S**NVPGG**VST**). There is also a substantial representation (6.4%) of the sequence AHNNVTGESE.

As with loops 1 and 2, the variation within loop 4 falls within the "highly mobile" section. This amino acid varies between histidine, positively charged, for 78% of our isolates and asparagine, uncharged, for the other 22% of the isolates.

The four remaining N-terminal domain polymorphisms are within transmembrane segments (**Figure 4**). Two are within the transmembrane segments flanking loop 3, both near the periplasmic end. At position 93, 50% of isolates encode isoleucine

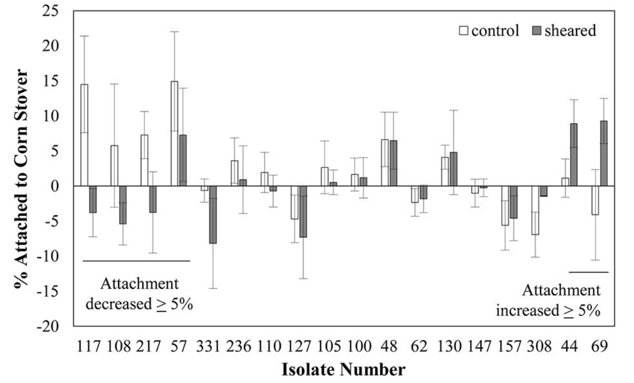


**FIGURE 2 | Shearing impacts the attachment of some *E. coli* isolates to corn stover.** Values are the average ± standard error of 3–6 replicates. Strains are ordered by the value of the change in attachment after shearing treatment.

**TABLE 2 | Spots analyzed by SameSpots from 2D gels and identified by MALDI-TOF MS during comparison of three strains with no change in attachment to corn stover after shearing treatment (62, 100, and 157) to three strains that showed decreased attachment after shearing treatment (57, 117, and 217).**

| Spot number | Hits[a] | Score[b] | pI given by SameSpots | MW (Dalton) | Protein |
|---|---|---|---|---|---|
| 5,352 | 3 | 1.38 | 6.72 | 28,161 | OmpA |
| 5,405 | 3 | 124 | 6.38 | 27,341 | OmpA |
| 5,737 | 1 | 63 | 5.8 | 20,642 | OmpA |
| 5,230 | 2 | 58 | 4.51 | 31,205 | OmpC |

*Spots 5,352, 5,405, and 5,737 are shown in **Figure 3**.*
[a] *Hits: number of matched peptides, sequence of amino acids matching the identified protein determined by MALDI-TOF-MS.*
[b] *Ions score is −10*Log(P), where P is the probability that the observed match is a random event. Individual ions scores >40 indicate identity or extensive homology ($P < 0.05$).*

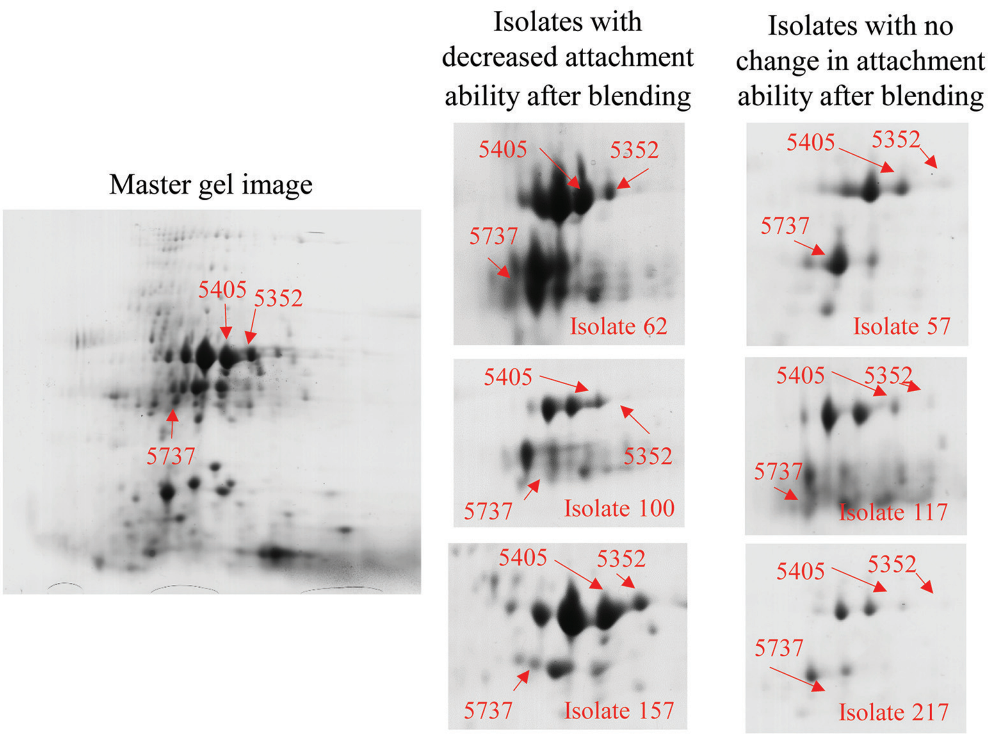


**FIGURE 3 | Outer membrane proteomic analysis showed shifting of the OmpA-associated spot between groups of strains responding differently to blending.** The photo on the left is a 2D gel selected as standard gel for SameSpots analysis. Spots were identified and selected for excision via the SameSpots software, but the images shown here are the raw images, manually labeled for clarity. The figures on the right show spots #5352, #5405, and #5737 (**Table 2**) from each of the six strains. Excised spots were subjected to protein identification through in-gel digestion, MALDI-QUAD-TOF and MS/MS.

and 50% encode valine, both of which are branched chain and hydrophobic. At position 129, 94% of isolates encode tyrosine and 6% encode tryptophan, both of which are hydrophobic and contain aromatic groups. The other two polymorphisms within the N-terminal domain are directly adjacent to extracellular loops. Position 114 encodes asparagine, polar and uncharged, in 55% of isolates and aspartate, negatively charged, in 45% of isolates. At position 161, 63% of isolates encode methionine and 37% encode leucine, both of which are hydrophobic.

Within the proline-rich linker and C-terminal domain, there are five residues at which variations were observed. Within the linker segment at position 175, three isolates encode a valine and the rest encode alanine. Valine and alanine both have hydrophobic side chains. At position 180, one isolate (strain 18) encodes a threonine, the rest encode an alanine (not shown). At position 203, 18 isolates encode a threonine, while the rest encode an asparagine. Asparagine and threonine both have polar, uncharged side chains, though asparagine is neutral and threonine is polar. At position 251, eight strains encode an alanine, while the rest of the isolates encode glycine. Glycine and alanine belong to different functional categories. At position 270, a single isolate (strain 385) encodes a serine instead of the alanine presented by the rest of the isolates (not shown). The C-terminal domain of OmpA is not as well characterized as the N-terminal domain, but these polymorphisms do fall within the dimerization region proposed between residues 189 and 276 (Marcoux et al., 2014).

Many of these variations have been previously described, mostly notably in two studies described below (Gophna et al., 2004; Power et al., 2006). To the best of our knowledge, we are the first to report the D variant at position 25 and the polymorphism at position 129. Additionally, these two previous studies only observed the two most common variants within loop 3: ANVPGGASF and SNVYG. The SNVPGGVST, AHNNVTGESE, SNFDG, ANVPGGASF and SNVPGGAST variants have not been previously described.

## OmpA Outer Loop Sequence Patterns

While some of these polymorphisms have been previously described (Gophna et al., 2004; Power et al., 2006), both of these previous reports only considered a few *E. coli* strains or only considered two possible *ompA* alleles. Here we have expanded the list of possible patterns (**Table 3A**) and considered the co-occurrence of the various polymorphisms (**Table 4**).

We have named the patterns of the outer loop polymorphisms relative to their abundance in our isolate collection. Pattern I (N, SVE, SNVYG, H) is encoded by 33.3% of our isolates and is the standard sequence previously characterized for structural analysis, such as (Pautsch and Schulz, 1998). Pattern II (P, DNI, ANVPGGASF, N) is encoded by 20.5% of our isolate collection

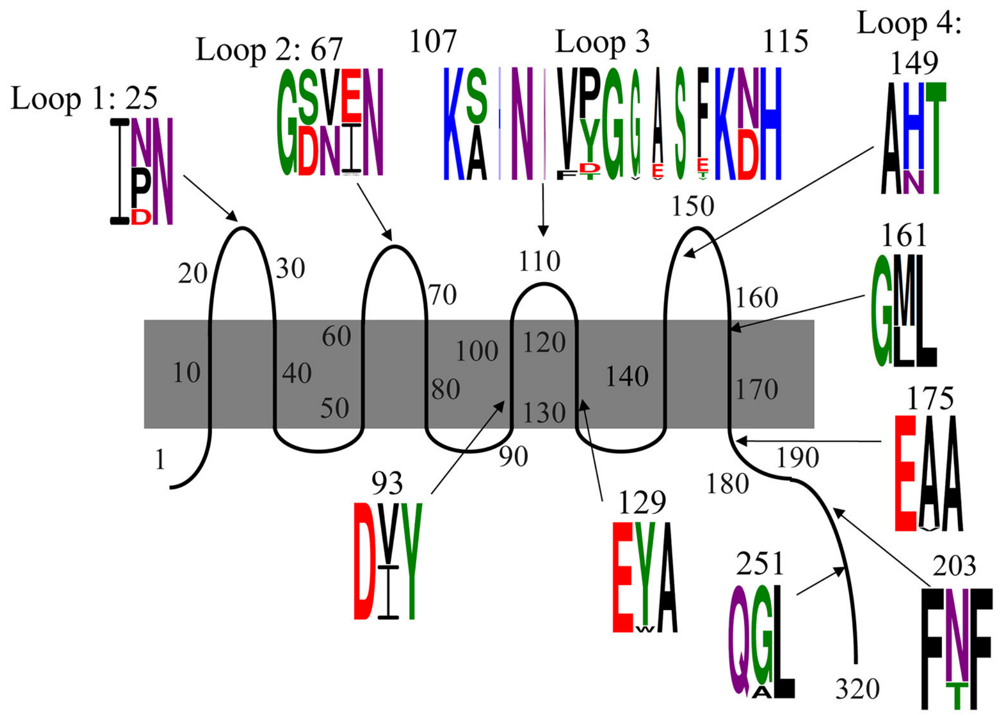


**FIGURE 4 | Polymorphisms within OmpA among our 78 *E. coli* isolates.** Residues are numbered and classified according to the description by Pautsch and Schulz (1998) and Marcoux et al. (2014). Residues 22–28, 65–68, and 147–157 were previously classified by Pautsch and Schulz (1998) as "highly mobile" regions of loops 1, 2, and 4, respectively and residues 188–276 were identified by Marcoux et al. (2014) as the dimerization domain. Two polymorphisms in the C-terminal domain were observed only in one isolate each and are not shown here, but are described in the text. Sequence logos were generated with Weblogo 3.5.0, with letter height representing probability. Colors indicate amino acid chemistry, with polar residues shown in green, neutral residues in purple, basic residues in blue, acidic residues in red and hydrophobic residues in black.

and is the only pattern that includes an asparagine at loop 4 instead of a histidine. It should be noted that these two most abundant patterns differ from each other at each of the four loops. Pattern III (P, DNI, ANVPGGASF, H) differs from pattern II only at loop 4 and is encoded by 14.1% of our strains. Pattern IV (N, SVE, SNFDG, H) differs from pattern I only at two residues within loop 3 and is expressed by 7.7% of our isolates. Pattern V (D, SVE, AHNNVTGESE, H) is encoded by 6.4% of our isolates and differs from all of the other patterns at loops 1 and 3 and also includes all of the strains that encode a tryptophan at transmembrane position 129 instead of a tyrosine (not shown).

Two other sequences are designated in **Table 3A** as patterns, even though they were only observed in one isolate each. These were selected for comparative purposes. Pattern VI (D, DNI, SNVYG, H) differs from the most abundant pattern (I) only at loops 1 and 2. Pattern VII (D, SVE, SNFDG, H) differs from pattern I only at loops 1 and 3.

Gophna et al.'s (2004) analysis identified the outer loop polymorphisms that we have designated pattern I in *E. coli* strains MG1655, O157:H7 EDL933, and K1 and pattern III in septicemic O78 strain 789 and uropathogenic strain CFT073 (Gophna et al., 2004). Power et al. compared the OmpA sequence of three *E. coli* isolates to the standard MG1655 sequence (Power et al., 2006). The human and Tasmanian devil isolates H474 and TA024 matched the MG1655 sequence, which we call pattern I. Their bird isolate, strain B194, encoded the sequence we have designated pattern II and that they designated *ompA2*. Power et al then used a PCR-based assay to screen 524 *E. coli* isolates and concluded that the *ompA2* allele was present in 43% of strains (Power et al., 2006). This PCR assay utilized a forward primer that binds within the gene segment encoding the ANVPGGA portion of loop 3 and a reverse primer that binds to a non-variable gene segment within the C-terminal domain. This assay would have given positive results for strains presenting the ANVPGGAS**F** or ANVPGGAS**Y** polymorphisms in loop 3 and thus would have given positive results for 41% of our isolate collection, consistent with the previous PCR results. However, the sequence data that we present from our 78 isolates indicates that Power's *ompA2* allele frequency calculation is incomplete, due to its lack of sensitivity to the polymorphisms in loops 1, 2, and 4.

Patterns are also observed for the polymorphisms in the linker and dimerization domain (**Table 3B**). When the polymorphisms that were observed in only one isolate each are ignored, four patterns are observed. Patterns α (A, N, G), β (A, T, G), δ (A, T, A), and γ (V, T, A) are encoded by 76.9, 12.8, 6.4, and 3.8% of isolates, respectively. The commonly characterized MG1655 OmpA encodes the α pattern.

**TABLE 3 | (A) Outer loop polymorphism patterns.**

| Pattern | 25 | 66–68 | 108–114 | 149 | Strains |
|---|---|---|---|---|---|
| | D | DNI | ANVPGGASFKD | H | 32, 333 |
| | D | DNI | SNVPGGVSTKD | H | 249 |
| VI (1.3%) | D | DNI | SNVYGKN | H | 44 |
| V (6.4%) | D | SVE | AHNNVTGESEKN | H | 62, 144, 205, 342 |
| VII (1.3%) | D | SVE | SNFDGKN | H | 127 |
| | D | SVE | SNVYGKN | H | 16, 186 |
| | N | DNI | ANVPGGASFKD | H | 110, 307 |
| | N | SVA | SNVYGKN | H | 105 |
| IV (7.7%) | N | SVE | SNFDGKN | H | 36, 111, 122, 128, 141, 185 |
| I (33.3%) | N | SVE | SNVYGKN | H | 9, 26, 28, 48, 60, 69, 84, 108, 130, 147, 157, 182, 196, 201, 204, 211, 226, 230, 270, 284, 286, 289, 292, 313, 315, 331 |
| | N | SVV | SNVYGKN | H | 3 |
| III (14.1%) | P | DNI | ANVPGGASFKD | H | 6, 53, 98, 117, 146, 158, 160, 184, 206, 281, 391 |
| II (20.5%) | P | DNI | ANVPGGASFKD | N | 8, 13, 18, 57, 83, 89, 148, 207, 217, 222, 236, 248, 257, 280, 298, 329 |
| | P | DNI | ANVPGGASFKD | H | 385 |
| | P | DNI | ANVPGVASFKD | N | 100 |
| | P | DNI | SNVPGGASTKD | H | 256 |

*Patterns are presented in alphabetical order of the polymorphisms. Patterns I–V are observed in at least 5% of our isolate collection and are named according to the observed frequency. The number in parentheses indicates the percent of our isolates encoding each pattern. Patterns VI and VII were selected for comparative purposes. Color coding indicates amino acid chemistry, as described in the legend of* ***Figure 4****.*

**TABLE 3 | (B) Linker/dimerization domain polymorphism patterns.**

| Pattern | 175 | 203 | 251 | Strains |
|---|---|---|---|---|
| α (76.9%) | A | N | G | 3, 8, 9, 13, 18, 28, 32, 44, 48, 57, 60, 62, 69, 83, 84, 89, 98, 100, 105, 108, 110, 130, 144, 147, 148, 158, 184, 196, 201, 204, 205, 206, 207, 211, 217, 222, 226, 230, 236, 248, 249, 257, 270, 280, 281, 284, 286, 289, 292, 298, 307, 308, 313, 315, 329, 331, 333, 342, 385, 391 |
| β (12.8%) | A | T | G | 6, 26, 36, 111, 117, 127, 128, 141, 146, 185 |
| δ (6.4%) | A | T | A | 122, 157, 160, 182, 256 |
| γ (3.8%) | V | T | A | 16, 53, 186 |

*Patterns are presented in alphabetical order of the polymorphisms and named according to the observed frequency. The number in parentheses indicates the percent of our isolates encoding each pattern. Color coding indicates amino acid chemistry, as described in the legend of* ***Figure 4****.*

This grouping shows that when the polymorphisms in the outer loops, linker region and dimerization domain are considered, there are 22 distinct OmpA sequence patterns, ten of which are observed in more than one isolate (**Table 3C**). Patterns (I, α) and (II, α) account for 50% of the isolates. Within these variants, the predicted protein size ranges from 37.14 to 37.71 kDa. These variants also lead to differences in the predicted pI values, from 5.90 for the five isolates in pattern (V, α) to the 6.41 predicted for the 26 total isolates in patterns (I, α), (I, β), and (I, δ)

**TABLE 3 | (C) Outer loop/linker/dimerization domain patterns and the associated molecular weight and pI predicted by Protein Calculator 3.4.**

| Pattern | MW (kDa) | pI | Strains |
|---|---|---|---|
| I, α (29.5%) | 37.20 | 6.41 | 9, 28, 48, 60, 69, 84, 108, 130, 147, 196, 201, 204, 211, 226, 230, 270, 284, 286, 289, 292, 313, 315, 331 |
| I, β (1.3%) | 37.17 | 6.41 | 26 |
| I, δ (2.6%) | 37.19 | 6.41 | 157, 182 |
| II, α (20.5%) | 37.44 | 5.97 | 8, 13, 18, 57, 83, 89, 148, 207, 217, 222, 236, 248, 257, 280, 298, 329 |
| III, α (7.7%) | 37.48 | 6.16 | 98, 158, 184, 206, 281, 391 |
| III, β (3.8%) | 37.48 | 6.16 | 6, 117, 146 |
| III, δ (1.3%) | 37.49 | 6.16 | 160 |
| III, γ (1.3%) | 37.52 | 6.16 | 53 |
| IV, β (6.4%) | 37.19 | 6.16 | 36, 111, 128, 141, 185 |
| IV, δ (1.3%) | 37.19 | 6.16 | 122 |
| V, α (6.4%) | 37.71 | 5.90 | 62, 144, 205, 308, 342 |
| VI, α (1.3%) | 37.20 | 6.16 | 44 |
| VII, β (1.3%) | 37.18 | 5.92 | 127 |
| None, α (11.5%) | 37.17 | 6.70 | 3 |
| | 37.49 | 5.92 | 32, 333 |
| | 37.48 | 5.97 | 100 |
| | 37.14 | 6.70 | 105 |
| | 37.49 | 6.16 | 110, 307 |
| | 37.49 | 5.92 | 249 |
| | 37.51 | 6.16 | 385 |
| None, δ (1.3%) | 37.45 | 6.16 | 256 |
| None, γ (2.6%) | 37.22 | 6.16 | 16, 186 |

*The number in parentheses indicates the percent of our isolates encoding the indicated pattern.*

and the 6.70 predicted for isolates 3 and 105. This variation in OmpA molecular weight and pI could explain the shifting of the OmpA-associated protein spots that led to this sequence analysis.

Certain polymorphisms were observed to significantly co-occur (**Table 4**). For example, all isolates presenting a proline within loop 1 also encode the DNI variant in loop 2. Thus, there is a positive correlation between the proline polymorphism and the DNI polymorphism and a negative correlation between the proline polymorphism and the SVE variant. The proline polymorphism at position 25 was also found to co-occur with the ANVPGGASF form of loop 3. Two statistically significant correlations were also identified for polymorphisms within loops 2 and 3, as well as one of the loop 3 variants and the transmembrane segment polymorphism at position 129 (**Table 4**).

Some of these sequence patterns may be related to overall genome similarity between isolates. Our rep-PCR analysis yielded a genome similarity score for each pair of isolates (Table S3) (Liang et al., 2016). Due to our selection criteria for the 78 isolates characterized in this study, the maximum possible genome similarity score between any two isolates is 90%. Observed genome similarity scores ranged from 0 to 89.14%, with a mean value of 50% and standard deviation of 17%. Five OmpA patterns were each observed in at least five percent of our

**TABLE 4 | Statistically significant co-occurrence of amino acid variations.**

| Variant pair | Adjusted *P*-value | *ρ* |
|---|---|---|
| **LOOPS 1 AND 2** | | |
| N, DNI | $6.5 \times 10^{-14}$ | −0.754 |
| P, DNI | 0 | 0.854 |
| P, SVE | 0 | −0.811 |
| **LOOPS 1 AND 3** | | |
| P, ANVPGGASFKD | 0 | 0.812 |
| **LOOPS 2 AND 3** | | |
| SVE, ANVPGGASFKD | 0 | −0.833 |
| DNI, ANVPGVASFKD | 0 | 0.877 |
| **LOOP 3 AND POSITION 129** | | |
| AHNNVTGESEKN, W | 0 | 1.00 |
| AHNNVTGESEKN, Y | 0 | −1.00 |

*Data is shown only for polymorphism pairs that have an adjusted $P \leq 0.05$ and Spearman's Correlation Coefficient (ρ) with an absolute value > 0.75.*

isolates: (I, α), (II, α), (III, α), (IV, β), and (V, α). The average genome similarity scores for these groups are 47, 59, 65, 65, and 52%, respectively, with a one-way ANOVA *P*-value of $9.3 \times 10^{-11}$. Direct comparison between groups with a two-tailed *t*-test identified significant differences for pattern (I, α) vs. patterns (II, α) ($P = 2.1 \times 10^{-9}$), (III, α) ($P = 2.0 \times 10^{-4}$), and (IV, β) ($P = 0.0017$). Pattern (V, α) differed significantly from patterns (III, α) ($P = 0.0098$) and (IV, β) ($P = 0.030$). The fact that strains encoding patterns (III, α) and (IV, β) both had significantly higher genome similarity scores than those encoding patterns (I, α) and (V, α) is consistent with patterns (III, α) and (IV, β) being newly-emerged relative to patterns (I, α) and (V, α).

## OmpA Allelic Variation Is Associated with Attachment to Corn Stover

Our sequencing of OmpA was motivated by the differential response of some strains to shearing treatment. The three strains selected for proteomic analysis due to their decreased attachment to corn stover after blending (57, 117, and 217) all have the same polymorphisms within loops 1 (P), 2 (DNI), and 3 (ANVPGGASFKD). In contrast, the two strains that showed increased attachment to corn stover after blending (44 and 69) both encode the SNVYGKN variant at loop 3 and the H polymorphism within loop 4. Of the nine strains that showed less than 5% change in corn stover attachment after shearing (48, 62, 100, 105, 110, 127, 130, 147, and 157), only one (100) presents the P variation at loop 1, only two (100 and 110) present the DNI variation at loop 2, and only one (110) presents the ANVPGGASFKD variant at loop 3 associated with decreased attachment after shearing. None of these nine strains present the (P, DNI, ANVPGGASFKD) combination observed in the isolates that showed decreased attachment after shearing. Four of these nine strains (105, 130, 147, 157) do encode the SNVYGKN at loop 3 and H at loop 4 associated with increased attachment after shearing. Thus, attachment to corn stover, especially decreased attachment after shearing treatment, appears to be related to the OmpA outer loop sequence. No such trends were apparent for the linker and dimerization domain polymorphisms.

In order to quantify the association of the OmpA polymorphisms and attachment of the isolates to corn stover, we performed the corn stover attachment assay for each of the isolates not already characterized (Table S4). Values ranged from −17 to 29%, with an average of 6.4%. As noted above, negative values for the attachment assay stem from higher cell counts in the top half of the settled liquid relative to the bottom half, possibly due to cell buoyancy (Wan et al., 1995).

Statistical analysis was performed for each sequence variation or pattern that was observed in at least five isolates (**Figure 5**). At locations where only two variants were present in at least five isolates, the Student's *t*-test was used. At locations where more than two variants were observed in at least five isolates, one-way ANOVA was used to compare the mean values, and then the Student's *t*-test was used to directly compare groups. Patterns VI and VII were not included in this analysis since they are each present in only one isolate each. Of the individual polymorphisms, significant trends were observed only for loop 3, with a one-way ANOVA *P*-value of 0.019. Direct comparison showed that strains encoding SNFDGKN had a significantly lower attachment than strains encoding ANVPGGASFKD ($P = 0.0032$) and the standard loop 3 sequence SNVYGKN ($P = 0.011$) (**Figure 5**).

When isolates were analyzed according to the outer loop patterns defined in **Table 3A**, significant differences were observed, with a *P*-value of 0.045 by one-way ANOVA (**Figure 5**). Isolates encoding pattern I significantly differed from pattern IV ($P = 0.030$), though these two patterns differ only by two residues within loop 3 (SN**VY**GKN vs. SN**FD**GKN). Also, pattern III differed from pattern IV ($P = 0.0087$), these patterns differ at loops 1, 2, and 3, but not loop 4. It is interesting to note that the two most abundant patterns, I and II, do not significantly differ ($P = 0.66$), despite encoding different sequence variants at each of the four extracellular loops.

When the linker and dimerization domain polymorphisms were included with the outer loop patterns, the one-way ANOVA *P*-value was 0.012. Both of the significant differences attributed to outer loop polymorphisms were still observed (**Figure 5**). Specifically, strains encoding the (I, α) pattern significantly differed from those encoding (IV, β) ($P = 0.0097$) and strains encoding pattern (III, α) significantly differed from those encoding (IV, β) ($P = 0.0032$). This analysis also identified significant differences between patterns (II, α) and (IV, β) ($P = 0.026$) and (III, α) and (V, α) ($P = 0.025$). Thus, naturally-occurring sequence patterns in OmpA are associated with significant differences in attachment to corn stover.

## OmpA Allelic Variations Impact Attachment in a Single Genetic Background

Our results thus far suggest that allelic variation in the sequence of OmpA contributes to differences in attachment to corn stover. However, these results simply show an association and not a direct cause. This associative effect could involve a variety of other cell features. Therefore, we further explored the role of OmpA and variations in its sequence within a single genetic background. We selected the standard K-12 *E. coli*

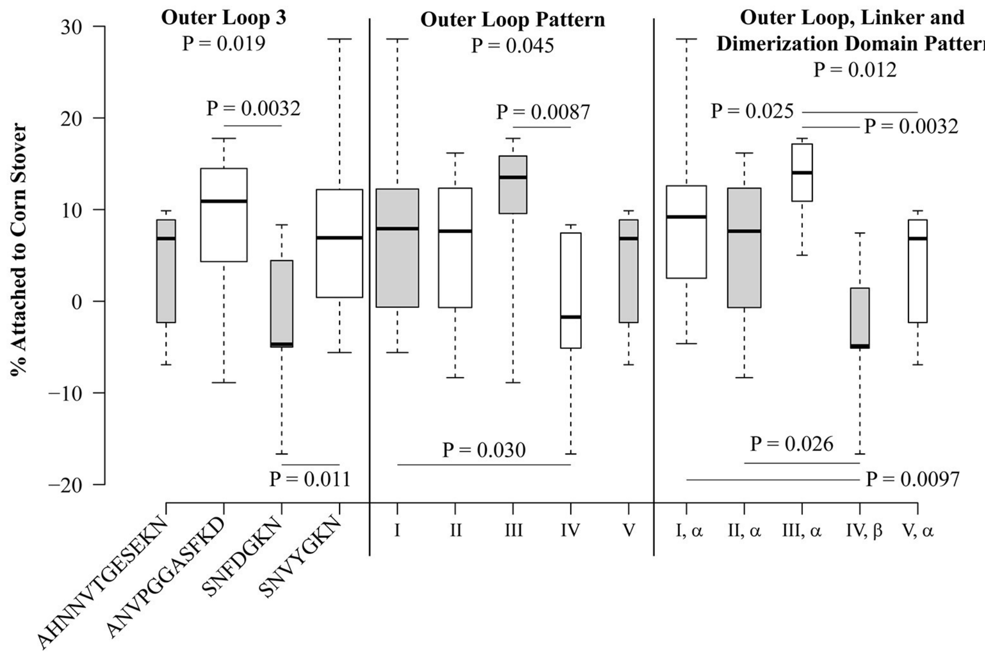


**FIGURE 5 | Polymorphisms within OmpA are significantly associated with attachment of *E. coli* isolates to corn stover.** Box plots were generated using BoxPlotR. The whiskers indicate the minimum and maximum values. The bottom of the box is the 1st quartile and the top of the box indicates the 3rd quartile. The dark line within the box is the median value. Box width is scaled according to the number of strains in each group. Shading of the boxes is for improved readability and does not convey meaning. Group-wide *P*-values were determined using one-way ANOVA, *P*-values between individual sequences and patterns were determined using the Student's *t*-test.

strain MG1655, which encodes the (I, α) version, for these studies. Specifically, the effect of these sequence variations on the attachment behavior of MG1655 was explored by expressing seven distinct OmpA versions from a plasmid in the MG1655 *ΔompA* strain (**Table 5**).

These results confirm some of the significant associations observed in the isolate studies. For example, isolates encoding pattern (I, α) had a significantly higher attachment to corn stover than strains encoding pattern (IV, β), $P = 0.0097$ (**Figure 5**). When these two versions of OmpA were expressed in the same genetic background, the attachment values were significantly different ($P = 0.0058$), and showed same trend. This shows that the difference in corn stover attachment observed in the isolate strains can be at least partially attributed to the variation in OmpA sequence.

However, not all of the significant associations observed for the isolates were observed in the MG1655 studies. For example, pattern (II, α) had a significantly higher mean attachment value than pattern (IV, β) in the isolates ($P = 0.026$), but no significant difference was observed when these two sequences were expressed in MG1655 ($P = 0.1$).

Other significant differences were detected in this comparison that were not detected in the isolate studies. Most notably, patterns (V, α), (VI, α), and (VII, β) each had a significantly higher attachment than patterns (I, α), (III, γ), and (IV, β) (**Table 5**). Patterns V, VI, and VII each encode an aspartic acid in loop 1, while patterns I and IV encode an asparagine and pattern III encodes a proline. Note that patterns VI and VII were not analyzed in the isolate studies, since they were observed in less than five isolates.

To summarize, these results demonstrate that when expressed in the same genetic background, naturally-occurring sequence variations within OmpA result in significant differences in attachment to corn stover. Note that previous work has shown that overexpression (Ma and Wood, 2009) and deletion of *ompA* (Wang, 2002) can both lead to low viability. Consistent with these previous reports, we were unable to characterize attachment for *ompA* deletion mutants due to low survival of the attachment assay (*data not shown*).

## DISCUSSION

The goal of this project was to increase understanding of the genetic factors that drive attachment of *E. coli* to a representative biotic environmental particle under environmentally relevant conditions. Here we have demonstrated that while flagella do not play a clear role in this attachment process, variations in the amino acid sequence of OmpA appear to play a significant role in attachment of *E. coli* to corn stover.

OmpA is one of the most abundant outer membrane proteins for Gram-negative bacteria, with expression of 2–3 $\times$ $10^5$ molecules per cell (Movva et al., 1980). As a transmembrane protein embedded in the outer membrane, it crosses the membrane eight times, resulting in four large and hydrophilic extracellular loops and four small periplasmic turns (Vogel and Jähnig, 1986; Pautsch and Schulz, 1998, 2000; Arora et al.,

**TABLE 5 | Expression of distinct OmpA variants in the same genetic background significantly impacts attachment to corn stover.**

| Template Strain (polymorphism pattern) | MG1655 (I, α) | 57 (II, α) | 53 (III, γ) | 111 (IV, β) | 62 (V, α) | 44 (VI, α) | 127 (VII, β) |
|---|---|---|---|---|---|---|---|
| % Attached, average ± SE ($n$ = 3) | 0 ± 0 | −8 ±11 | −24 ± 2 | −35 ± 7 | 14 ± 4 | 11 ± 1 | 18 ± 4 |
| ***P*-VALUES, STUDENT'S *T*-TEST** | | | | | | | |
| MG1655 (I, α) | | | 0.00049 | 0.0058 | 0.020 | 0.0011 | 0.013 |
| 57 (II, α) | | | | | | | |
| 53 (III, γ) | 0.00049 | | | | 0.00098 | 0.00019 | 0.00099 |
| 111 (IV, β) | 0.0058 | | | | 0.0028 | 0.0022 | 0.0024 |
| 62 (V, α) | 0.020 | | 0.00098 | 0.0028 | | | |
| 44 (VI, α) | 0.0011 | | 0.00019 | 0.0022 | | | |
| 127 (VII, β) | 0.013 | | 0.00099 | 0.0024 | | | |
| **OmpA POLYMORPHISMS** | | | | | | | |
| Loop 1 | N | P | P | N | D | D | D |
| Loop 2 | SVE | DNI | DNI | SVE | SVE | DNI | SVE |
| 93 | I | I | I | V | V | V | V |
| Loop 3 | SNVYGKN | ANVPGGASFKD | ANVPGGASFKD | SNFDGKN | AHNNVTGESEKN | SNVYGKN | SNFDGKN |
| 129 | Y | Y | Y | Y | W | Y | Y |
| Loop 4 | H | N | H | H | H | H | H |
| 161 | M | L | M | M | L | L | M |
| 175 | A | A | V | A | A | A | A |
| 203 | N | N | T | T | N | N | T |
| 251 | G | G | A | G | G | G | G |

*OmpA was cloned from the indicated template strain and expressed from the pGEN-MCS expression vector in MG1655 ΔompA. The one-way ANOVA P-value was 3.9 × $10^{-5}$. Amino acids are color-coded according to the convention described in the legend of* ***Figure 4****. P values are shown only when the value was less than 0.05.*

2001; Smith et al., 2007). OmpA can act as a porin for uptake of small molecules (Pautsch and Schulz, 1998; Arora et al., 2000), as a receptor for bacteriophage and colicin (Foulds and Barrett, 1973; Morona et al., 1984), and also as a structural protein to maintain the integrity of the outer membrane (Sonntag et al., 1978; Koebnik, 1995). It has also been reported to be an important target for immune response, a contributing factor to the pathogenicity of *E. coli* (Weiser and Gotschlich, 1991; Prasadarao et al., 1996; Belaaouaj et al., 2000; Wang, 2002; Godefroy et al., 2003; Jeannin et al., 2003), and a critical component of biofilm formation (Danese et al., 2000; Orme et al., 2006; Rivas et al., 2008).

Sequence variation for OmpA has been previously noted among bacterial genera. Wang's (2002) comparison of *E. coli* K1, *Salmonella typhimurium*, *Shigella flexneri*, *Enterobacter aerogenes,* and *Klebsiella pneumonia* concluded that the amino acid sequence of the extracellular loops is greatly variable among genera (Wang, 2002). Weiss et al. (2008) comparison of OmpA across nine species also noted the variability among the extracellular loops. Some of these variations were shown to impact survival in acid, high salt and serum (Wang, 2002) and pathogenesis in tsetse fly (Weiss et al., 2008) but their role in attachment was not described.

OmpA sequence variation has also been reported within *E. coli*. Gophna et al. (2004) and Power et al. (2006) each observed many of the individual polymorphisms reported here. These previous studies linked OmpA polymorphisms to resistance to neutrophil elastase (Gophna et al., 2004) and bacteriophage (Gophna et al., 2004), but did not consider attachment. Our consideration of 78 strains enabled us to identify low-frequency sequence variations that have not been previously reported. Our larger dataset and consideration of the entire *ompA* sequence also allowed us to identify patterns and significant co-occurrences among these polymorphisms. The fact that 30% of the isolates in our collection encode outer loop sequences distinct from those described by these two previous studies emphasizes the contribution of this dataset to capturing the variability within *E. coli* OmpA.

When the polymorphisms in the transmembrane segments are not considered, we observed 22 distinct OmpA sequences (**Table 3C**). Fifty percent of our 78 isolates encode either pattern (I, α) or (II, α). When only the outer loop polymorphisms are considered, there are five patterns (I, II, III, IV, and V) that are observed in at least five isolates each, with the two most abundant patterns differing at each of the four extracellular loops. Significant co-occurrences were observed for polymorphisms in loops 1, 2, and 3, but not loop 4 (**Table 4**). This variation in OmpA is consistent with a genome-wide analysis of *Salmonella enterica* that concluded that surface-exposed molecules display a high degree of allelic variation, possibly contributing to pathoadaptation (Yue et al., 2015).

The OmpA outer loop sequence variations showed a significant association with attachment to corn stover among our isolate collection (**Figure 5**). Statistically significant differences were detected for the loop 3 sequences SNVYKGN and SNFDGKN at each level of analysis: when grouping only by the loop 3 sequence ($P$ = 0.011), when grouping only by the

outer loop pattern (I vs. IV) ($P = 0.0087$), when grouping by the combined outer loop/linker/dimerization domain pattern (I, α vs. IV, β) ($P = 0.0097$) and when expressed in the same genetic background ($P = 0.0058$). However, OmpA sequence variation obviously does not explain all of the observed variation in corn stover attachment. Other outer membrane proteins and structures are likely to contribute to the variation that we observed between isolates expressing the same OmpA sequence.

Krishnan et al. (2014) previously demonstrated through targeted mutagenesis that specific interactions can occur between loops 2 and 3 of OmpA to glycan groups on human cell receptors (Krishnan et al., 2014). Thus, it is possible that the attachment of *E. coli* cells to corn stover is also mediated by chemical bond formation between OmpA's extracellular loops and the glycan groups on the surface of the corn stover particle. Therefore, the changes in attachment associated with expression of different versions of OmpA can possibly be attributed to the formation of stronger or additional chemical bonds. A 2015 study of *E. coli* O157:H7 EDL933 concluded that deletion of *ompA* significantly decreases attachment to glass and to spinach (Nagy et al., 2015), though it was proposed that the decreased attachment may be due to problems with membrane structure and cell physiology.

It is possible that some of the differences observed in the MG1655 studies, particularly those that were not observed in the isolates (**Table 5**), are due to interactions of OmpA with other membrane components. For example, a 1980 publication cited difficulty in functional expression of *ompA* from clinical isolates in *E. coli* K12 (Beher et al., 1980). The authors concluded that successful cell surface expression of OmpA requires interaction between OmpA and lipopolysaccharides, where these lipopolysaccharides can vary between strains (Beher et al., 1980), consistent with the conclusions of other researchers (Schweizer et al., 1978). The lack of consistency between the associative studies and expression in MG1655 could also indicate that the differences in attachment behavior are due to features other than OmpA.

Previous reports of the role of flagella in bacterial attachment to surfaces have been mixed (Rivas et al., 2006; Gutman et al., 2013), consistent with our own mixed results. Beyond functioning as the appendage generating motility, flagella also contribute to surface hydrophobicity and net surface charge and associate with surfaces through both steric and electrostatic interactions (De Kerchove and Elimelech, 2008). Therefore, our observation that different *E. coli* isolates have a differential response to blending in terms of corn stover attachment (**Figure 2**) can possibly be attributed to other structures revealed on the cell surface after blending. Alternatively, the loss of flagella-mediated tumbling may have allowed increased interaction time between the cells and particle surfaces so that attachment was more likely. EPS have also been previously reported to play a role in bacterial attachment (Matthysse et al., 2008; Macarisin et al., 2012). However, our characterization of MG1655 deletion mutants of *wcaD*, *bcsA,* and *pgaC* suggested that these three EPS-associated genes play either no role or only a minor role in attachment to corn stover (*data not shown*).

A variety of methods have previously been used to test the attachment of bacteria to abiotic surfaces, such as deposition rate during flow through a packed column (Foppen and Schijven, 2005; Levy et al., 2007), flow cytometry (Liang et al., 2014), and settling, centrifugation, and filtration (Schillinger and Gannon, 1985; Soupir et al., 2008; Krometis et al., 2009; Liu et al., 2011). Here we developed a method of directly comparing attachment behavior of two strains (**Figure 1**). This method is a modification of the previously-described settling method to investigate the role of a specific genetic factor on attachment and should be of use in a variety of studies in which strains can be distinguished, such as through the use of antibiotic resistance markers.

## AUTHOR CONTRIBUTIONS

CL, XL, and MS collected strains. CL performed rep-PCR; generated the strains and performed the sequence analysis and experiments for the data presented in **Tables 1–3**, **5**, and **Figures 2–5**, Figures S1, S2 and wrote the manuscript draft. XL generated the isolate attachment data and developed the protocols for the attachment assays. FY performed the co-occurrence analysis. MS, AH, and MT contributed to experimental design, data analysis and manuscript writing. LJ contributed to experimental design, data analysis and finalized the manuscript.

## FUNDING

Funding for this work was provided by the National Science Foundation grant CBET-1236510. The funders had no role in study design, data collection and interpretation, or the decision to submit the work for publication.

## ACKNOWLEDGMENTS

We thank Yingxi Chen, Le Zhao, Fuyuan Jing, and Wan Sun for assistance in attachment assay. Thanks to Dr. Jean-Phillipe Tessonnier from the Department of Chemical & Biological Engineering, ISU, for providing the equipment of Zetasizer Nano-ZS. Thanks to Tracey Stewart from Microscopy and NanoImaging Facility for Transmission Electron Microscopy and Joel Nott from Protein Facility for assistance in proteomics analysis. We also thank Thaddeus Stanton, Catherine Logue, and Larry Halverson for helpful conversations.

## SUPPLEMENTARY MATERIAL

The Supplementary Material for this article can be found online at: http://journal.frontiersin.org/article/10.3389/fmicb.2017.00708/full#supplementary-material

## REFERENCES


Adler, P. R., Rau, B. M., and Roth, G. W. (2015). Sustainability of corn stover harvest strategies in Pennsylvania. *BioEnergy Res.* 8, 1310–1320. doi: 10.1007/s12155-015-9593-2

Agladze, K., Wang, X., and Romeo, T. (2005). Spatial periodicity of *Escherichia coli* K-12 biofilm microstructure initiates during a reversible, polar attachment phase of development and requires the polysaccharide adhesin PGA. *J. Bacteriol.* 187, 8237–8246. doi: 10.1128/JB.187.24.8237-8246.2005

Arora, A., Abildgaard, F., Bushweller, J. H., and Tamm, L. K. (2001). Structure of outer membrane protein A transmembrane domain by NMR spectroscopy. *Nat. Struct. Biol.* 8, 334–338. doi: 10.1038/86214

Arora, A., Rinehart, D., Szabo, G., and Tamm, L. K. (2000). Refolded outer membrane protein A of *Escherichia coli* forms ion channels with two conductance states in planar lipid bilayers. *J. Biol. Chem.* 275, 1594–1600. doi: 10.1074/jbc.275.3.1594

Bai, S., and Lung, W. S. (2005). Modeling sediment impact on the transport of fecal bacteria. *Water Res.* 39, 5232–5240. doi: 10.1016/j.watres.2005.10.013

Beher, M., Pugsley, A., and Schnaitman, C. (1980). Correlation between the expression of an *Escherhichia coli* cell surface protein and the ability of the protein to bind to lipopolysaccharide. *J. Bacteriol.* 143, 403–410.

Belaaouaj, A., Kim, K. S., and Shapiro, S. D. (2000). Degradation of outer membrane protein A in *Escherichia coli* killing by neutrophil elastase. *Science* 289, 1185–1188. doi: 10.1126/science.289.5482.1185

Beloin, C., Valle, J., Latour-Lambert, P., Faure, P., Kzreminski, M., Balestrino, D., et al. (2004). Global impact of mature biofilm lifestyle on *Escherichia coli* K-12 gene expression. *Mol. Microbiol.* 51, 659–674. doi: 10.1046/j.1365-2958.2003.03865.x

Benjamini, Y., and Hochberg, Y. (1995). Controlling the false discovery rate - a practical and powerful approach to multiple testing. *J. R. Stat. Soc. Ser. B. Methodol.* 57, 289–300.

Bolster, C. H., Haznedaroglu, B. Z., and Walker, S. L. (2009). Diversity in cell properties and transport behavior among 12 different environmental *Escherichia coli* isolates. *J. Environ. Qual.* 38, 465–472. doi: 10.2134/jeq2008.0137

Danese, P. N., Pratt, L. A., Dove, S. L., and Kolter, R. (2000). The outer membrane protein, antigen 43, mediates cell-to-cell interactions within *Escherichia coli* biofilms. *Mol. Microbiol.* 37, 424–432. doi: 10.1046/j.1365-2958.2000.02008.x

Datsenko, K. A., and Wanner, B. L. (2000). One-step inactivation of chromosomal genes in *Escherichia coli* K-12 using PCR products. *Proc. Natl. Acad. Sci. U.S.A.* 97, 6640–6645. doi: 10.1073/pnas.120163297

Davies, C. M., and Bavor, H. J. (2000). The fate of stormwater-associated bacteria in constructed wetland and water pollution control pond systems. *J. Appl. Microbiol.* 89, 349–360. doi: 10.1046/j.1365-2672.2000.01118.x

Donlan R. M. (2002). Biofilms: microbial life on surfaces. *Emerg. Infect. Dis. J.* 8, 881. doi: 10.3201/eid0809.020063

De Kerchove, A. J., and Elimelech, M. (2008). Calcium and magnesium cations enhance the adhesion of motile and nonmotile *Pseadomonas aeruginosa* on alginate films. *Langmuir* 24, 3392–3399. doi: 10.1021/la7036229

Foppen, J. W., and Schijven, J. F. (2005). Transport of *E. coli* in columns of geochemically heterogeneous sediment. *Water Res.* 39, 3082–3088. doi: 10.1016/j.watres.2005.05.023

Foulds, J., and Barrett, C. (1973). Characterization of *Escherichia coli* mutants tolerant to bacteriocin JF246: two new classes of tolerant mutants. *J. Bacteriol.* 116, 885–892.

Godefroy, S., Corvaia, N., Schmitt, D., Aubry, J. P., Bonnefoy, J. Y., Jeannin, P., et al. (2003). Outer membrane protein A (OmpA) activates human epidermal Langerhans cells. *Eur. J. Cell Biol.* 82, 193–200. doi: 10.1078/0171-9335-00303

Golecha, R., and Gan, J. B. (2016). Effects of corn stover year-to-year supply variability and market structure on biomass utilization and cost. *Renewable Sustain. Energy Rev.* 57, 34–44. doi: 10.1016/j.rser.2015.12.075

Gophna, U., Ideses, D., Rosen, R., Grundland, A., and Ron, E. Z. (2004). OmpA of a septicemic *Escherichia coli* O78–secretion and convergent evolution. *Int. J. Med. Microbiol.* 294, 373–381. doi: 10.1016/j.ijmm.2004.08.004

Greene, R. A., Blum, E. F., DeCoro, C. T., Fairchild, R. B., Kaplan, M. T., Landau, J. L., et al. (1951). Rapid methods for the detection of motility. *J. Bacteriol.* 62, 347–347.

Gutman, J., Walker, S. L., Freger, V., and Herzberg, M. (2013). Bacterial attachment and viscoelasticity: physicochemical and motility effects analyzed using quartz crystal microbalance with dissipation (QCM-D). *Environ. Sci. Technol.* 47, 398–404. doi: 10.1021/es303394w

Hare, J. M., and McDonough, K. A. (1999). High-frequency RecA-dependent and -independent mechanisms of Congo red binding mutations in *Yersinia pestis*. *J. Bacteriol.* 181, 4896–4904.

Hori, K., and Matsumoto, S. (2010). Bacterial adhesion: from mechanism to control. *Biochem. Eng. J.* 48, 424–434. doi: 10.1016/j.bej.2009.11.014

Jackson, D. W., Suzuki, K., Oakford, L., Simecka, J. W., Hart, M. E., and Romeo, T. (2002). Biofilm formation and dispersal under the influence of the global regulator CsrA of *Escherichia coli*. *J. Bacteriol.* 184, 290–301. doi: 10.1128/JB.184.1.290-301.2002

Jeannin, P., Magistrelli, G., Herbault, N., Goetsch, L., Godefroy, S., Charbonnier, P., et al. (2003). Outer membrane protein A renders dendritic cells and macrophages responsive to CCL21 and triggers dendritic cell migration to secondary lymphoid organs. *Eur. J. Immunol.* 33, 326–333. doi: 10.1002/immu.200310006

Koebnik, R. (1995). Proposal for a peptidoglycan-associating alpha-helical motif in the C-terminal regions of some bacterial cell-surface proteins. *Mol. Microbiol.* 16, 1269–1270. doi: 10.1111/j.1365-2958.1995.tb02348.x

Krishnan, S., Liu, F., Abrol, R., Hodges, J., Goddard, W. A., and Prasadarao, N. V. (2014). The interaction of N-glycans in Fc gamma receptor I alpha-chain with *Escherichia coli* K1 outer membrane protein a for entry into macrophages experimental and computational analysis. *J. Biol. Chem.* 289, 30937–30949. doi: 10.1074/jbc.M114.599407

Krometis, L. A., Dillaha, T. A., Love, N. G., and Mostaghimi, S. (2009). Evaluation of a filtration/dispersion method for enumeration of particle-associated *Escherichia coli*. *J. Environ. Qual.* 38, 980–986. doi: 10.2134/jeq2007.0037

Kumar, R., Mago, G., Balan, V., and Wyman, C. E. (2009). Physical and chemical characterizations of corn stover and poplar solids resulting from leading pretreatment technologies. *Bioresour. Technol.* 100, 3948–3962. doi: 10.1016/j.biortech.2009.01.075

Levy, J., Sun, K., Findlay, R. H., Farruggia, F. T., Porter, J., Mumy, K. L., et al. (2007). Transport of *Escherichia coli* bacteria through laboratory columns of glacial-outwash sediments: estimating model parameter values based on sediment characteristics. *J. Contam. Hydrol.* 89, 71–106. doi: 10.1016/j.jconhyd.2006.08.006

Liang, X., Liao, C., Thompson, M. L., Soupir, M. L., Jarboe, L. R., and Dixon, P. M. (2016). *E. coli* surface properties differ between stream water and sediment environments. *Front. Microbiol.* 7:1732. doi: 10.3389/fmicb.2016.01732

Liang, X., Soupir, M. L., Rigby, S., Jarboe, L. R., and Zhang, W. (2014). Flow cytometry is a promising and rapid method for differentiating between freely suspended *Escherichia coli* and *E. coli* attached to clay particles. *J Appl. Microbiol.* 117, 1730–1739. doi: 10.1111/jam.12660

Liao, C., Liang, X., Soupir, M. L., and Jarboe, L. R. (2015). Cellular, particle and environmental parameters influencing attachment in surface waters: a review. *J. Appl. Microbiol.* 119, 315–330. doi: 10.1111/jam.12860

Liu, P., Soupir, M. L., Zwonitzer, M., Huss, B., and Jarboe, L. R. (2011). Association of antibiotic resistance in agricultural *Escherichia coli* isolates with attachment to quartz. *Appl. Environ. Microbiol.* 77, 6945–6953. doi: 10.1128/AEM.00717-11

Ma, Q., and Wood, T. K. (2009). OmpA influences *Escherichia coli* biofilm formation by repressing cellulose production through the CpxRA two-component system. *Environ. Microbiol.* 11, 2735–2746. doi: 10.1111/j.1462-2920.2009.02000.x

Macarisin, D., Patel, J., Bauchan, G., Giron, J. A., and Sharma, V. K. (2012). Role of curli and cellulose expression in adherence of *Escherichia coli* O157:H7 to spinach leaves. *Foodborne Pathog. Dis.* 9, 160–167. doi: 10.1089/fpd.2011.1020

Marcoux, J., Politis, A., Rinehart, D., Marshall, D. P., Wallace, M. I., Tamm, L. K., et al. (2014). Mass spectrometry defines the C-terminal dimerization domain and enables modeling of the structure of full-length OmpA. *Structure* 22, 781–790. doi: 10.1016/j.str.2014.03.004

Matthysse, A. G., Deora, R., Mishra, M., and Torres, A. G. (2008). Polysaccharides cellulose, poly-beta-1,6-n-acetyl-D-glucosamine, and colanic acid are required for optimal binding of *Escherichia coli* O157:H7 strains to alfalfa sprouts and K-12 strains to plastic but not for binding to epithelial cells. *Appl. Environ. Microbiol.* 74, 2384–2390. doi: 10.1128/AEM.01854-07

Morona, R., Klose, M., and Henning, U. (1984). *Escherichia coli* K-12 Outer-Membrane Protein (OmpA) as a bacteriophage receptor - analysis of mutant-genes expressing altered proteins. *J. Bacteriol.* 159, 570–578.

Movva, N. R., Nakamura, K., and Inouye, M. (1980). Regulatory region of the gene for the ompA protein, a major outer membrane protein of *Escherichia coli*. *Proc. Natl. Acad. Sci. U.S.A.* 77, 3845–3849. doi: 10.1073/pnas.77.7.3845

Murray, C. J., Ortblad, K. F., Guinovart, C., Lim, S. S., Wolock, T. M., Roberts, D. A., et al. (2014). Global, regional, and national incidence and mortality for HIV, tuberculosis, and malaria during 1990-2013: a systematic analysis for the Global Burden of Disease Study 2013. *Lancet* 384, 1005–1070. doi: 10.1016/S0140-6736(14)60844-8

Nagy, A., Mowery, J., Bauchan, G. R., Wang, L., Nichols-Russell, L., and Nou, X. (2015). Role of extracellular structures of *Escherichia coli* O157:H7 in initial attachment to biotic and abiotic surfaces. *Appl. Environ. Microbiol.* 81, 4720–4727. doi: 10.1128/AEM.00215-15

Orme, R., Douglas, C. W., Rimmer, S., and Webb, M. (2006). Proteomic analysis of *Escherichia coli* biofilms reveals the overexpression of the outer membrane protein OmpA. *Proteomics* 6, 4269–4277. doi: 10.1002/pmic.200600193

Otto, K., and Hermansson, M. (2004). Inactivation of ompX Causes Increased Interactions of Type 1 Fimbriated *Escherichia coli* with Abiotic Surfaces. *J. Bacteriol.* 186, 226–234. doi: 10.1128/JB.186.1.226-234.2004

Otto, K., Norbeck, J., Larsson, T., Karlsson, K.-A., and Hermansson, M. (2001). Adhesion of type 1-fimbriated *Escherichia coli* to abiotic surfaces leads to altered composition of outer membrane proteins. *J. Bacteriol.* 183, 2445–2453. doi: 10.1128/JB.183.8.2445-2453.2001

Pautsch, A., and Schulz, G. E. (1998). Structure of the outer membrane protein A transmembrane domain. *Nat. Struct. Mol. Biol.* 5, 1013–1017. doi: 10.1038/2983

Pautsch, A., and Schulz, G. E. (2000). High-resolution structure of the OmpA membrane domain. *J. Mol. Biol.* 298, 273–282. doi: 10.1006/jmbi.2000.3671

Pond, K. (2005). *Water Recreation and Disease.* London; Seattle, WA: International Water Association.

Power, M. L., Ferrari, B. C., Littlefield-Wyer, J., Gordon, D. M., Slade, M. B., and Veal, D. A. (2006). A naturally occurring novel allele of *Escherichia coli* outer membrane protein A reduces sensitivity to bacteriophage. *Appl. Environ. Microbiol.* 72, 7930–7932. doi: 10.1128/AEM.01040-06

Prasadarao, N. V., Wass, C. A., Weiser, J. N., Stins, M. F., Huang, S. H., and Kim, K. S. (1996). Outer membrane protein A of *Escherichia coli* contributes to invasion of brain microvascular endothelial cells. *Infect. Immun.* 64, 146–153.

Pratt, L. A., and Kolter, R. (1998). Genetic analysis of *Escherichia coli* biofilm formation: roles of flagella, motility, chemotaxis and type I pili. *Mol. Microbiol.* 30, 285–293. doi: 10.1046/j.1365-2958.1998.01061.x

Rijnaarts, H. H. M., Norde, W., Lyklema, J., and Zehnder, A. J. B. (1999). DLVO and steric contributions to bacterial deposition in media of different ionic strengths. *Colloids Surf. B. Biointerfaces* 14, 179–195. doi: 10.1016/S0927-7765(99)00035-1

Rivas, L., Dykes, G. A., and Fegan, N. (2006). Attachment of shiga toxigenic *Escherichia coli* to beef muscle and adipose tissue. *J. Food Protect.* 69, 999–1006. doi: 10.4315/0362-028X-69.5.999

Rivas, L., Fegan, N., and Dykes, G. A. (2007). Attachment of Shiga toxigenic *Escherichia coli* to stainless steel. *Int. J. Food Microbiol.* 115, 89–94. doi: 10.1016/j.ijfoodmicro.2006.10.027

Rivas, L., Fegan, N., and Dykes, G. A. (2008). Expression and putative roles in attachment of outer membrane proteins of *Escherichia coli* O157 from planktonic and sessile culture. *Foodborne Pathog. Dis.* 5, 155–164. doi: 10.1089/fpd.2007.0052

Rosen, B. H. (2001). *Waterborne Pathogens in Agricultural Watersheds*. Ithaca, NY: USDA; Agriculture & N.R.C. Service.

Rosenbaum, J. L., and Child, F. M. (1967). Flagellar regeneration in protozoan flagellates. *J. Cell Biol.* 34, 345–364. doi: 10.1083/jcb.34.1.345

Schillinger, J. E., and Gannon, J. J. (1985). Bacterial adsorption and suspended particles in urban stormwater. *J. (Water Pollution Control Federation)* 57, 384–389.

Schweizer, M., Hindennach, I., Garten, W., and Henning, U. (1978). Major proteins of *Escherichia coli* outer cell-envelope membrane - interaction of protein IIStar with lipopolysaccharide. *Euro. J. Biochem.* 82, 211–217. doi: 10.1111/j.1432-1033.1978.tb12013.x

Smith, S. G. J., Mahon, V., Lambert, M. A., and Fagan, R. P. (2007). A molecular Swiss army knife: OmpA structure, function and expression. *FEMS Microbiol. Lett.* 273, 1–11. doi: 10.1111/j.1574-6968.2007.00778.x

Song, L., Johnson, P. R., and Elimelech, M. (1994). Kinetics of colloid deposition onto heterogeneously charged surfaces in porous-meida. *Environ. Sci. Technol.* 28, 1164–1171. doi: 10.1021/es00055a030

Sonntag, I., Schwarz, H., Hirota, Y., and Henning, U. (1978). Cell envelope and shape of *Escherichia coli*: multiple mutants missing the outer membrane lipoprotein and other major outer membrane proteins. *J. Bacteriol.* 136, 280–285.

Soupir, M. L., Mostaghimi, S., and Love, N. G. (2008). Method to partition between attached and unattached *E. coli* in runoff from agricultural lands. *J. Am. Water Resour. Assoc.* 44, 1591–1599. doi: 10.1111/j.1752-1688.2008.00262.x

Terrio, P. J. (1996). *Analysis of Suspended-Sediment Concentrations and Discharges at Four Long-Term Sediment Stations in Central And Southern Illinois, 1975–92 Water Years*. Urbana, IL: US Geological Survey.

USDA (2016). *Crop Production 2015 Summary*. Washington, DC: United States Department of Agriculture; National Agricultural Statistics Services.

Van Houdt, R., and Michiels, C. W. (2005). Role of bacterial cell surface structures in *Escherichia coli* biofilm formation. *Res. Microbiol.* 156, 626–633. doi: 10.1016/j.resmic.2005.02.005

Vidal, O., Longin, R., Prigent-Combaret, C., Dorel, C., Hooreman, M., and Lejeune, P. (1998). Isolation of an *Escherichia coli* K-12 mutant strain able to form biofilms on inert surfaces: involvement of a new ompR allele that increases curli expression. *J. Bacteriol.* 180, 2442–2449.

Vogel, H., and Jähnig, F. (1986). Models for the structure of outer-membrane proteins of *Escherichia coli* derived from Raman-Spectroscopy and prediction methods. *J. Mol. Biol.* 190, 191–199. doi: 10.1016/0022-2836(86)90292-5

Wan, J. M., Tokunaga, T. K., and Tsang, C. F. (1995). Bacterial sedimentation through a porous medium. *Water Resour. Res.* 31, 1627–1636. doi: 10.1029/95WR01255

Wang, Y. (2002). The function of OmpA in *Escherichia coli*. *Biochem. Biophys. Res. Commun.* 292, 396–401. doi: 10.1006/bbrc.2002.6657

Weiser, J. N., and Gotschlich, E. C. (1991). Outer-Membrane Protein-a (OmpA) contributes to serum resistance and pathogenicity of *Escherichia coli* K-1. *Infect. Immun.* 59, 2252–2258.

Weiss, B. L., Wu, Y., Schwank, J. J., Tolwinski, N. S., and Aksoy, S. (2008). An insect symbiosis is influenced by bacterium-specific polymorphisms in outer-membrane protein A. *Proc. Natl. Acad. Sci. U.S.A.* 105, 15088–15093. doi: 10.1073/pnas.0805666105

Yue, M., Han, X., De Masi, L., Zhu, C., Ma, X., Zhang, J., et al. (2015). Allelic variation contributes to bacterial host specificity. *Nat. Commun.* 6:8754. doi: 10.1038/ncomms9754

Zhao, W., Walker, S. L., Huang, Q., and Cai, P. (2014). Adhesion of bacterial pathogens to soil colloidal particles: influences of cell type, natural organic matter, and solution chemistry. *Water Res.* 53, 35–46. doi: 10.1016/j.watres.2014.01.009

Zwonitzer, M. R., Soupir, M. L., Jarboe, L. R., and Smith, D. R. (2016). Quantifying attachment and antibiotic resistance of *Escherichia coli* from conventional and organic swine manure. *J. Environ. Qual.* 45, 609–617. doi: 10.2134/jeq2015.05.0245

**Conflict of Interest Statement:** The authors declare that the research was conducted in the absence of any commercial or financial relationships that could be construed as a potential conflict of interest.

*Copyright © 2017 Liao, Liang, Yang, Soupir, Howe, Thompson and Jarboe. This is an open-access article distributed under the terms of the Creative Commons Attribution License (CC BY). The use, distribution or reproduction in other forums is permitted, provided the original author(s) or licensor are credited and that the original publication in this journal is cited, in accordance with accepted academic practice. No use, distribution or reproduction is permitted which does not comply with these terms.*
